# Base-Catalyzed Reaction of Isatins and (3-Hydroxyprop-1-yn-1-yl)phosphonates as a Tool for the Synthesis of Spiro-1,3-dioxolane Oxindoles with Anticancer and Anti-Platelet Properties

**DOI:** 10.3390/molecules29194764

**Published:** 2024-10-08

**Authors:** Arina V. Murashkina, Andrei V. Bogdanov, Alexandra D. Voloshina, Anna P. Lyubina, Alexandr V. Samorodov, Alexander Y. Mitrofanov, Irina P. Beletskaya, Elena A. Smolyarchuk, Kseniya A. Zavadich, Zulfiya A. Valiullina, Kseniya A. Nazmieva, Vladislav I. Korunas, Irina D. Krylova

**Affiliations:** 1 Department of Chemistry, M. V. Lomonosov Moscow State University, 119991 Moscow, Russia; arinamr@mail.ru (A.V.M.); beletska@org.chem.msu.ru (I.P.B.); 2 A. M. Butlerov Institute of Chemistry, Kazan Federal University, 420008 Kazan, Russia; 3 Arbuzov Institute of Organic and Physical Chemistry, FRC Kazan Scientific Center, Russian Academy of Sciences, 420088 Kazan, Russia; microbi@iopc.ru (A.D.V.); aplyubina@gmail.com (A.P.L.); 4 Department of Pharmacology, Bashkir State Medical University, 450008 Ufa, Russia; avsamorodov@gmail.com (A.V.S.); z_suleimanova@mail.ru (Z.A.V.); nazmievaksenia@gmail.com (K.A.N.); bsmu.korunas@gmail.com (V.I.K.); i.krylova16@yandex.ru (I.D.K.); 5 The A.P. Nelyubin Institute of Pharmacy, Sechenov First Moscow State Medical University (Sechenov University), 119571 Moscow, Russia; smolyarchuk_e_a@staff.sechenov.ru (E.A.S.); zavadich_k_a@staff.sechenov.ru (K.A.Z.)

**Keywords:** spiro compounds, isatin, phosphonates, cytotoxicity, anticoagulants, stereoselectivity

## Abstract

An approach to the synthesis of phosphoryl substituted spiro-1,3-dioxolane oxindoles was developed from the base-catalyzed reaction of various isatins with (3-hydroxyprop-1-yn-1-yl)phosphonates. It was found that various aryl-substituted and N-functionalized isatins with the formation of appropriate products with high yields and stereoselectivity when using *t*-BuOLi are able to react. Cytotoxic activity evaluation suggests that the most significant results in relation to the HuTu 80 cell line were shown by N-benzylated spirodioxolanes. 5-Cloro-*N*-unsubstituted spirooxindoles exhibit antiaggregational activity exceeding the values of acetylsalicylic acid.

## 1. Introduction

Spirooxindoles have a unique three-dimensional structure and are a common fragment in many natural products and pharmacologically significant compounds. A lot of diverse spirooxindoles containing various hetero- and carbocyclic fragments have been obtained to date using various multicomponent metal- and organocatalytic reactions of isatins and their derivatives [1,2,3,4,5]. The 1,3-dioxolane fragment is also often found in natural sources and presents in various synthetic bioactive structures. Despite this, spiroxindoles containing the 1,3-dioxolane fragment have been studied significantly less [6,7,8,9,10], although they have psychotropic [11], anticonvulsant [12], anxiolytic [13], and sedative–hypnotic activities [14].

The heterocyclic core of isatin features a privileged scaffold [15] and is a convenient building block for the creation of a hybrid system in view of the easy functionalization of the carbonyl group, aromatic moiety, and nitrogen atom [16,17,18,19,20,21]; its numerous derivatives possess a wide range of biological activity [22,23,24,25,26,27,28,29,30,31,32,33,34,35,36,37,38,39,40,41,42,43]. Due to their synthetic and commercial availability, isatins have been in the focus of attention as convenient starting compounds for the synthesis of spirooxindoles [44,45].

Phosphonic acids and their derivatives, due to their biological activity, are widely used in medicine and agriculture and are actively used in coordination chemistry and materials chemistry. In this regard, the introduction of phosphoryl substituents into complex bioactive molecules can significantly change their properties and lead to the production of new, practically useful compounds. In particular, several examples of spirooxindoles containing a phosphoryl group, along with aziridine [46,47], oxirane [48,49], cyclopropyl [50], pyrazoline [51], and cyclohexene [52,53] rings, are known in the literature, but compounds with a 1,3-dioxolane fragment have not yet been obtained. Therefore, in this work, we decided to obtain hybrid spirooxindoles that combine in one molecule both a phosphoryl group and a 1,3-dioxolane fragment. It is worth noting that approaches in the literature use indole derivatives such as substituted methyleneoxindoles, whereas the use of available isatins as starting compounds seems more desirable [46,47,48,49,50,51].

**Figure 1:**
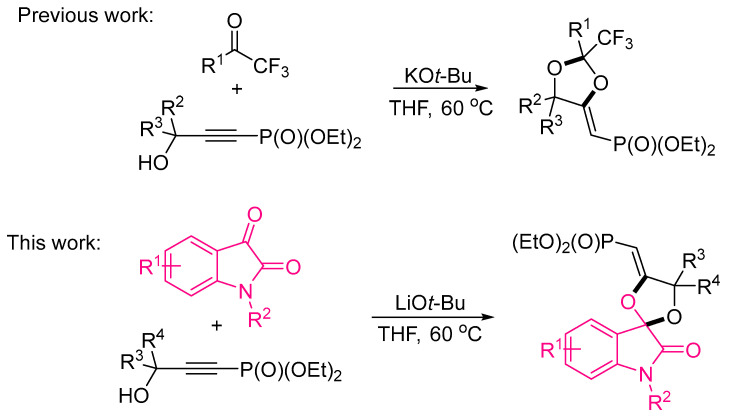
Synthesis of phosphoryl-substituted 1,3-dioxolane derivatives.

We have previously shown that compounds of the (3-hydroxyprop-1-yn-1-yl)phosphonate class can be used in a base-catalyzed reaction with activated trifluoromethyl ketones to obtain (1,3-dioxolan-4-ylidene)methylphosphonates in high yields [54]. In this work, we proposed the use of (3-hydroxyprop-1-yn-1-yl)phosphonates to obtain spirooxindoles by reaction with isatins, which also have an activated carbonyl group at position 3 (Figure 1).

## 2. Results

### 2.1. Synthesis

Based on previous results of the synthesis of 1,3-dioxolane derivatives using (3-hydroxypropynyl)phosphonates, we assumed that in this case, their reaction with isatins could also proceed in the presence of a base as a catalyst. Indeed, the model reaction of unsubstituted isatin **1a** with phosphonate **2a** proceeded within 16 h using 20 mol% *t*-BuOK as a base in THF to form the corresponding spirooxindole **3a** with an isomer ratio *Z*/*E* = 93/7 (entry 1, Table 1). At room temperature, the reaction practically did not proceed (entry 2). The use of other solvents (entries 3–6), as well as inorganic (potassium and cesium carbonates) and organic bases (entries 7–10), did not result in an increase in stereoselectivity. At the same time, the use of *t*-BuONa as a base led to an increase in stereoselectivity (entry 11), and the use of *t*-BuOLi made it possible to significantly reduce the reaction time (up to 4 h) and also led to the formation of spirooxindole **3a** with almost complete stereoselectivity (entry 12).

**Scheme 1:**
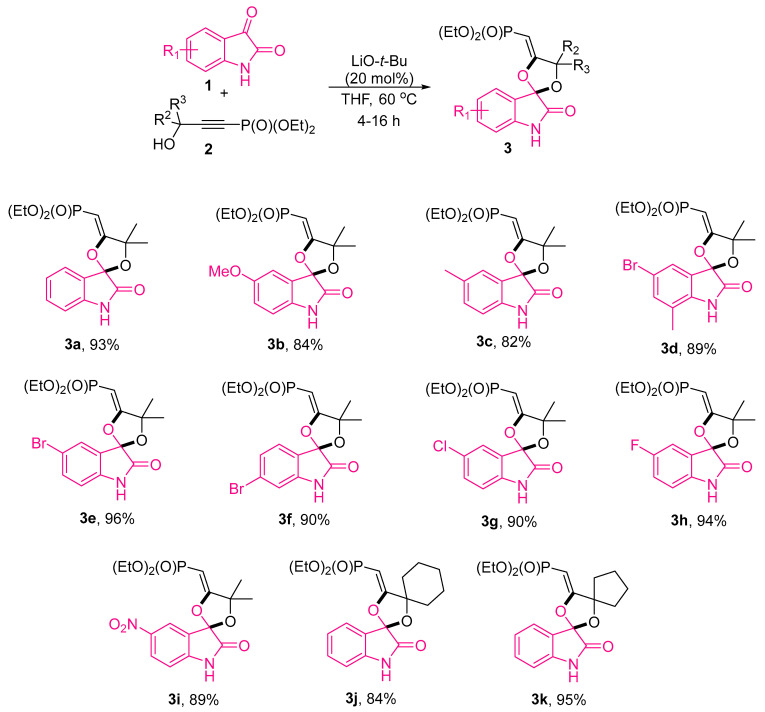
Synthesis of spirooxindoles **3**.

Under the optimized conditions found, a series of different substituted spirooxindoles were obtained with high yields and selectivity (Scheme 1, Supporting Information). The presence of substituents in the starting isatins (donor or acceptor) did not affect the yield and stereoselectivity, but required an increase in the reaction time. The reaction could also be performed with 3-hydroxypropynylphosphonate **2b** and **2c** containing a cyclohexane and cyclopentane fragment, leading to the corresponding dispirooxindoles **3j** and **3k**.

**Scheme 2:**
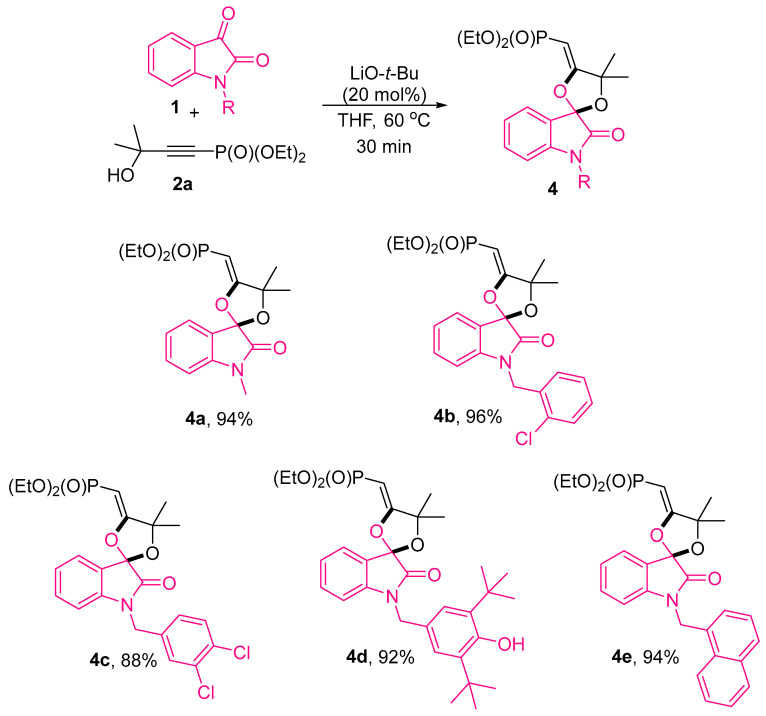
Synthesis of N-substituted spirooxindoles **4**.

The reactivity of N-substituted isatins turned out to be significantly higher than unsubstituted ones. Thus, the reaction of *N*-methyl- and various *N*-benzylisatins in the presence of 20 mol% *t*-BuOLi ended in half an hour and led to *N*-substituted spirooxindoles **4** in high yields (Scheme 2).

The structure of the compounds was confirmed by ^1^H, ^13^C, ^31^P NMR, and high-resolution mass spectra. For example, compound **3a** had an olefinic proton with a chemical shift of 4.52 (^2^*J_H,P_* = 7.1 Hz) ppm, which is in good agreement with known similar structures [54]. Also, the chemical shifts of carbon C1 and C3 (79.3 (d, ^1^*J_C,P_* = 196.8 Hz) and 86.1 (d, ^3^*J_C,P_* = 15.4 Hz) correspond to literature data of similar compounds.

As we have shown earlier using the example of the reaction of ethynylphosphonate with trifluoromethyl ketones, it is possible to carry out a three-component reaction with activated ketones with the intermediate formation of 3-hydroxyprop-1-yn-1-ylphosphonate [54]. However, in this case, ethynylphosphonate was inactive in the reaction with isatins and did not lead to the production of bis-spirooxindoles, in contrast to the previously published work [6] devoted to the reaction of propiolic acid esters with isatins.

**Figure 2:**
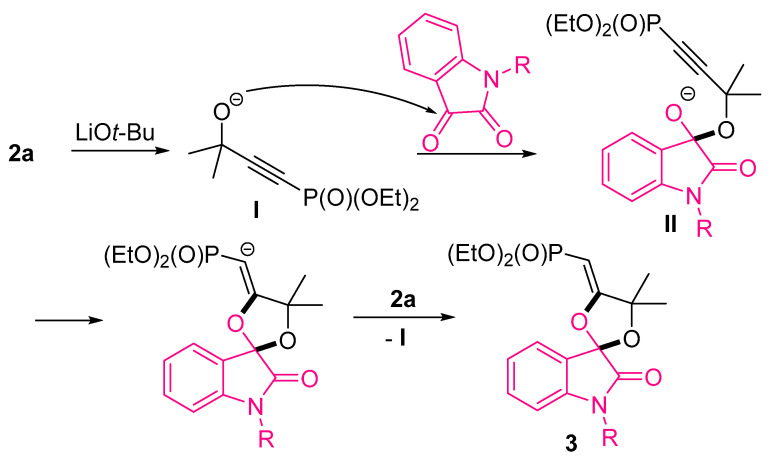
Proposed mechanism for the formation of **3**.

The proposed mechanism for the formation of spirooxindoles is shown in Figure 2. In the presence of a base, alcoholate **I** was generated, which added to the carbonyl group of isatin to form intermediate **II**, which then underwent cyclization to form **3**.

### 2.2. Bioactivity

#### 2.2.1. Anticancer Activity

The compounds were tested for cytotoxicity against cancer and normal cell lines (Table 2). Cytotoxic activity data are presented as IC_50_ values.

The studied compounds showed moderate activity against human cervical carcinoma (M-HeLa) and duodenal adenocarcinoma (HuTu 80) cancer cell lines and, in some cases, demonstrated low cytotoxicity against normal liver cells. The most significant results in relation to the HuTu 80 cell line were shown by benzylated analogs **4c** and **4d**, the IC_50_ values of which were 15.4 and 14.3, respectively. Both compounds were approximately 4 times more active than the reference drug 5-fluorouracil.

The selectivity of compounds for cancer cells is an important criterion for assessing the cytotoxic effect. For this purpose, the selectivity index (SI) was calculated as the ratio between the IC_50_ value for normal cells and the IC_50_ value for cancer cells. The selectivity index values for the tested compounds are given in Table 2. It is evident that the highest selectivity towards the HuTu 80 cancer cell line was demonstrated by compound **4d**, which contained a sterically hindered phenolic fragment and whose SI value was 3.0. Compounds with SI ≥ 3 are generally considered selective [55]. According to these data, compound **4d** can be considered selective with respect to the HuTu 80 cell line. However, the reference drug 5-fluorouracil was inferior to **4d** in terms of selectivity.

#### 2.2.2. Anticoagulant and Antiaggregating Activities

Systemic hypercoagulation and the risk of thromboembolic complications in cancer patients have been well studied, and the concept of bidirectional pathways between cancer and the blood clotting system has been elucidated [56].

For example, the results of current epidemiological studies demonstrate a 9-fold increase in the risk of venous thromboembolism in people with cancer compared with people without cancer [57]. However, the risk of thrombosis is heterogeneous and largely depends on the individual prothrombotic risk profiles of patients [58]. In part, the risk of thrombosis depends on the main risk factors specific to the patient, including age, gender, and concurrent diseases, and it also depends on treatment [59,60]. This is why the presence of antiplatelet properties as a mechanism of action of potential antitumor agents is considered as a promising approach to the development of therapeutic agents to combat oncopathologies.

In this work, the anticoagulant and antiaggregation properties of spirooxindoles under study were investigated (Table 3).

The findings show that compound **3g** exhibited antiaggregational activity exceeding the values of acetylsalicylic acid (13.7 vs. 20.5 at *p* < 0.05). Compounds **3d**, **e**, **h**, **k**, and **4a** had an antiplatelet effect at the level of acetylsalicylic acid. However, one should note that all compounds, in addition to antiaggregational activity, lengthen the lag period, which characterizes the process of the release of endogenous agonists of aggregation from platelets. This effect is absent in acetylsalicylic acid, which indicates the potentially wide antithrombotic potential of the studied compounds. With respect to the coagulation link of hemostasis, these compounds showed an effect exclusively on the APTT index. Therefore, the resulting compounds have high potential as a scaffold for the development of effective anticoagulant and antiaggregation agents.

## 3. Materials and Methods

### 3.1. Cells and Materials

For the experiments, we used tumor cell cultures M-HeLa clone 11 (epithelioid carcinoma of the cervix, subline HeLa, clone M-HeLa), HuTu 80, human duodenal adenocarcinoma from the collection of the Institute of Cytology, Russian Academy of Sciences (St. Petersburg), and human liver cells (Chang liver) from the collection of the Research Institute of Virology of the Russian Academy of Medical Sciences (Moscow).

### 3.2. Cytotoxic Assay

The cytotoxic effect on cells was determined using the colorimetric method of cell proliferation—the MTT test. NADP-H-dependent cellular oxidoreductase enzymes can, under certain conditions, reflect the number of viable cells. These enzymes are able to reduce the tetrazolium dye (MTT)—3-(4,5-dimethylthiazol-2-yl)-2,5-diphenyl-tetrazolium bromide—to insoluble blue-violet formazan, which crystallizes inside the cell. The amount of formazan formed is proportional to the number of cells with active metabolism. Cells were seeded on a 96-well Nunc plate at a concentration of 5 × 10^3^ cells per well in a volume of 100 μL of medium and cultured in a CO_2_ incubator at 37 °C until a monolayer was formed. Then, the nutrient medium was removed and 100 µL amounts of solutions of the test drug in the given dilutions were added to the wells, which were prepared directly in the nutrient medium with the addition of 5% DMSO to improve solubility. After 24 h of incubation of the cells with the tested compounds, the nutrient medium was removed from the plates and 100 µL of the nutrient medium without serum with MTT at a concentration of 0.5 mg/mL was added and incubated for 4 h at 37 °C. Formazan crystals were added with 100 µL of DMSO to each well. Optical density was recorded at 540 nm on an Invitrologic microplate reader (Pharma, Russia). The experiments for all compounds were repeated three times.

### 3.3. Anticoagulant and Antiaggregation Activities Study

The in vitro experiments were performed using the blood of healthy male donors aged 18–24 years (total 78 donors). The study was approved by the Ethics Committee of Federal State Budgetary Educational Institution of Higher Education at the Bashkir State Medical University of the Ministry of Health of Russian Federation (No.1 dated 30 January 2024). Informed consent was obtained from all participants before blood sampling. The blood was collected from the cubital vein using the system of vacuum blood collection BD Vacutainer^®^ equipment (Becton, Dickinson and Company, Franklin Lakes, NJ, USA). A 3.8% sodium citrate solution in a 9:1 ratio was used as a venous blood stabilizer. The study of the effect on platelet aggregation was performed using the Born method [61] using the «AT-02» aggregometer (SPC Medtech, Moscow, Russia). The assessment of the antiplatelet activity of the studied compounds and reference preparations was started with the final concentration of 2 × 10^−3^ mol/L. Adenosine diphosphate (ADP; 20 μg/mL) and collagen (5 mg/mL) manufactured by Tehnologia-Standart Company, Barnaul, Russia, were used as inducers of aggregation. The study on the anticoagulant activity was performed by standard recognized clotting tests using the optical two-channel automatic analyzer of blood coagulation, Solar CGL 2110 (CJSC SOLAR, Minsk, Belarus). The following parameters were studied: activated partial thromboplastin time (APTT), prothrombin time (PT), and fibrinogen concentrations according to the Clauss method. The determination of the anticoagulant activity of the studied compounds and reference preparation was performed in a concentration of 5 × 10^−4^ g/mL using the reagents manufactured by Tehnologia-Standart Company (Barnaul, Russia) [62]. The results of the study of the anticoagulant and antiaggregation activities were processed using the statistical package Statistica 10.0 (StatSoft Inc., Tulsa, OK, USA). The Shapiro–Wilk test was used to check the normality of actual data distribution. The form of the distribution of the data obtained differed from the normal one; therefore, non-parametric methods were used for further analysis. The data were presented as medians and 25 and 75 percentiles. Analysis of variance was conducted using the Kruskal–Wallis test. A *p* value of 0.05 was considered statistically significant.

### 3.4. Chemistry: Synthesis of ***3*** and ***4***

#### 3.4.1. General Remarks

Unless otherwise specified, all reactions were performed under an air atmosphere. The following anhydrous solvents were distilled prior to use: THF was distilled from sodium using benzophenone as the indicator. Reagents were used as purchased, unless otherwise indicated. Alkynylphosphonates **2a**–**2c** were obtained according to the procedure in the literature [54]. Flash chromatography was performed on silica gel using petroleum ether and EtOAc as eluents. ^1^H, ^13^C, and ^31^P NMR spectra were recorded on a 400 MHz spectrometer. Chemical shifts (ppm) were recorded with the solvent signal as the internal standard (CHCl_3_, ^1^H NMR 7.26 ppm, ^13^C NMR 77.16 ppm). Chemical shifts are expressed in ppm, and J values are given in hertz. Mass spectra were obtained by ESI on an Orbitrap spectrometer.

#### 3.4.2. General Procedure for the Synthesis of Spiro-1,3-dioxolane Oxindoles **3** and **4**

A vial containing a Teflon-coated stir bar was charged with (3-hydroxyprop-1-yn-1-yl)phosphonate **2** (0.2 mmol), isatin **1** (0.22 mmol), *t*-BuOLi (3.2 mg, 0.04 mmol, 20 mol%), and THF (2 mL). The vial was closed with a Teflon screw cap and stirred at 60 °C for 0.5–16 h. After solvent removal and purification by flash chromatography on silica gel (petroleum ether/EtOAc, 1/1) products **3** and **4** were obtained as colorless oils, which partially solidified upon standing.

Diethyl (Z)-((4′,4′-dimethyl-2-oxospiro[indoline-3,2′-[1,3]dioxolan]-5′-ylidene)methyl)phosphonate (**3a**) (reaction time: 4 h); yield 66 mg (93%); ^1^H NMR (400 MHz, CDCl_3_) δ 9.44 (s, 1H), 7.14 (d, ^3^*J_H,H_* = 7.0 Hz, 1H), 7.07–6.99 (m, 1H), 6.89–6.82 (m, 1H), 6.63 (d, ^3^*J_H,H_* = 7.6 Hz, 1H), 4.52 (d, ^2^*J_H,P_* = 7.1 Hz, 1H), 4.15–3.96 (m, 4H), 1.79 (s, 3H), 1.63 (s, 3H), 1.30 (t, ^3^*J_H,H_* = 7.1 Hz, 3H), 1.27 (t, ^3^*J_H,H_* = 7.1 Hz, 3H); ^13^C NMR (101 MHz, CDCl_3_) δ 173.7, 171.7, 143.0, 132.4, 124.8, 122.7, 122.5, 111.5, 106.0, 86.1 (d, ^3^*J_C,P_* = 15.4 Hz), 79.3 (d, ^1^*J_C,P_* = 196.8 Hz), 62.3 (d, ^2^*J_C,P_* = 4.2 Hz), 61.9 (d, ^2^*J_C,P_* = 4.2 Hz), 30.1, 28.8, 16.3; ^31^P NMR (162 MHz, CDCl_3_) δ 17.28; HRMS (ESI): *m*/*z* calcd for C_17_H_24_NO_5_P+H^+^: 354.1470 [M+H]^+^; found: 354.1477.

Diethyl (Z)-((5′-methoxy-4,4-dimethyl-2′-oxospiro[[1,3]dioxolane-2,3′-indolin]-5-ylidene)methyl)phosphonate (**3b**) (reaction time: 16 h); yield: 66 mg (84%); ^1^H NMR (400 MHz, CDCl_3_) δ 9.47 (s, 1H), 6.67 (s, 1H), 6.53 (s, 2H), 4.49 (d, ^2^*J_H,P_* = 7.2 Hz, 1H), 4.16–4.04 (m, 4H), 3.71 (s, 3H), 1.78 (s, 3H), 1.62 (s, 3H), 1.31 (t, ^3^*J_H,H_* = 7.1 Hz, 3H), 1.29 (t, ^3^*J_H,H_* = 7.1 Hz, 3H); ^13^C NMR (101 MHz, CDCl_3_) δ 173.9, 171.5, 136.4, 123.3, 117.0, 112.2, 111.3, 106.2, 86.0 (d, ^3^*J_C,P_* = 15.6 Hz), 79.2 (d, ^1^*J_C,P_* = 196.9 Hz), 62.3 (d, ^2^*J_C,P_* = 5.3 Hz), 61.7 (d, ^2^*J_C,P_* = 5.3 Hz), 55.8, 30.1, 28.8, 16.4; ^31^P NMR (162 MHz, CDCl_3_) δ 17.43; HRMS (ESI): *m*/*z* calcd for C_18_H_26_NO_6_P+H^+^: 384.1576 [M+H]^+^; found: 384.1574.

Diethyl (Z)-((4,4,5′-trimethyl-2′-oxospiro[[1,3]dioxolane-2,3′-indolin]-5-ylidene)methyl)phosphonate (**3c**) (reaction time: 16 h); yield: 62 mg (82%); ^1^H NMR (400 MHz, CDCl_3_) δ 9.40 (s, 1H), 6.92 (m, 1H), 6.80 (t, ^3^*J_H,H_* = 7.9 Hz, 1H), 6.50 (d, ^3^*J_H,H_* = 7.9, 1H), 4.50 (d, ^2^*J_H,P_* = 7.3 Hz, 1H), 4.16–4.05 (m, 4H), 2.21 (s, 3H), 1.79 (s, 3H), 1.64 (s, 3H), 1.32 (t, ^3^*J_H,H_* = 6.9 Hz, 3H), 1.30 (t, ^3^*J_H,H_* = 6.9 Hz, 3H); ^13^C NMR (101 MHz, CDCl_3_) δ 173.8, 171.6, 140.6, 132.3, 131.9, 125.3, 122.3, 111.4, 106.2, 85.9 (d, ^3^*J_C,P_* = 16.0 Hz), 79.1 (d, ^1^*J_C,P_* = 196.8 Hz), 62.2 (d, ^2^*J_C,P_* = 5.3 Hz), 61.7 (d, ^2^*J_C,P_* = 5.3 Hz), 30.1, 28.8, 21.0, 16.3; ^31^P NMR (162 MHz, CDCl_3_) δ 17.49; HRMS (ESI): *m*/*z* calcd for C_18_H_26_NO_5_P+H^+^: 368.1627 [M+H]^+^; found: 368.1637.

Diethyl (Z)-((5′-bromo-4,4,7′-trimethyl-2′-oxospiro[[1,3]dioxolane-2,3′-indolin]-5-ylidene)methyl)phosphonate (**3d**) (reaction time: 16 h); yield: 82 mg (89%); ^1^H NMR (400 MHz, CDCl_3_) δ 10.49 (s, 1H), 7.00 (m, 2H), 4.39 (d, ^2^*J_H,P_* = 5.8 Hz, 1H), 4.28–4.04 (m, 4H), 1.99 (s, 3H), 1.76 (s, 3H), 1.61 (s, 3H), 1.32 (t, ^3^*J_H,H_* = 6.9 Hz, 3H), 1.31 (t, ^3^*J_H,H_* = 6.9 Hz, 3H); ^13^C NMR (101 MHz, CDCl_3_) δ 174.3, 171.0, 141.5, 136.3, 124.88, 123.3, 122.7, 114.2, 105.5, 86.3 (d, ^3^*J_C,P_* = 15.8 Hz), 79.1 (d, ^1^*J_C,P_* = 197.7 Hz), 62.5 (d, ^2^*J_C,P_* = 5.3 Hz), 61.6 (d, ^2^*J_C,P_* = 5.3 Hz), 30.2, 28.9, 16.4; ^31^P NMR (162 MHz, CDCl_3_) δ 16.66; HRMS (ESI): *m*/*z* calcd for C_18_H_25_BrNO_5_P+H^+^: 446.0732 [M+H]^+^; found: 446.0724.

Diethyl (Z)-((5′-bromo-4,4-dimethyl-2′-oxospiro[[1,3]dioxolane-2,3′-indolin]-5-ylidene)methyl)phosphonate (**3e**) (reaction time: 16 h); yield: 86 mg (96%); ^1^H NMR (400 MHz, CDCl_3_) δ 10.22 (s, 1H), 7.18–7.05 (m, 2H), 6.40 (d, ^3^*J_H,H_* = 8.7 Hz, 1H), 4.45 (d, ^2^*J_H,P_* = 6.6 Hz, 1H), 4.18–4.05 (m, 4H), 1.77 (s, 3H), 1.62 (s, 3H), 1.33 (t, ^3^*J_H,H_* = 7.0 Hz, 3H), 1.31 (t, ^3^*J_H,H_* = 7.2 Hz, 3H); ^13^C NMR (101 MHz, CDCl_3_) δ 174.1, 170.7, 142.5, 134.7, 127.6, 123.9, 114.7, 113.4, 105.4, 86.9 (d, ^3^*J_C,P_* = 16.0 Hz), 79.3 (d, ^1^*J_C,P_* = 198.3 Hz), 62.4 (d, ^2^*J_C,P_* = 5.2 Hz), 61.8 (d, ^2^*J_C,P_* = 5.3 Hz), 30.0, 28.8, 16.4; ^31^P NMR (162 MHz, CDCl_3_) δ 17.27; HRMS (ESI): *m*/*z* calcd for C_17_H_23_BrNO_5_P+H^+^: 432.0575 [M+H]^+^; found: 432.0580.

Diethyl (Z)-((6′-bromo-4,4-dimethyl-2′-oxospiro[[1,3]dioxolane-2,3′-indolin]-5-ylidene)methyl)phosphonate (**3f**) (reaction time: 16 h); yield: 78 mg (90%); ^1^H NMR (400 MHz, CDCl_3_) δ 10.22 (s, 1H), 7.02 (d, ^3^*J_H,H_* = 9.4 Hz, 1H), 6.95 (d, ^3^*J_H,H_* = 7.9 Hz, 1H), 6.65 (m, 1H), 4.47 (d, ^2^*J_H,P_* = 6.7 Hz, 1H), 4.21–4.06 (m, 4H), 1.80 (s, 3H), 1.64 (s, 3H), 1.37 (t, ^3^*J_H,H_* = 7.1 Hz, 3H), 1.34 (t, ^3^*J_H,H_* = 6.9 Hz, 3H); ^13^C NMR (101 MHz, CDCl_3_) δ 174.3, 171.0, 144.5, 126.2, 125.6, 125.5, 121.0, 115.0, 105.5, 86.2 (d, ^3^*J_C,P_* = 16.1 Hz), 79.1 (d, ^1^*J_C,P_* = 197.2 Hz), 62.4 (d, ^2^*J_C,P_* = 5.3 Hz), 61.8 (d, ^2^*J_C,P_* = 5.3 Hz), 30.0, 28.7, 16.4; ^31^P NMR (162 MHz, CDCl_3_) δ 17.45; HRMS (ESI): *m*/*z* calcd for C_17_H_23_BrNO_5_P+H^+^: 432.0575 [M+H]^+^; found: 432.0584.

Diethyl (Z)-((5′-chloro-4,4-dimethyl-2′-oxospiro[[1,3]dioxolane-2,3′-indolin]-5-ylidene)methyl)phosphonate (**3g**) (reaction time: 16 h); yield: 72 mg (90%); ^1^H NMR (400 MHz, CDCl_3_) δ 10.15 (s, 1H), 7.00 (s, Hz, 1H), 6.96 (d, ^3^*J_H,H_* = 8.1 Hz, 1H), 6.47 (d, ^3^*J_H,H_* = 8.4 Hz, 1H), 4.47 (d, ^2^*J_H,P_* = 6.5 Hz, 1H), 4.18–4.06 (m, 4H), 1.78 (s, 3H), 1.63 (s, 3H), 1.37–1.31 (m, 6H); ^13^C NMR (101 MHz, CDCl_3_) δ 174.0, 170.9, 141.9, 131.9, 127.5, 124.9, 123.6, 112.9, 105.5, 86.3 (d, ^3^*J_C,P_* = 16.3 Hz), 79.3 (d, ^1^*J_C,P_* = 197.4 Hz), 62.4 (d, ^2^*J_C,P_* = 5.3 Hz), 61.8 (d, ^2^*J_C,P_* = 5.3 Hz), 30.0, 28.7, 16.3; ^31^P NMR (162 MHz, CDCl_3_) δ 17.27; HRMS (ESI): *m*/*z* calcd for C_17_H_23_ClNO_5_P+H^+^: 388.1081 [M+H]^+^; found: 388.1072.

Diethyl (Z)-((5′-fluoro-4,4-dimethyl-2′-oxospiro[[1,3]dioxolane-2,3′-indolin]-5-ylidene)methyl)phosphonate (**3h**) (reaction time: 16 h); yield: 73 mg (94%); ^1^H NMR (400 MHz, CDCl_3_): δ 9.87 (s, 1H), 6.81–6.78 (m, 1H), 6.72–6.66 (m, 1H), 6.54–6.51 (m, 1H), 4.48 (d, ^2^*J_H,P_* = 6.7 Hz, 1H), 4.20–4.07 (m, 4H), 1.80 (s, 3H), 1.63 (s, 3H), 1.36–1.31 (m, 6H); ^13^C NMR (101 MHz, CDCl_3_): δ 174.0, 171.2, 158.7 (d, ^1^*J_C,F_* = 241.0 Hz), 139.3, 123.5 (d, ^3^*J_C,F_* = 6.9 Hz), 118.4 (d, ^2^*J_C,F_* = 23.2 Hz), 112.6 (d, ^3^*J_C,F_* = 7.1 Hz), 112.6 (d, ^2^*J_C,F_* = 24.6 Hz), 105.7, 86.3 (d, ^3^*J_C,P_* = 16.0 Hz), 79.4 (d, ^1^*J_C,P_* = 197.5 Hz), 62.4 (d, ^2^*J_C,P_* = 4.6 Hz), 61.8 (d, ^2^*J_C,P_* = 5.6 Hz), 30.1, 28.8, 16.4 (d, ^3^*J_C,P_* = 6.5 Hz); ^31^P NMR (162 MHz, CDCl_3_) δ 17.26; HRMS (ESI): *m*/*z* calcd for C_17_H_23_FNO_5_P+H^+^: 372.1376 [M+H]^+^; found: 372.1370.

Diethyl (Z)-((4,4-dimethyl-5′-nitro-2′-oxospiro[[1,3]dioxolane-2,3′-indolin]-5-ylidene)methyl)phosphonate (**3i**) (reaction time: 16 h); yield: 76 mg (89%); ^1^H NMR (400 MHz, CDCl_3_) δ 11.00 (s, 1H), 7.92 (d, 1H), 7.79 (m, 1H), 6.69 (d, ^3^*J_H,H_* = 8.6 Hz, 1H), 4.54 (d, ^2^*J_H,P_* = 6.3 Hz, 1H), 4.20–4.09 (m, 4H), 1.78 (s, 3H), 1.68 (s, 3H), 1.39–1.33 (m, 6H); ^13^C NMR (101 MHz, CDCl_3_) δ 173.8, 171.2, 149.6, 143.0, 128.2, 123.2, 120.9, 111.7, 104.4, 86.7 (d, ^3^*J_C,P_* = 16.2 Hz), 80.0 (d, ^1^*J_C,P_* = 198.0 Hz), 62.5 (d, ^2^*J_C,P_* = 5.3 Hz), 62.2 (d, ^2^*J_C,P_* = 5.3 Hz), 30.0, 28.7, 16.4; ^31^P NMR (162 MHz, CDCl_3_) δ 16.73; HRMS (ESI): *m*/*z* calcd for C_17_H_23_N_2_O_7_P+H^+^: 399.1321 [M+H]^+^; found: 399.1329.

Diethyl (Z)-[(2″-oxo-1″,2″-dihydrodispiro[cyclohexane-1,4′-[1,3]dioxolane-2′,3″-indol]-5′-ylidene)methyl]phosphonate (**3j**) (reaction time: 16 h); yield: 71 mg (84%); ^1^H NMR (400 MHz, CDCl_3_) δ 9.50 (s, 1H), 7.10 (d, ^3^*J_H,H_* = 7.6 Hz, 1H), 7.01 (t, ^3^*J_H,H_* = 7.7 Hz, 1H), 6.83 (t, ^3^*J_H,H_* = 7.5 Hz, 1H), 6.62 (d, ^3^*J_H,H_* = 7.8 Hz, 1H), 4.49 (d, ^2^*J_H,P_* = 7.3 Hz, 1H), 4.15–4.01 (m, 4H), 2.63 (d, ^2^*J_H,H_* = 10.8 Hz, 1H), 1.98 (d, ^2^*J_H,H_* = 11.6 Hz, 1H), 1.68–1.51 (m, 7H), 1.32–1.25 (m, 7H); ^13^C NMR (101 MHz, CDCl_3_) δ 173.4, 171.7, 143.1, 132.3, 124.7, 122.8, 122.5, 111.5, 106.1, 87.6 (d, ^3^*J_C,P_* = 15.4 Hz), 79.3 (d, ^1^*J_C,P_* = 196.0 Hz), 62.1 (d, ^2^*J_C,P_* = 4.8 Hz), 61.8 (d, ^2^*J_C,P_* = 4.8 Hz), 38.8, 36.9, 24.9, 22.1, 22.0, 16.3; ^31^P NMR (162 MHz, CDCl_3_) δ 17.76; HRMS (ESI): *m*/*z* calcd for C_20_H_28_NO_5_P+H^+^: 394.1783 [M+H]^+^; found: 394.1781.

Diethyl (Z)-[(2″-oxo-1″,2″-dihydrodispiro[cyclopentane-1,4′-[1,3]dioxolane-2′,3″-indol]-5′-ylidene)methyl]phosphonate (**3k**) (reaction time: 16 h); yield: 77 mg (95%); ^1^H NMR (400 MHz, CDCl_3_) δ 9.60 (s, 1H), 7.13 (d, ^3^*J_H,H_* = 7.4 Hz, 1H), 7.00 (t, ^3^*J_H,H_* = 7.7 Hz, 1H), 6.82 (t, ^3^*J_H,H_* = 7.5 Hz, 1H), 6.62 (d, ^3^*J_H,H_* = 7.8 Hz, 1H), 4.53 (d, ^2^*J_H,P_* = 7.3 Hz, 1H), 4.16–4.01 (m, 4H), 2.67–2.63 (m, 1H), 2.21–2.17 (m, 1H), 2.09–1.99 (m, 2H), 1.82–1.77 (m, 4H), 1.29 (t, ^3^*J_H,H_* = 7.1 Hz, 3H), 1.26 (t, ^3^*J_H,H_* = 7.1, 3H); ^13^C NMR (101 MHz, CDCl_3_) δ 173.6, 171.4, 143.1, 132.3, 124.7, 122.5, 122.3, 111.5, 105.8, 95.4 (d, ^3^*J_C,P_* = 16.0 Hz), 78.7 (d, ^1^*J_C,P_* = 197.2 Hz), 62.1 (d, ^2^*J_C,P_* = 4.5 Hz), 61.8 (d, ^2^*J_C,P_* = 4.5 Hz), 42.6, 42.3, 25.1, 24.9, 16.3; ^31^P NMR (162 MHz, CDCl_3_) δ 17.73; HRMS (ESI): *m*/*z* calcd for C_19_H_26_NO_5_P+H^+^: 380.1627 [M+H]^+^; found: 380.1627.

Diethyl (Z)-((1,4′,4′-trimethyl-2-oxospiro[indoline-3,2′-[1,3]dioxolan]-5′-ylidene)methyl)phosphonate (**4a**) (reaction time: 30 min); yield: 69 mg (94%); ^1^H NMR (400 MHz, CDCl_3_) δ 7.40–7.34 (m, 1H), 7.28 (d, ^3^*J_H,H_* = 7.4 Hz, 1H), 7.10–7.03 (m, 1H), 6.78 (d, ^3^*J_H,H_* = 7.8 Hz, 1H), 4.56 (d, ^2^*J_H,P_* = 8.1 Hz, 1H), 4.07–3.94 (m, 4H), 3.09 (s, 3H), 1.78 (s, 3H), 1.62 (s, 3H), 1.24 (t, ^3^*J_H,H_* = 7.1 Hz, 3H), 1.20 (t, ^3^*J_H,H_* = 7.1 Hz, 3H); ^13^C NMR (101 MHz, CDCl_3_) δ 172.4, 170.4, 144.7, 132.7, 125.1, 123.6, 122.7, 109.0, 105.5, 86.1 (d, ^3^*J_C,P_* = 15.2 Hz), 80.1 (d, ^1^*J_C,P_* = 195.3 Hz), 62.0 (d, ^2^*J_C,P_* = 4.8 Hz), 61.8 (d, ^2^*J_C,P_* = 4.8 Hz), 30.0, 28.7, 26.2, 16.2 (d, ^3^*J_C,P_* = 5.6 Hz); ^31^P NMR (162 MHz, CDCl_3_) δ 16.82; HRMS (ESI): *m*/*z* calcd for C_18_H_26_NO_5_P+H^+^: 368.1627 [M+H]^+^; found: 368.1631.

Diethyl (Z)-((1′-(2-chlorobenzyl)-4,4-dimethyl-2′-oxospiro[[1,3]dioxolane-2,3′-indolin]-5-ylidene)methyl)phosphonate (**4b**) (reaction time: 30 min); yield: 95 mg (96%); ^1^H NMR (400 MHz, CDCl_3_) δ 7.36 (t, ^3^*J_H,H_* = 8.7 Hz, 2H), 7.27 (t, ^3^*J_H,H_* = 7.7 Hz, 1H), 7. 19 (t, ^3^*J_H,H_* = 7.7 Hz, 1H), 7.14 (t, ^3^*J_H,H_* = 7.5 Hz, 1H), 7.09–7.05 (m, 2H), 6.63 (d, ^3^*J_H,H_* = 7.9 Hz, 1H), 4.91 (s, 2H), 4.60 (d, ^2^*J_H,P_* = 7.5 Hz, 1H), 4.08–3.97 (m, 4H), 1.82 (s, 3H), 1.66 (s, 3H), 1.25 (t, ^3^*J_H,H_* = 7.1 Hz, 3H), 1.21 (t, ^3^*J_H,H_* = 7.1 Hz, 3H); ^13^C NMR (101 MHz, CDCl_3_) δ 171.9, 170.8, 143.5, 132.8, 132.7, 132.0, 129.8, 129.1, 127.7, 127.3, 125.3, 123.9, 122.7, 109.9, 105.4, 86.2 (d, ^3^*J_C,P_* = 15.1 Hz), 80.7 (d, ^1^*J_C,P_* = 195.3 Hz), 61.8 (d, ^2^*J_C,P_* = 5.3 Hz), 61.6 (d, ^2^*J_C,P_* = 5.3 Hz), 41.2, 30.0, 28.7, 16.3; ^31^P NMR (162 MHz, CDCl_3_) δ 16.42; HRMS (ESI): *m*/*z* calcd for C_24_H_29_ClO_5_P+H^+^: 478.1550 [M+H]^+^; found: 478.1549. 

Diethyl (Z)-((1′-(3,4-dichlorobenzyl)-4,4-dimethyl-2′-oxospiro[[1,3]dioxolane-2,3′-indolin]-5-ylidene)methyl)phosphonate (**4c**) (reaction time: 30 min); yield: 93 mg (88%); ^1^H NMR (400 MHz, CDCl_3_) δ 7.36–7.27 (m, 4H), 7.09–7.05 (m, 2H), 6.64 (d, ^3^*J_H,H_* = 7.8 Hz, 1H), 4.76 (d, ^2^*J_H,H_* = 15.8 Hz, 1H), 4.69 (d, ^2^*J_H,H_* = 15.9 Hz, 1H), 4.59 (d, ^2^*J_H,P_* = 7.4 Hz, 1H), 4.06–3.96 (m, 4H), 1.80 (s, 3H), 1.65 (s, 3H), 1.25–1.18 (m, 6H); ^13^C NMR (101 MHz, CDCl_3_) δ 171.8, 170.7, 143.3, 135.2, 133.0, 132.7, 132.1, 131.0, 129.2, 126.6, 125.5, 124.0, 122.7, 109.7, 105.3, 86.3 (d, ^3^*J_C,P_* = 15.1 Hz), 80.8 (d, ^1^*J_C,P_* = 195.3 Hz), 61.9 (d, ^2^*J_C,P_* = 5.3 Hz), 61.7 (d, ^2^*J_C,P_* = 5.3 Hz), 42.8, 30.0, 28.7, 16.3; ^31^P NMR (162 MHz, CDCl_3_) δ 16.33; HRMS (ESI): *m*/*z* calcd for C_24_H_28_Cl_2_O_5_P+H^+^: 512.1160 [M+H]^+^; found: 512.1166.

Diethyl (Z)-((1′-(3,5-di-tert-butyl-4-hydroxybenzyl)-4,4-dimethyl-2′-oxospiro[[1,3]dioxolane-2,3′-indolin]-5-ylidene)methyl)phosphonate (**4d**) (reaction time: 30 min); yield: 108 mg (92%); ^1^H NMR (400 MHz, CDCl_3_) δ 7.34–7.30 (m, 2H), 7.09 (s, 2H), 7.05 (t, ^3^*J_H,H_* = 7.5 Hz, 1H), 6.81 (d, ^3^*J_H,H_* = 7.8 Hz, 1H), 5.25 (s, 1H), 4.76 (d, ^2^*J_H,H_* = 15.3 Hz, 1H), 4.64 (d, ^2^*J_H,H_* = 15.6 Hz, 1H), 4.59 (s, 1H), 3.97–4.10 (s, 4H), 1.82 (s, 3H), 1.65 (s, 3H), 1.38 (s, 18H), 1.25–1.19 (m, 6H); ^13^C NMR (101 MHz, CDCl_3_) δ 172.2, 170.6, 153.4, 144.2, 136.4, 132.4, 125.6, 125.2, 124.4, 123.4, 122.9, 110.0, 105.5, 86.0 (d, ^3^*J_C,P_* = 15.7 Hz), 80.6 (d, ^1^*J_C,P_* = 190.7 Hz), 61.9, 61.7, 44.1, 34.3, 30.2, 30.1, 28.7, 16.3; ^31^P NMR (162 MHz, CDCl_3_) δ 16.51; HRMS (ESI): *m*/*z* calcd for C_32_H_46_NO_6_P+H^+^: 572.3141 [M+H]^+^; found: 572.3149.

Diethyl (Z)-((4,4-dimethyl-1′-(naphthalen-1-ylmethyl)-2′-oxospiro[[1,3]dioxolane-2,3′-indolin]-5-ylidene)methyl)phosphonate (**4e**) (reaction time: 30 min); yield: 95 mg (94%); ^1^H NMR (400 MHz, CDCl_3_) δ 8.05 (d, ^3^*J_H,H_* = 8.3 Hz, 1H), 7.89 (d, ^3^*J_H,H_* = 8.0 Hz, 1H), 7.79 (d, ^3^*J_H,H_* = 8.2 Hz, 1H), 7.59 (t, ^3^*J_H,H_* = 7.0 Hz, 1H), 7.53 (t, ^3^*J_H,H_* = 7.0 Hz, 1H), 7.39–7.35 (m, 2H), 7.27 (d, ^3^*J_H,H_* = 7.1 Hz, 1H), 7.22 (t, ^3^*J_H,H_* = 7.8 Hz, 1H), 7.07 (t, ^3^*J_H,H_* = 7.5 Hz, 1H), 6.62 (d, ^3^*J_H,H_* = 7.9 Hz, 1H), 5.33 (d, ^2^*J_H,H_* = 16.5 Hz, 1H), 5.28 (d, ^2^*J_H,H_* = 16.5 Hz, 1H), 4.64 (d, ^2^*J_H,P_* = 7.4 Hz, 1H), 4.12–3.99 (m, 4H), 1.88 (s, 3H), 1.70 (s, 3H), 1.30–1.23 (m, 6H); ^13^C NMR (101 MHz, CDCl_3_) δ 172.0, 170.8, 144.1, 133.8, 132.6, 130.8, 129.5, 129.0, 128.5, 126.7, 126.1, 125.32, 125.28, 124.0, 123.7, 122.8, 122.6, 110.3, 105.5, 86.2 (d, ^3^*J_C,P_* = 15.4 Hz), 80.7 (d, ^1^*J_C,P_* = 195.2 Hz), 61.9 (d, ^2^*J_C,P_* = 4.8 Hz), 61.7 (d, ^2^*J_C,P_* = 4.8 Hz), 41.9, 30.1, 28.7, 16.3; ^31^P NMR (162 MHz, CDCl_3_) δ 16.54; HRMS (ESI): *m*/*z* calcd for C_28_H_35_NO_5_P+H^+^: 494.2096 [M+H]^+^; found: 494.2091.

## 4. Conclusions

In conclusion, we have developed a convenient approach for the synthesis of phosphoryl-substituted spiro-1,3-dioxolane oxindoles by the *t*-BuOLi-catalyzed reaction of various available aryl- and 1-substituted isatins and (3-hydroxyprop-1-yn-1-yl)phosphonates. A large series of various spirooxindoles were obtained in high yields regardless of the nature of the substituents in isatins and phosphonates. The study of the cytotoxicity of new compounds has shown the high potential of spirooxindoles containing a phosphonate group in the search for selective antitumor drugs. In addition, this class of oxindole-based spiro-compounds has high potential as a scaffold for the development of effective anticoagulant and antiaggregation agents.

## Figures and Tables

**Table 1:** Optimization of reaction conditions for the synthesis of **3a** ^a^.

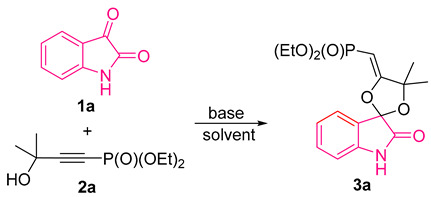						
Entry	Base (mol%)	Solvent	T, °C	t, hours	Yield (Conversion), %	*Z/E*
1	*t-*BuOK (20)	THF	60	16	100 (100)	93/7
2	*t-*BuOK (20)	THF	25	16	5 (5)	-
3	*t-*BuOK (20)	toluene	60	16	100 (100)	84/16
4	*t-*BuOK (20)	dioxane	60	16	85 (85)	91/9
5	*t-*BuOK (20)	MTBE	60	16	42 (42)	92/8
6	*t-*BuOK (20)	EtOH	60	16	95 (100)	87/13
7	Cs_2_CO_3_ (20)	THF	60	16	100 (100)	86/14
8	K_2_CO_3_ (20)	THF	60	24	93 (93)	91/9
9	DBU (20)	THF	60	24	88 (88)	91/9
10	TEA (20)	THF	60	24	0	-
11	*t*-BuONa (20)	THF	60	16	78 (78)	96/4
12	*t*-BuOLi (20)	THF	60	4	100 (100)	>99/1

^a^ Yields, conversion, and Z/E ratio were determined by ^1^H and ^31^P NMR spectroscopy.

**Table 2:** Cytotoxic effects (IC_50_, µM) and selectivity index (SI) of test compounds.

Cmpd	Cancer Cell Line				Normal Cell Line
	M-HeLa		HuTu 80		Chang Liver
	IC_50_	SI	IC_50_	SI	
**3b**	91.0 ± 7.3	1.2	118 ± 9.3	ns	108 ± 8.4
**3c**	102.6 ± 8.2	ns	180 ± 14.4	ns	84.5 ± 6.8
**3d**	81.0 ± 6.5	2.5	119 ± 9.5	1.7	200 ± 16
**3e**	86.1 ± 6.9	2.1	68.6 ± 5.5	2.7	185 ± 14.8
**3f**	76.0 ± 6.1	1.4	131 ± 10	ns	103 ± 8.2
**3g**	92.7 ± 7.4	1.3	113.3 ± 9	1.0	118 ± 9.3
**3h**	107.0 ± 8.7	1.6	110.7 ± 8.9	1.5	167 ± 13.3
**3i**	99.3 ± 7.8	1.3	136.2 ± 11	ns	125 ± 9.9
**3j**	84.5 ± 6.8	2.5	124 ± 10	1.7	208 ± 16.4
**3k**	92.8 ± 7.4	ns	107.3 ± 8.6	ns	61.2 ± 4.9
**4a**	104.0 ± 8.1	1.9	129.0 ± 10	1.5	195 ± 15
**4b**	29.0 ± 2.3	1.3	31.4 ± 2.5	1.2	39.0 ± 2.8
**4c**	23.4 ± 5.0	1.1	15.4 ± 1.3	1.6	25 ± 2
**4d**	30.5 ± 3.6	1.3	14.3 ± 0.4	3.0	40.0 ± 3.1
**4e**	23.5 ± 1.8	1.1	23.3 ± 1.7	1.1	26 ± 2.1
**3a**	na	-	na	-	nd
**3j**	na	-	na	-	nd
**5-fluorouracil**	75.4 ± 5.9	1.1	65.2 ± 5.6	1.3	83.3 ± 6.7

The experiments were performed in triplicate. Results are expressed as the mean ± standard deviation (SD); ns—no selectivity; na—no activity; nd—not determined.

**Table 3:** Anticoagulant and antiaggregating activity of compounds.

Cmpd	Latent Period, % of Control	Maximum Amplitude (MA), % of Control	Aggregation Rate, % of Control	Time to MA, % of Control	APTT $, % of Control
**3b**	**−2.4 (1.7–3.5)**	−3.8 (3.1–4.2) #	−11.4 (10.5–12.7) *	−18.5 (16.2–19.7) *,#	+5.6 (4.9–7.2) *,†
**3c**	−3.7 (3.1–4.2)	−2.4 (2.1–3.5) #	−13.7 (12.5–16.3) *,#	+8.4 (7.5–9.6) *,#	+7.7 (6.5–8.4) *,†
**3d**	**+4.8 (3.7–5.6) #**	−15.4 (13.3–16.7) *	−7.6 (7.1–10.5) *	+14.8 (13.5–16.7) *	**+4.7 (3.8–5.8)** †
**3e**	+6.5 (4.7–7.6) *,#	−12.1 (10.5–14.2) *	−31.7 (30.6–34.2) *,#	−18.2 (17.4–21.3) *,#	+4.9 (4.1–8.3) *,†
**3f**	+2.3 (1.7–3.5) #	−9.5 (8.2–10.9) *,#	−16.2 (15.7–17.1) *,#	−11.9 (10.2–13.6) *,#	+3.8 (2.4–4.3) †
**3g**	+20.3 (19.7–21.4) *,#	−20.1 (18.7–23.5) *,#	−18.4 (17.4–20.5) *,#	−21.4 (17.4–22.9) *,#	+8.3 (7.2–10.1) *,†
**3h**	+13.4 (11.7–15.2) *,#	−15.3 (14.8–18.2) *	−13.6 (12.4–14.9) *	−22.5 (21.7–23.9) *,#	+8.7 (6.8–10.2) *,†
**3i**	+5.1 (4.7–5.4) #	−5.3 (4.7–7.5) *,#	+2.7 (2.5–3.9) #	−16.4 (16.1–19.5) *,#	+5.7 (4.1–7.6) *,†
**3j**	+3.1 (2.9–4.2) #	−4.2 (2.6–4.8) #	−10.4 (9.3–12.7) *	+12.6 (10.3–15.7) *	+7.3 (5.9–8.2) †
**3k**	+7.3 (6.2–8.5) *,#	−11.5 (9.2–13.1) *	−27.4 (26.3–29.5) *,#	−5.1 (3.7–6.4) *,#	+9.7 (8.8–10.5) *,†
**4a**	+25.7 (24.8–27.5) *,#	−14.2 (13.1–15.7) *	−12.4 (10.2–14.3) *	+12.3 (11.7–13.5) *	**+6.1 (4.5–7.9)** †
**4b**	−3.1 (2.9–4.1)	−2.4 (1.7–2.6) #	−4.1 (3.9–5.6) #	−9.7 (8.1–11.4) *,#	+7.4 (6.3–9.2) *,†
**4c**	+5.2 (4.7–6.1) *,#	−6.8 (5.7–7.3) *,#	−8.2 (7.1–10.4) *	+11.8 (10.4–13.7) *	+2.5 (1.7–3.2) †
**4d**	+4.3 (3.8–5.7) #	−6.7 (5.4–8.3) *,#	−7.9 (7.1–10.4) *,#	+10.2 (9.1–13.4) *	+2.7 (1.7–2.8) †
**4e**	+2.6 (2.1–3.8) #	−7.2 (5.4–9.2) *,#	+3.1 (2.9–4.2) #	−11.4 (9.4–12.3) *,#	+2.4 (1.8–3.7) †
**Acetylsalicylic acid**	−2.1 (1.1–2.6)	−13.7 (10.8–16.4) *	−10.5 (7.6–12.3) *	+10.5 (8.7–13.4) *	-
**Heparin sodium**	-	-	-	-	+20.3 (19.7–21.4) *

* *p* < 0.05—compared to control; # *p* < 0.05—compared to acetylsalicylic acid; † *p* < 0.05—compared to heparin sodium; $ APTT—activated partial thromboplastin time.

## Data Availability

Not applicable.

## Supplementary Materials

The following supporting information can be downloaded at: https://www.mdpi.com/article/10.3390/molecules29194764/s1, Figures S1–S48—copies of NMR spectra of all synthesized compounds.
