# Supplementary material for: Base-Catalyzed Reaction of Isatins and (3-Hydroxyprop-1-yn-1-yl)phosphonates as a Tool for the Synthesis of Spiro-1,3-dioxolane Oxindoles with Anticancer and Anti-Platelet Properties
Source: Molecules. 2024 Oct 8;29(19):4764. doi: 10.3390/molecules29194764 (PMC11477635; doi:10.3390/molecules29194764)

Supporting Information

For

Base-catalyzed reaction of isatins and (3-hydroxyprop-1-yn-1-yl)phosphonates as a tool for the synthesis of spiro-1,3-dioxolane oxindoles with anticancer and anti-platelet properties

Arina V. Murashkina [1], Andrei V. Bogdanov [2,*], Alexandra D. Voloshina [3], Anna P. Lyubina [3], Alexandr V. Samorodov [4], Alexander Y. Mitrofanov [1,*], Irina P. Beletskaya [1], Elena A. Smolyarchuk [5], Kseniya A. Zavadich [5], Zulfiya A. Valiullina [4], Kseniya A. Nazmieva [4], Vladislav I. Korunas [4] and Irina D. Krylova [4]

[1] Department of Chemistry, M. V. Lomonosov Moscow State University, 119991 Moscow, Russia; arinamr@mail.ru (A.V.M.); beletska@org.chem.msu.ru (I.P.B.)
[2] A. M. Butlerov Institute of Chemistry, Kazan Federal University, 420008 Kazan, Russia
[3] Arbuzov Institute of Organic and Physical Chemistry, FRC Kazan Scientific Center, Russian Academy of Sciences, 420088 Kazan, Russia; microbi@iopc.ru (A.D.V.); aplyubina@gmail.com (A.P.L.)
[4] Department of Pharmacology, Bashkir State Medical University, 450008 Ufa, Russia; avsamorodov@gmail.com (A.V.S.); z_suleimanova@mail.ru (Z.A.V.); nazmievaksenia@gmail.com (K.A.N.); bsmu.korunas@gmail.com (V.I.K.); i.krylova16@yandex.ru (I.D.K.)
[5] The A.P. Nelyubin Institute of Pharmacy, Sechenov First Moscow State Medical University (Sechenov University), 119571 Moscow, Russia; smolyarchuk_e_a@staff.sechenov.ru (E.A.S.); zavadich_k_a@staff.sechenov.ru (K.A.Z.)
* Correspondence: abogdanov@inbox.ru; Tel.: +7-843-272-7384 (A.B.); mitrofanov@org.chem.msu.ru

NMR spectra

Figure S1. ^{1}H NMR spectrum of compound (**3a**)

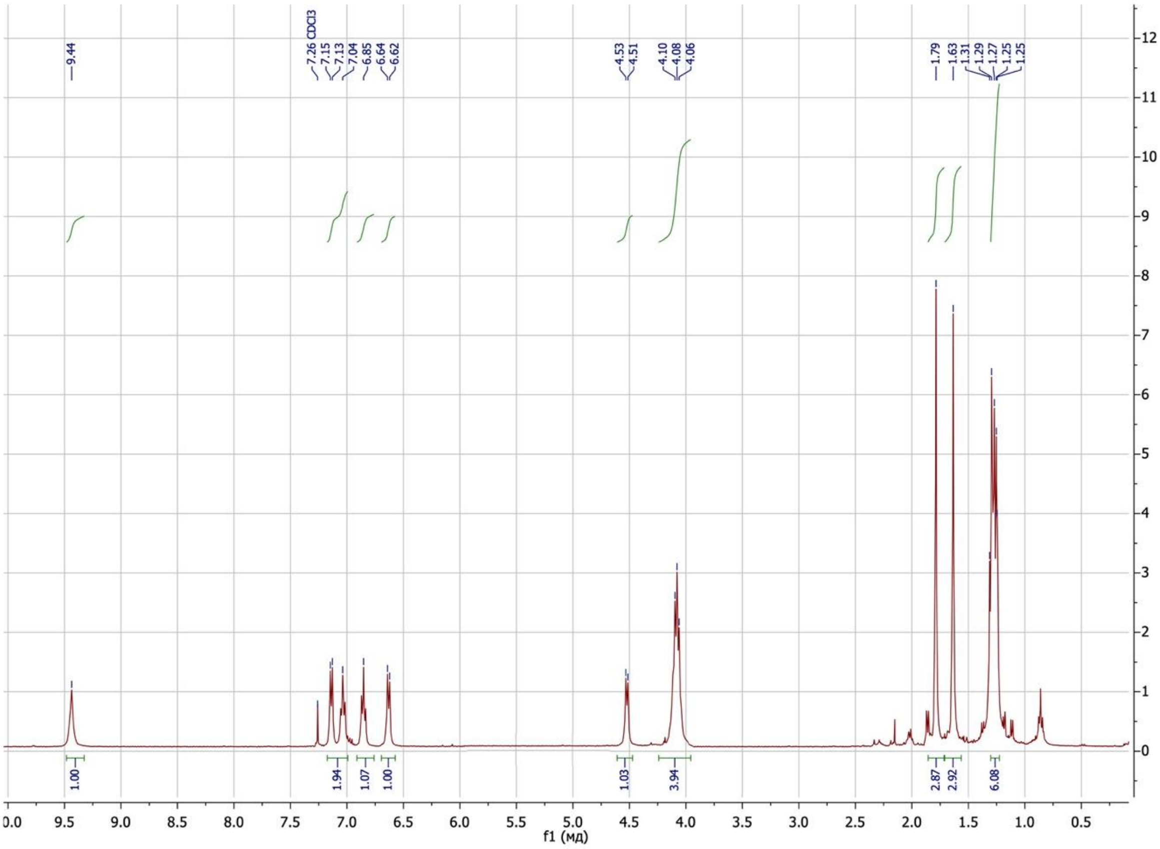

Figure S2. ^{13}C NMR spectrum of compound (**3a**)

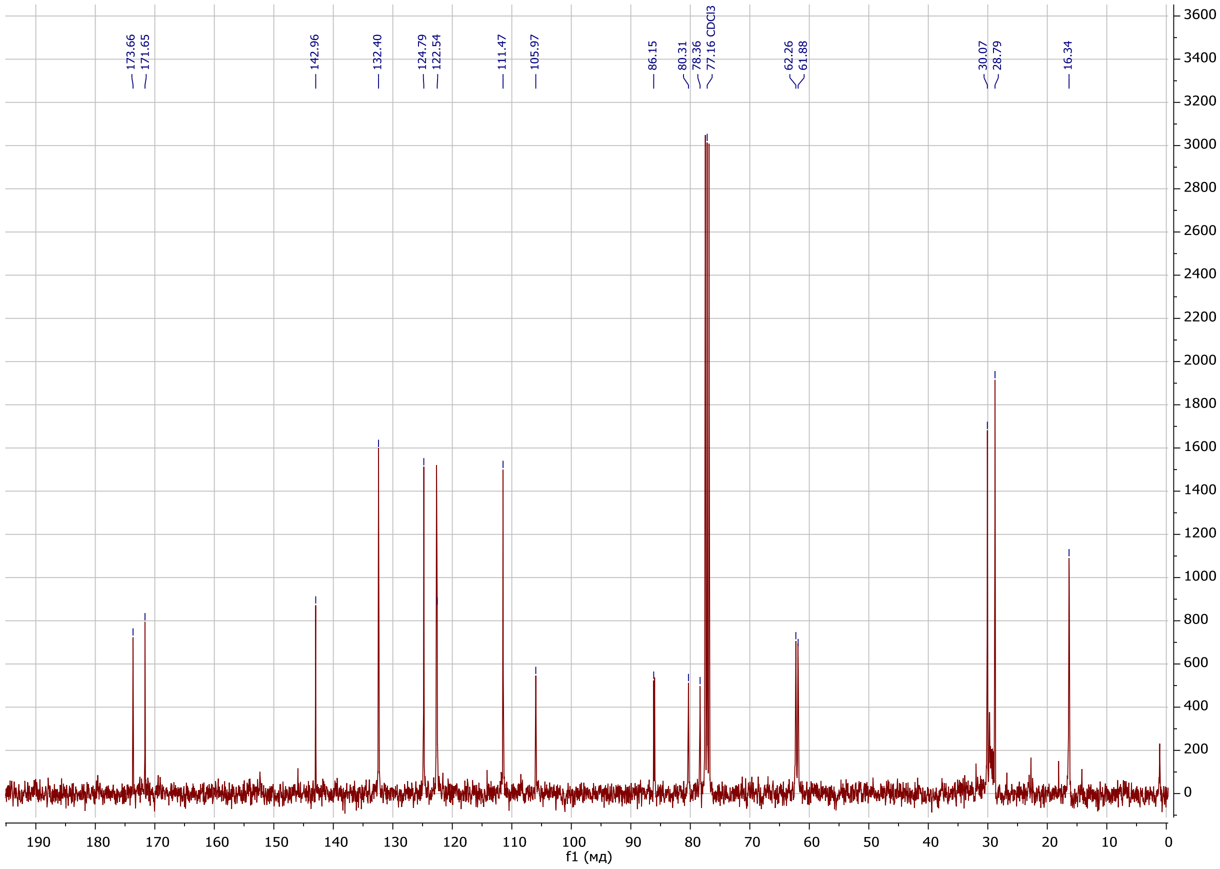

Figure S3. ^{31}P NMR spectrum of compound (**3a**)

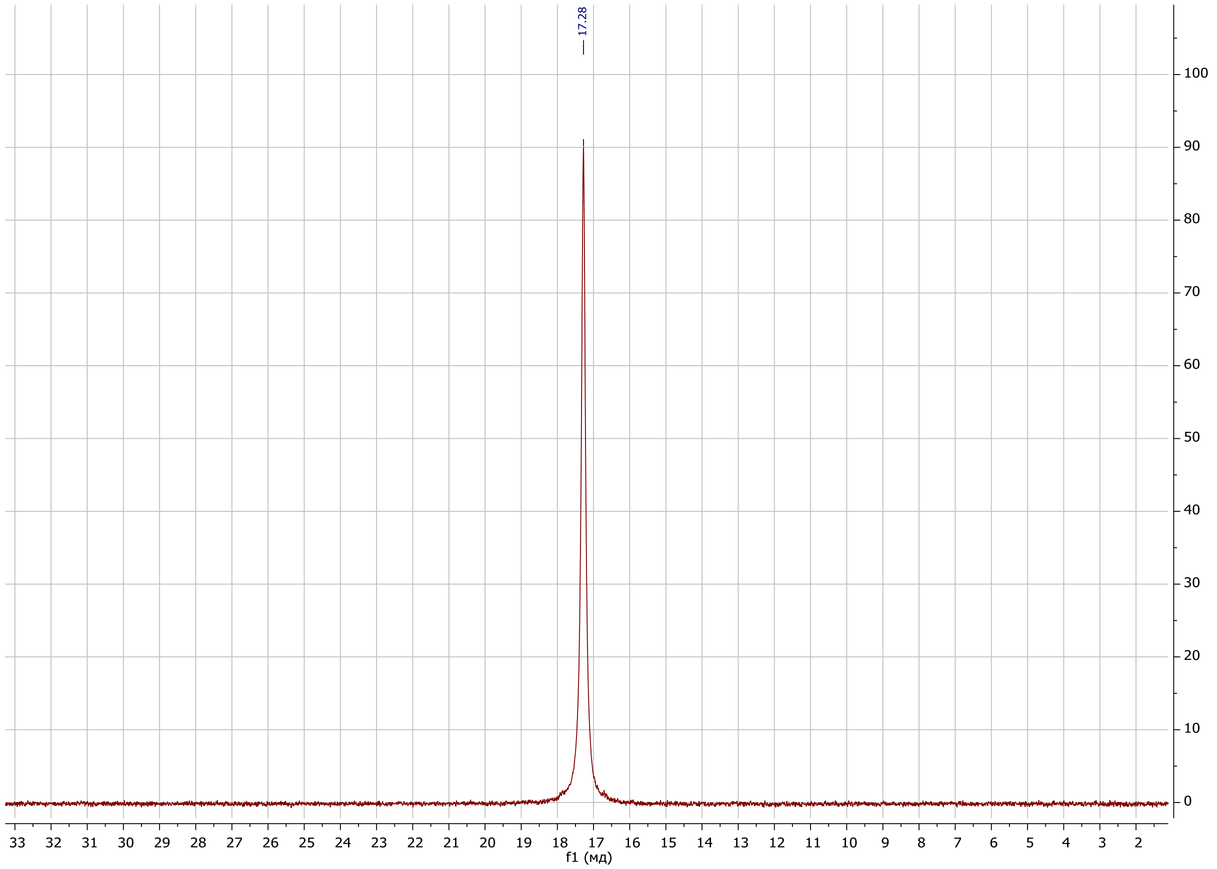

Figure S4. ^{1}H NMR spectrum of compound (**3b**)

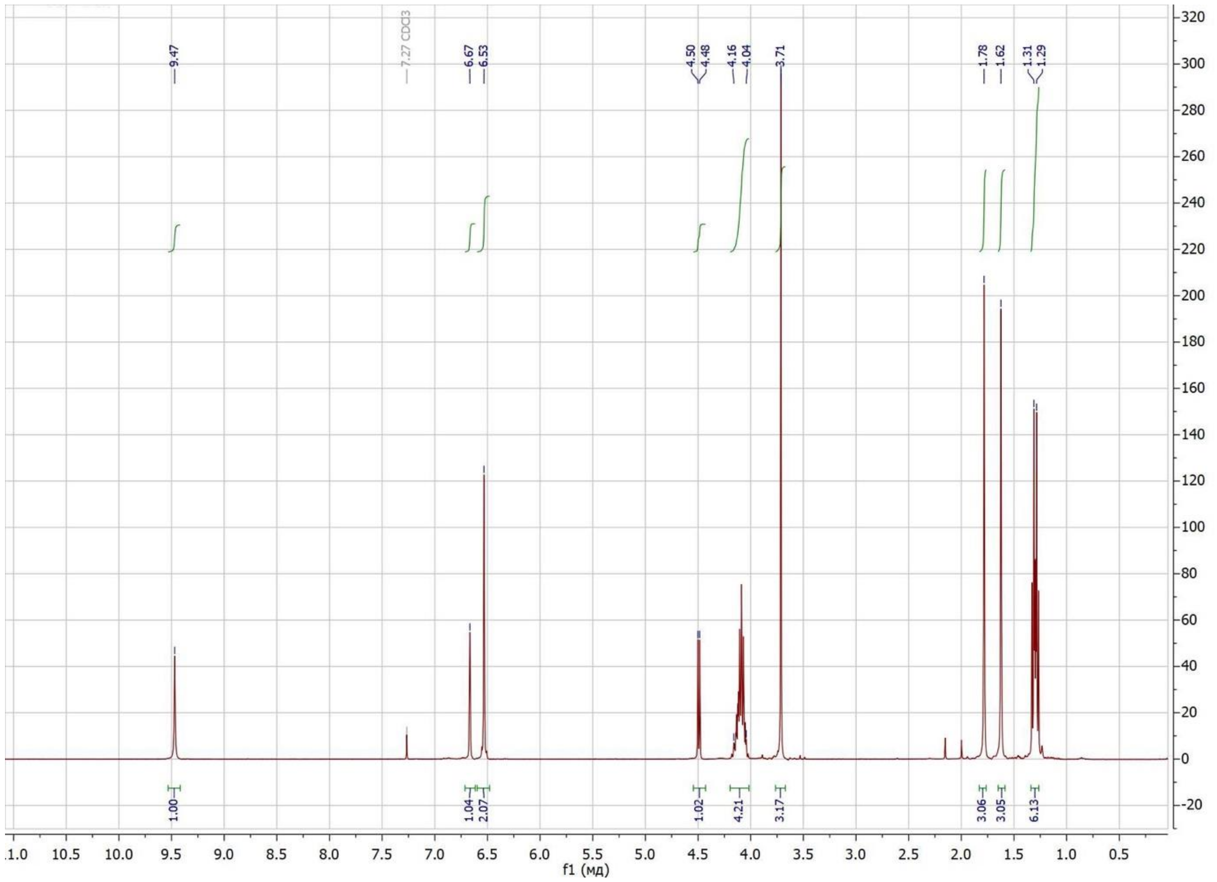

Figure S5. ^{13}C NMR spectrum of compound (**3b**)

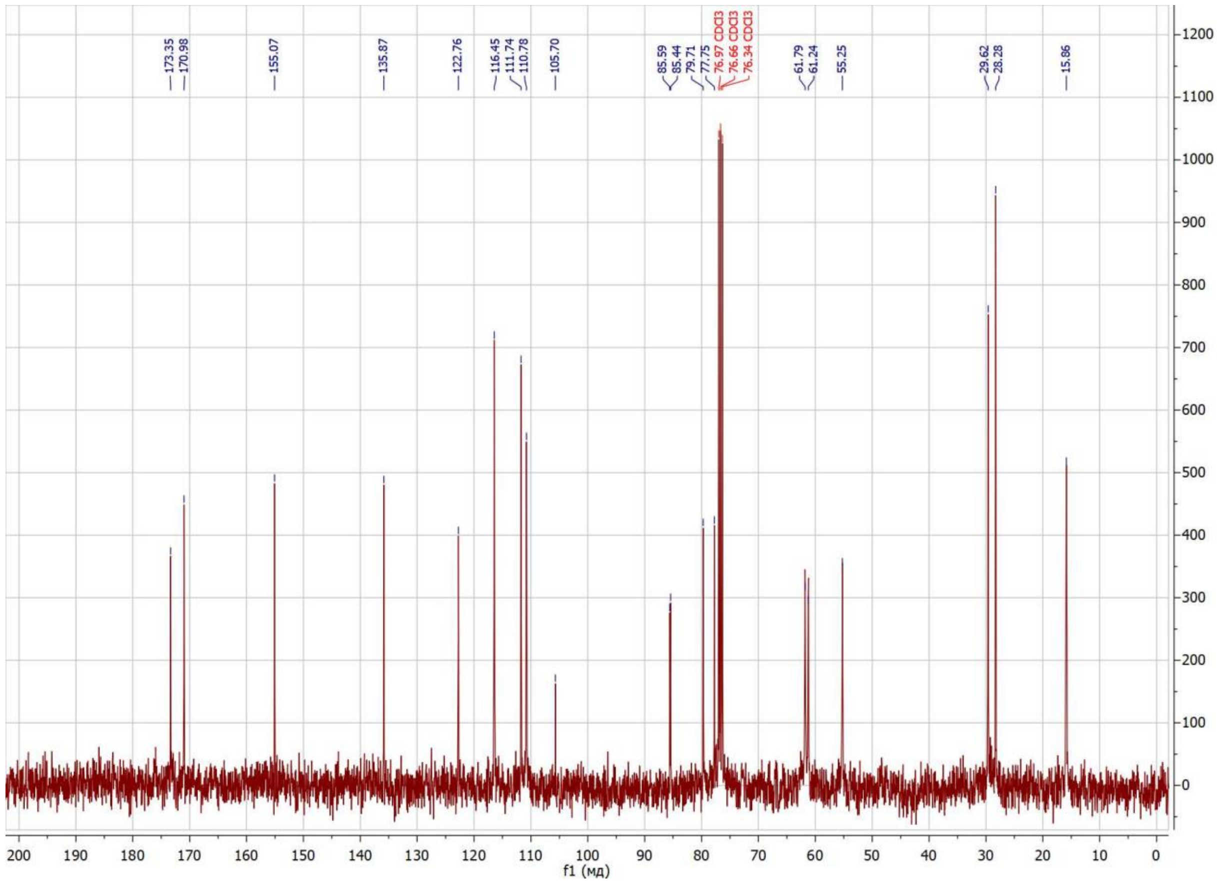

Figure S6. ^{31}P NMR spectrum of compound (**3b**)

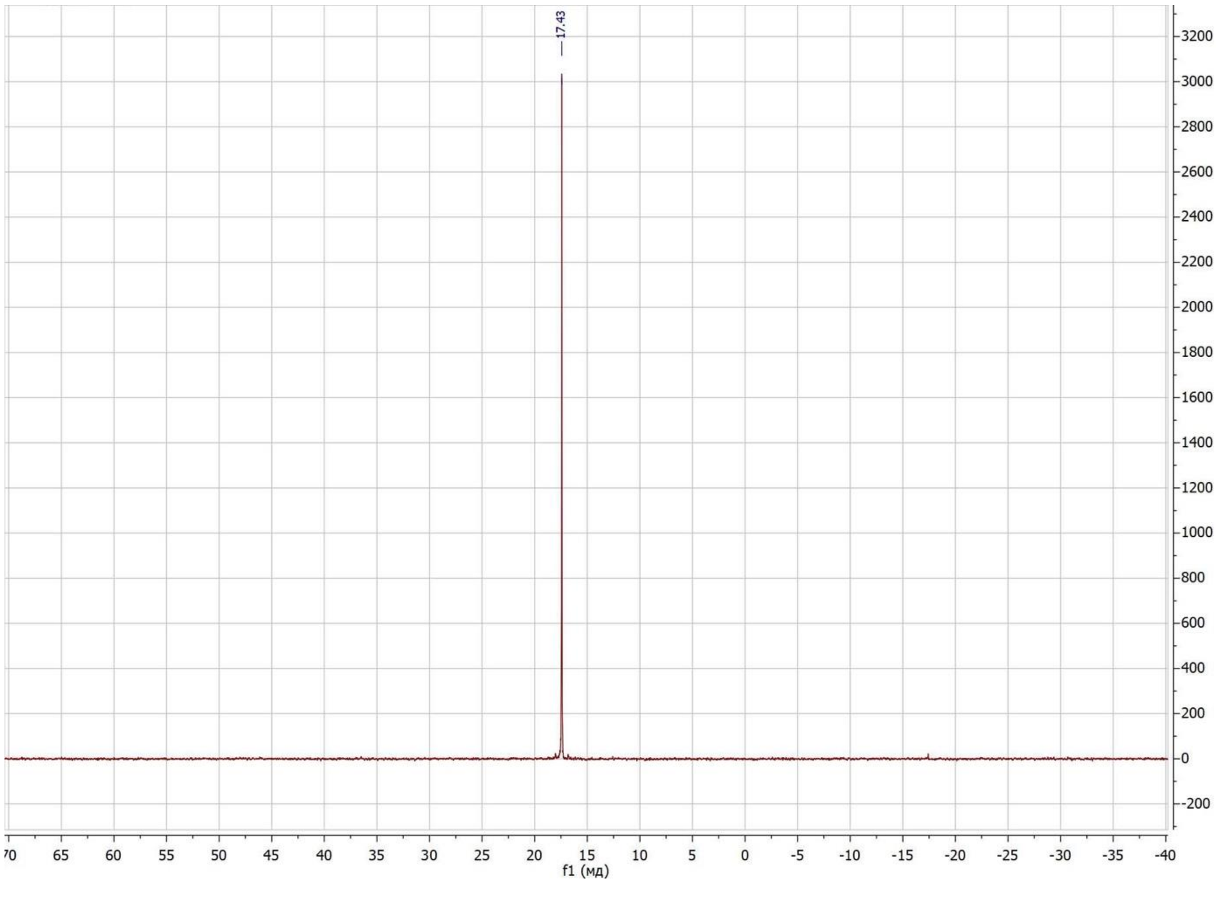

Figure S7. ^{1}H NMR spectrum of compound (**3c**)

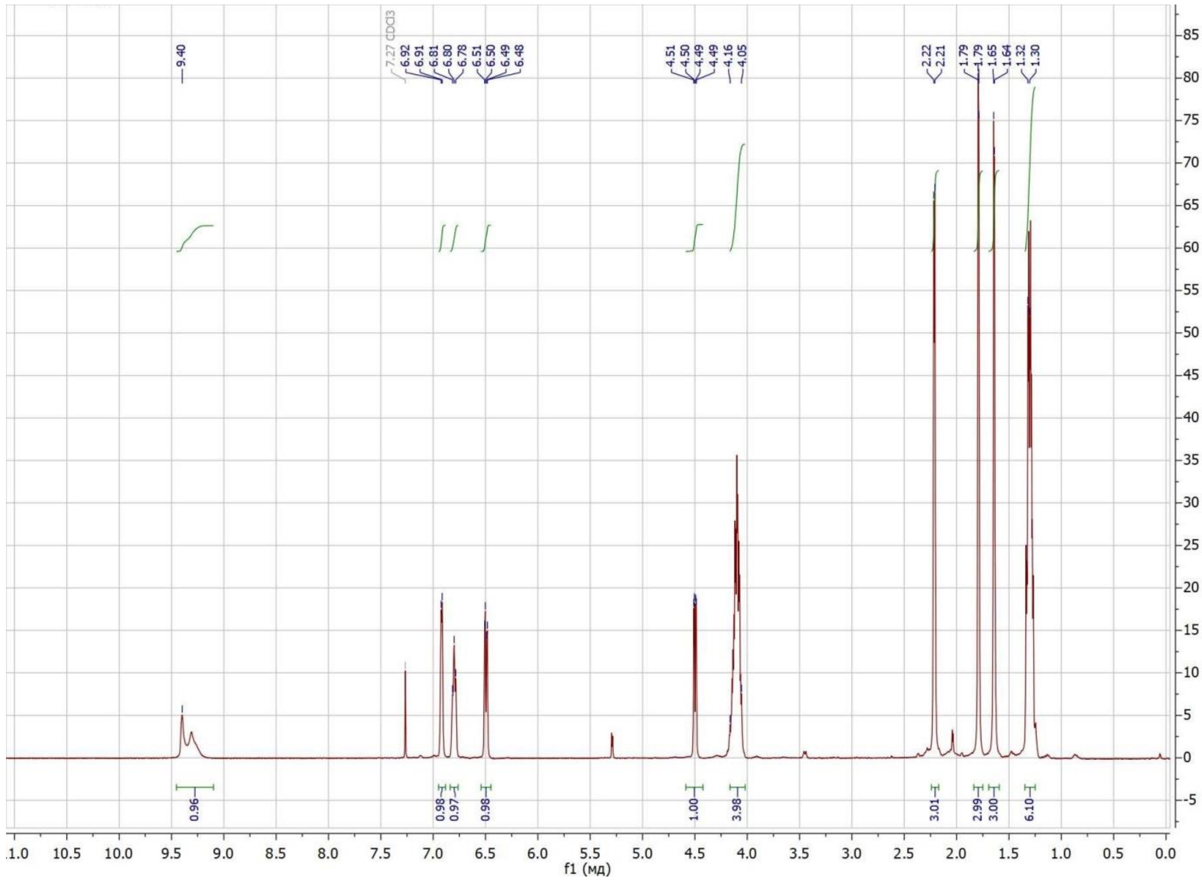

Figure S8. ^{13}C NMR spectrum of compound (**3c**)

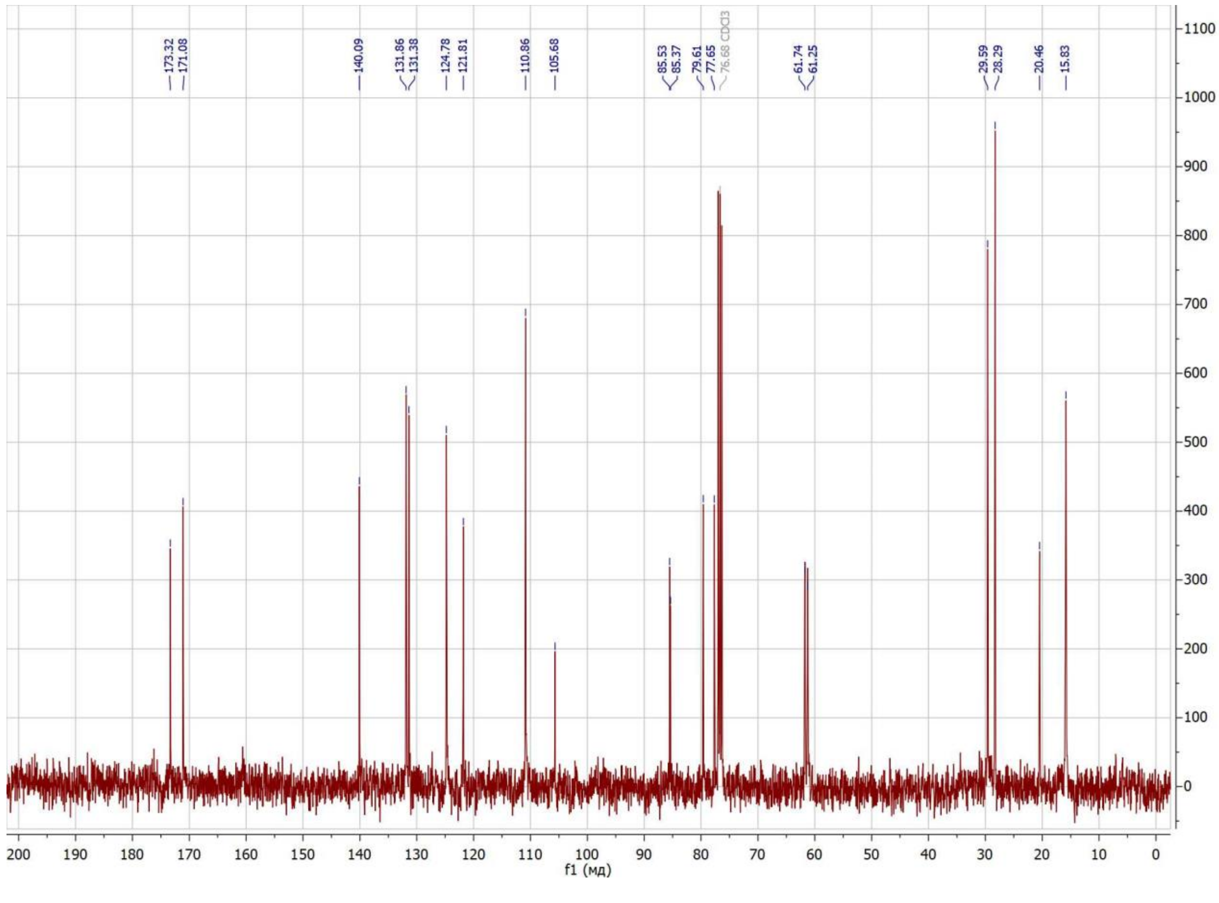

Figure S9. ^{31}P NMR spectrum of compound (**3c**)

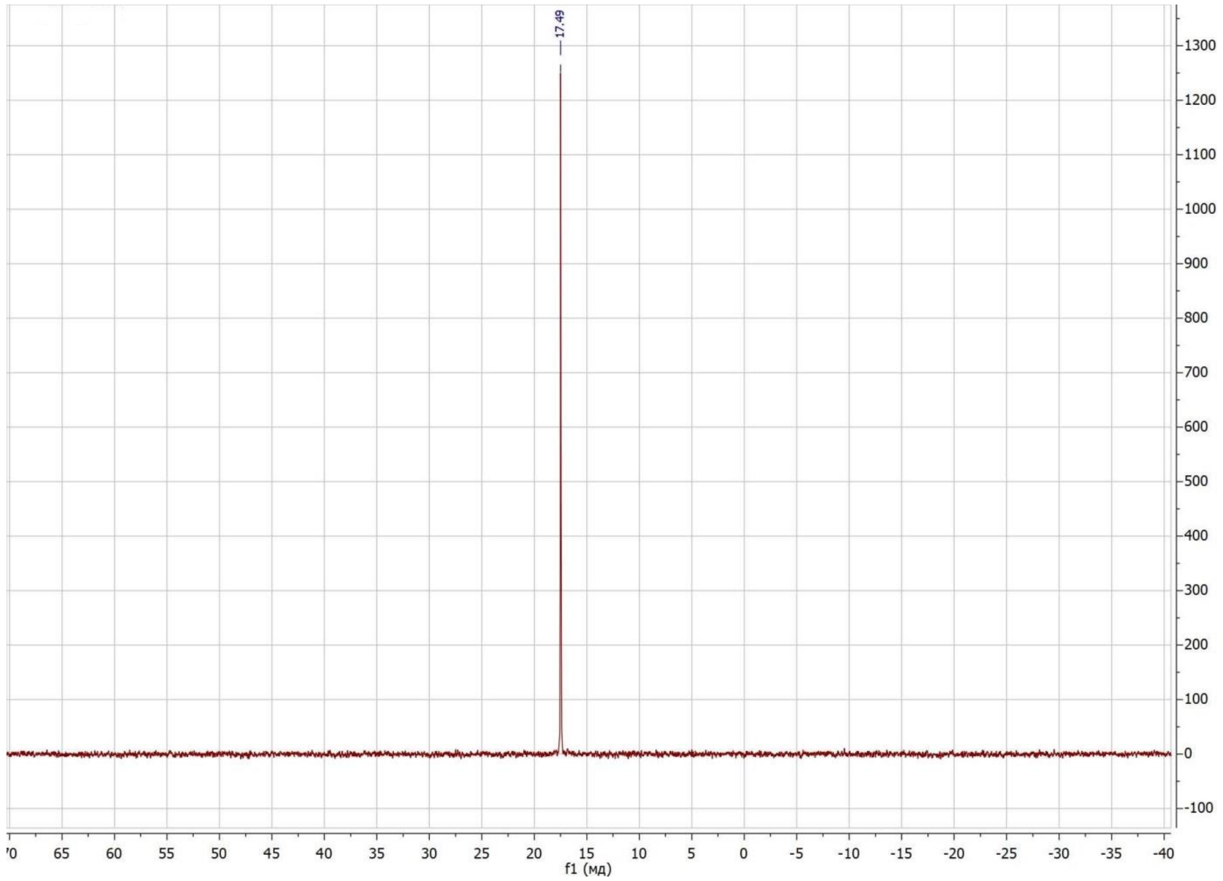

Figure S10. ^{1}H NMR spectrum of compound (**3d**)

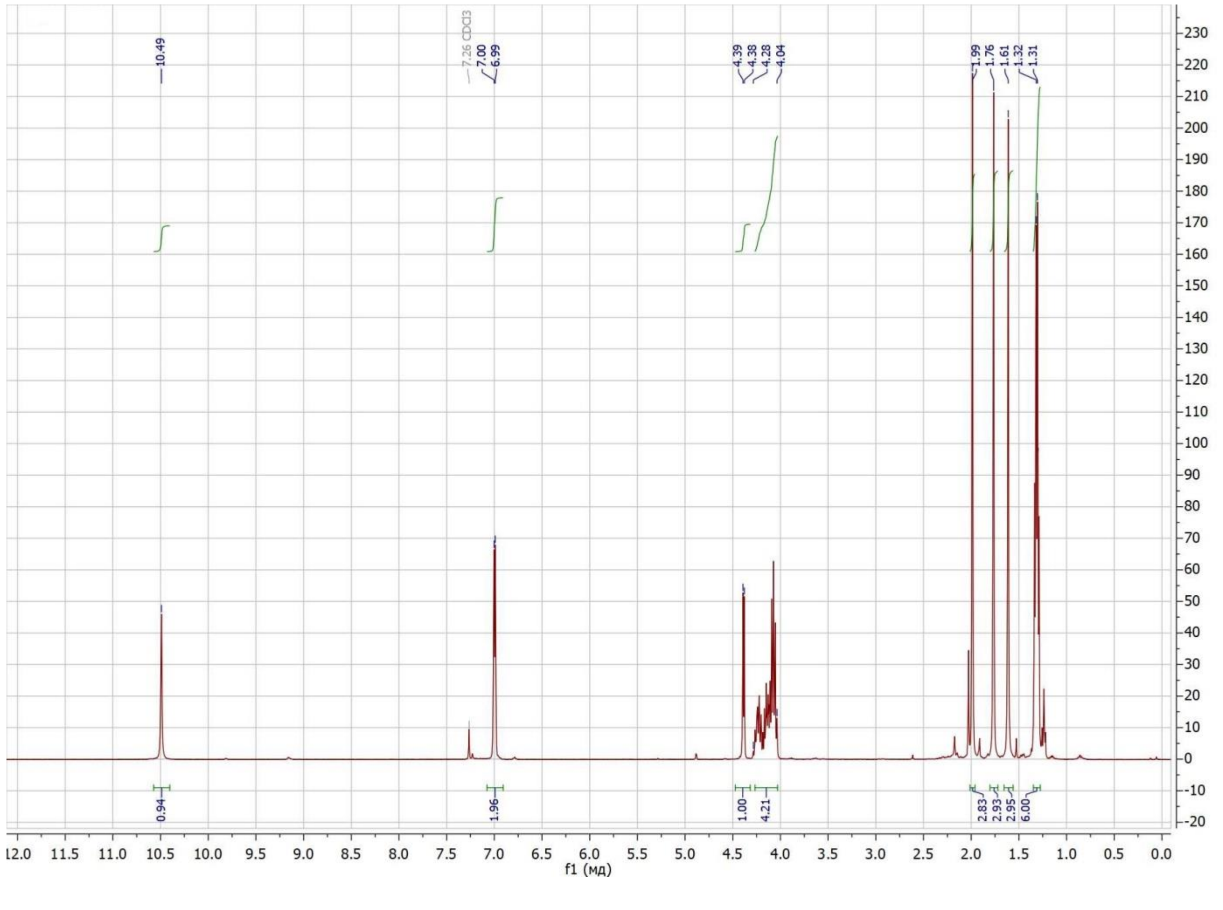

Figure S11. ^{13}C NMR spectrum of compound (**3d**)

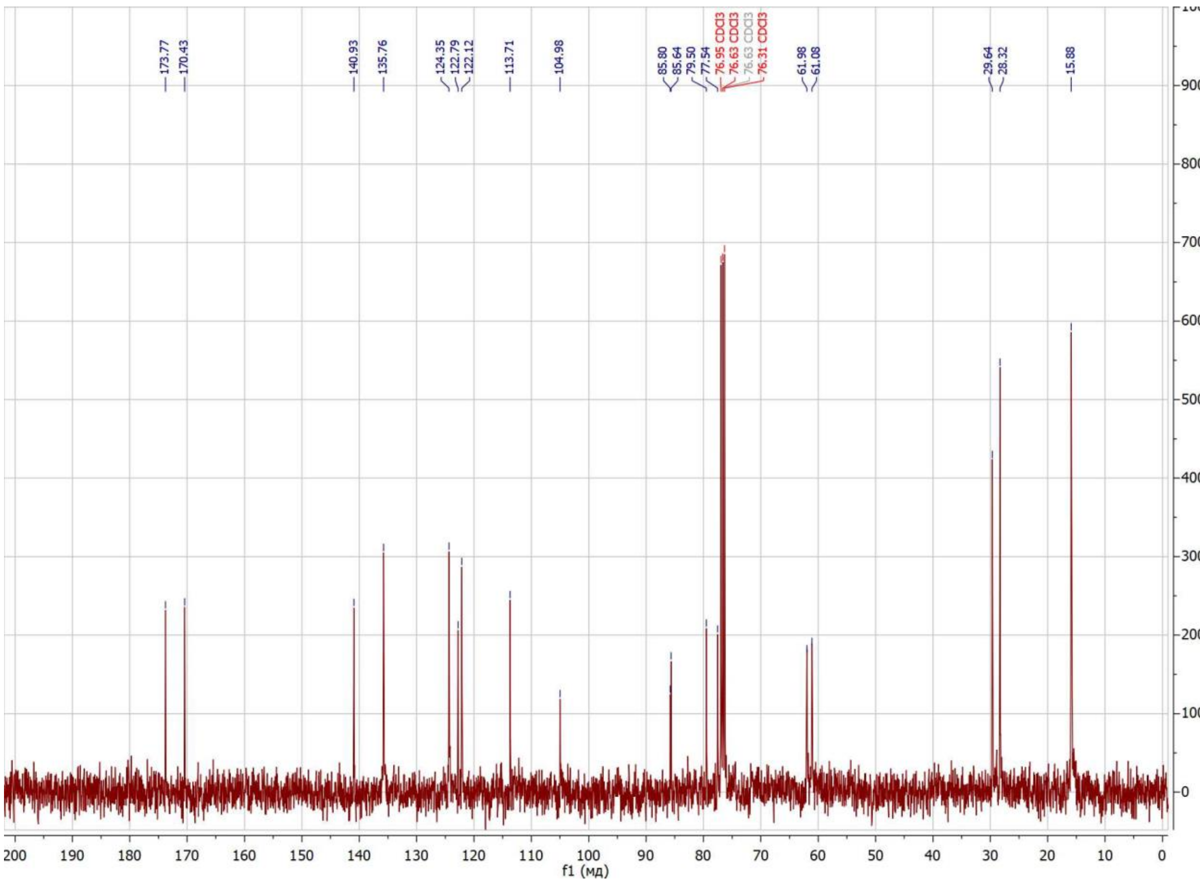

Figure S12. ^{31}P NMR spectrum of compound (**3d**)

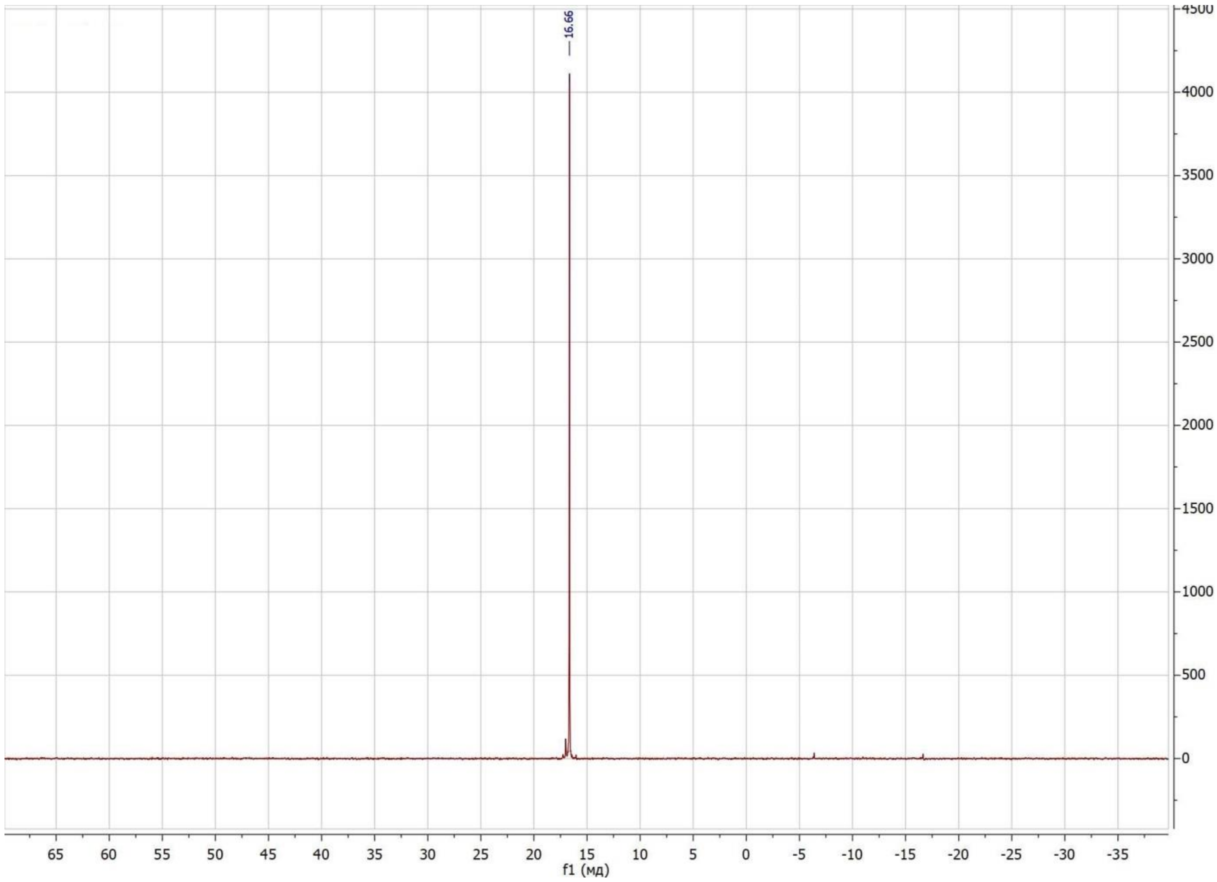

Figure S13. ^{1}H NMR spectrum of compound (**3e**)

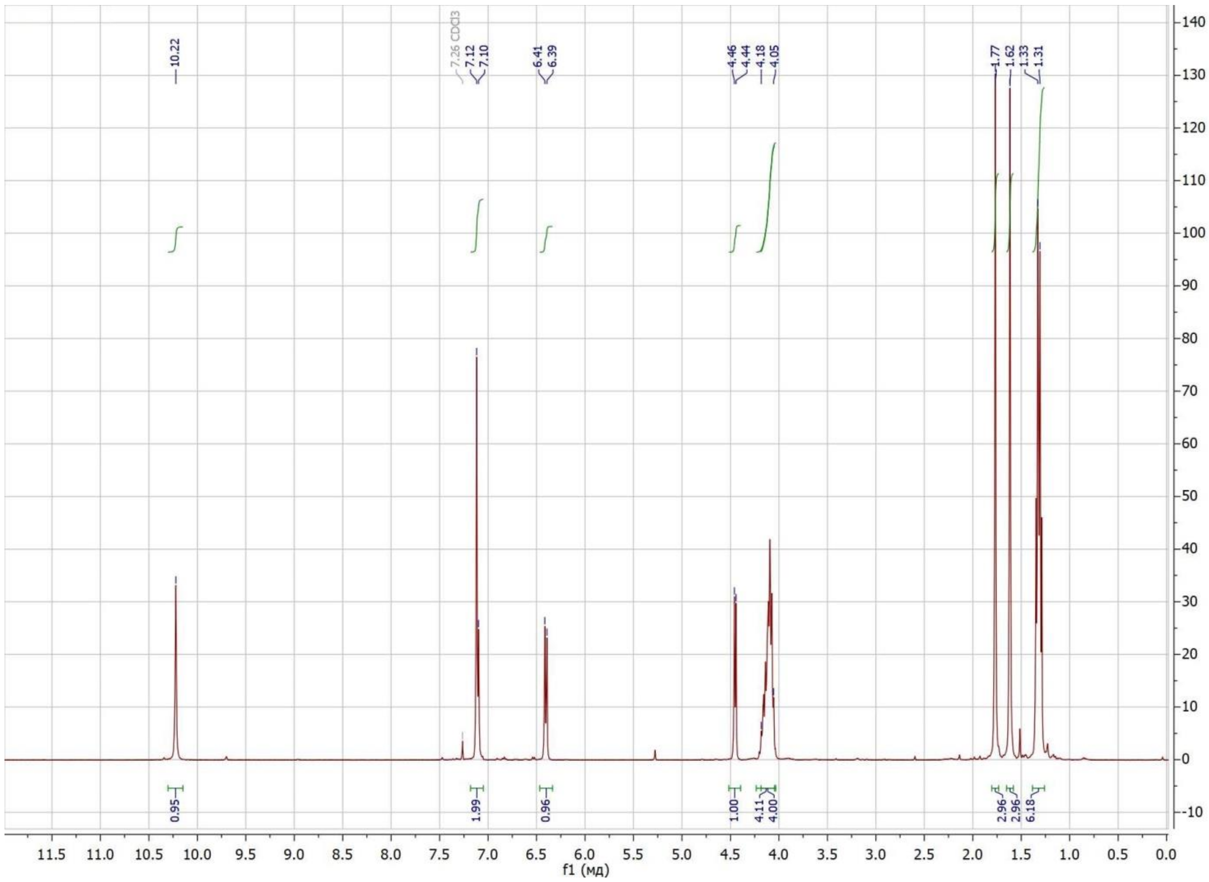

Figure S14. ^{13}C NMR spectrum of compound (**3e**)

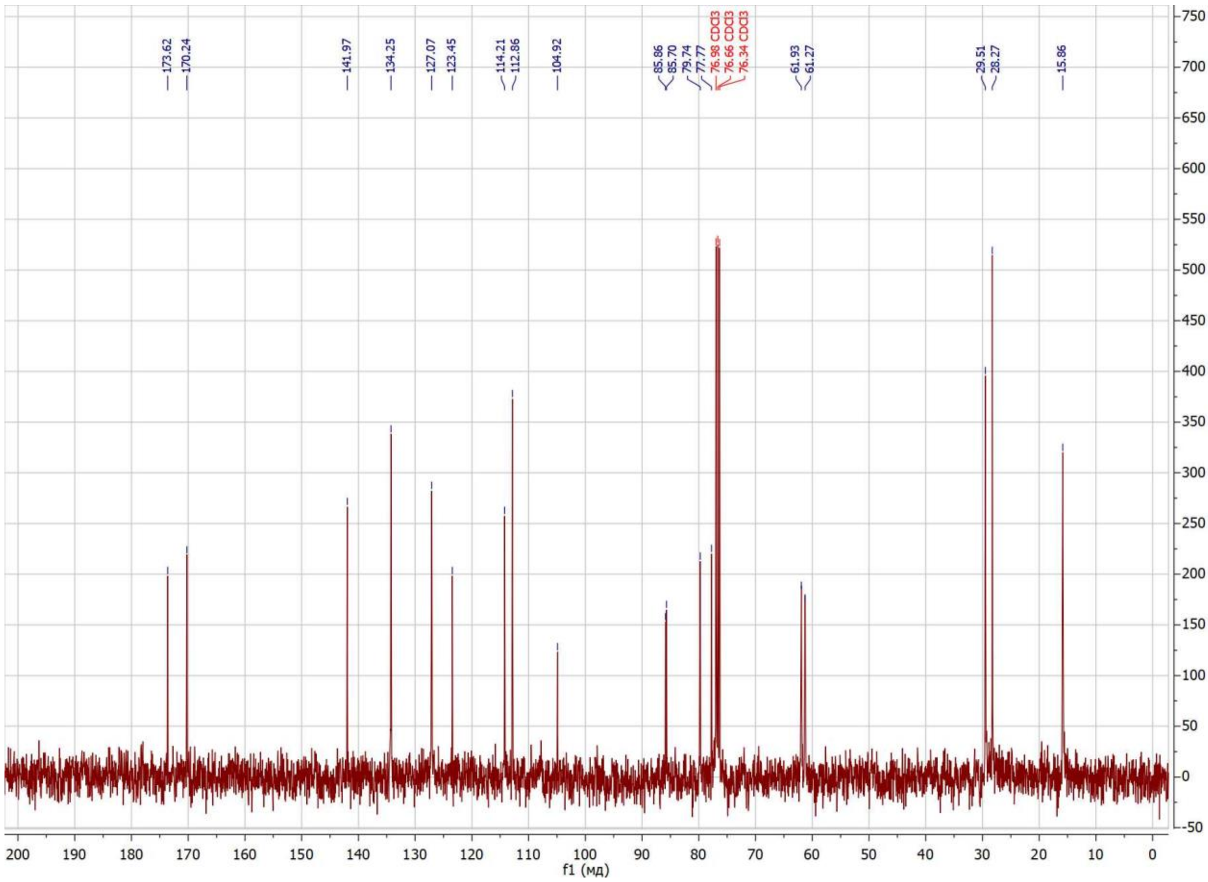

Figure S15. ^{31}P NMR spectrum of compound (**3e**)

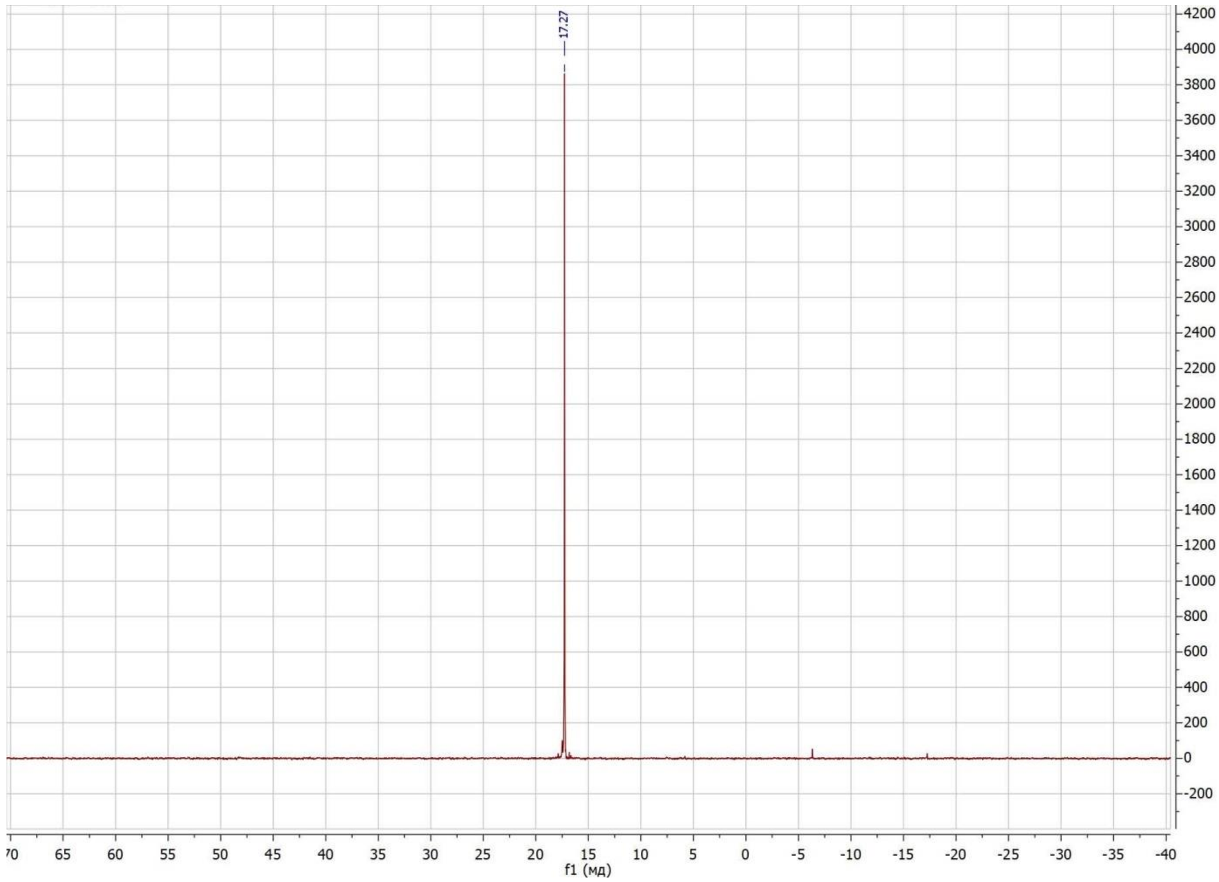

Figure S16. ^{1}H NMR spectrum of compound (**3f**)

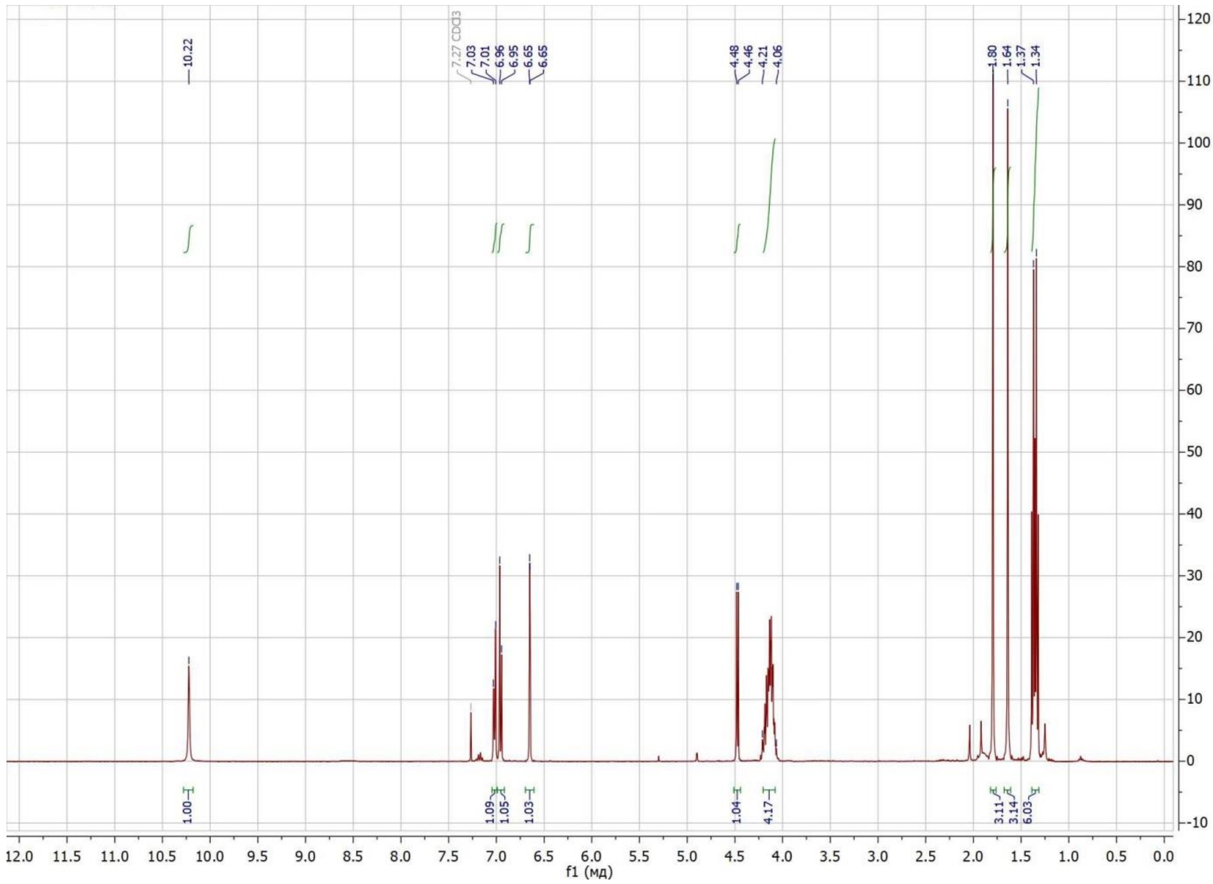

Figure S17. ^{13}C NMR spectrum of compound (**3f**)

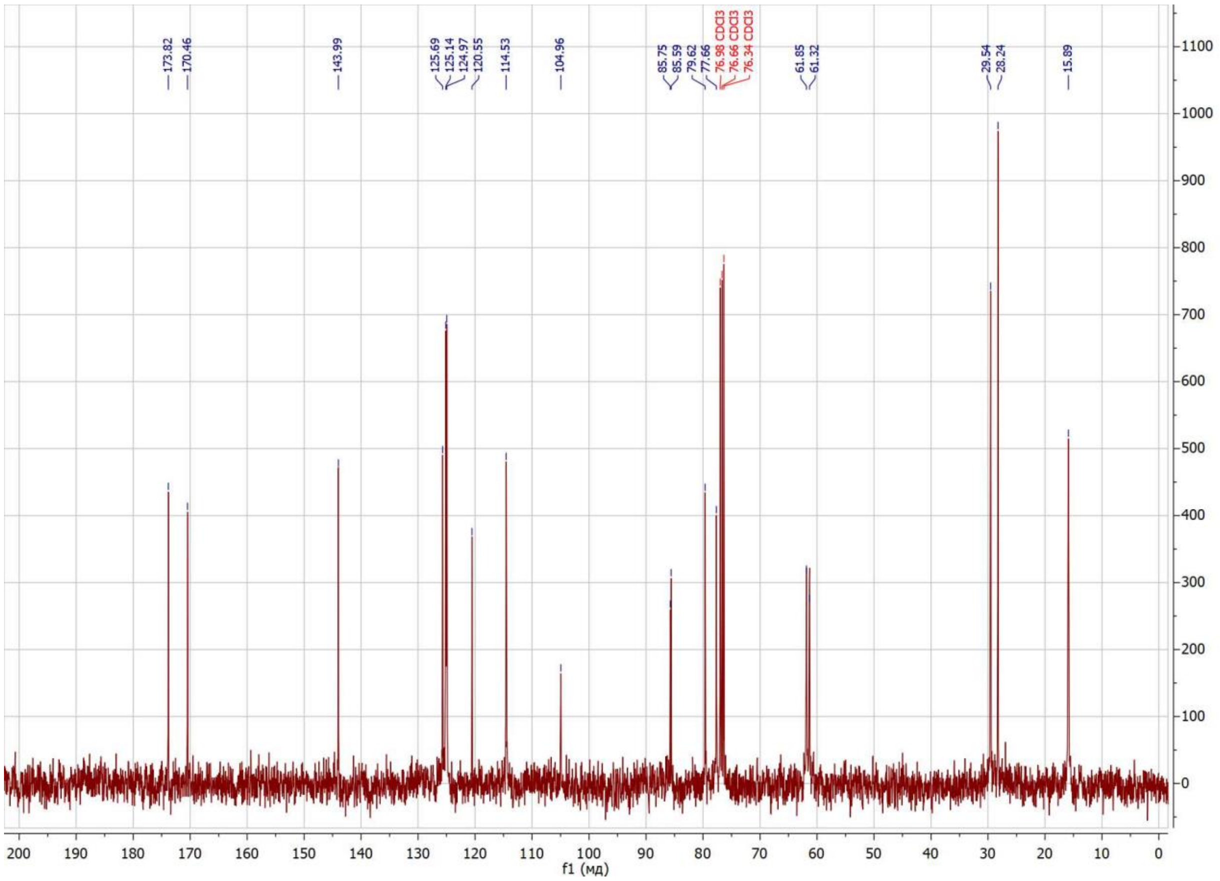

Figure S18. ^{31}P NMR spectrum of compound (**3f**)

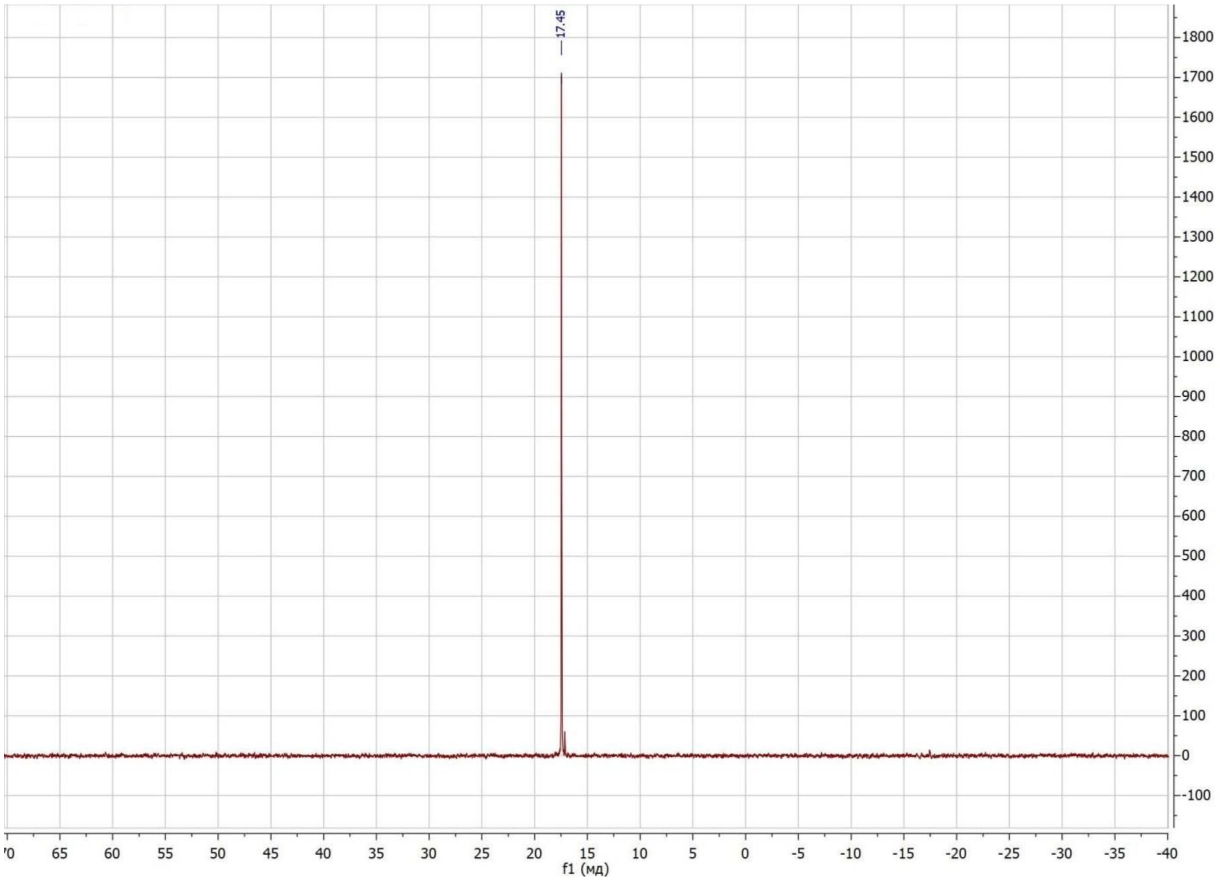

Figure S19. ^{1}H NMR spectrum of compound (**3g**)

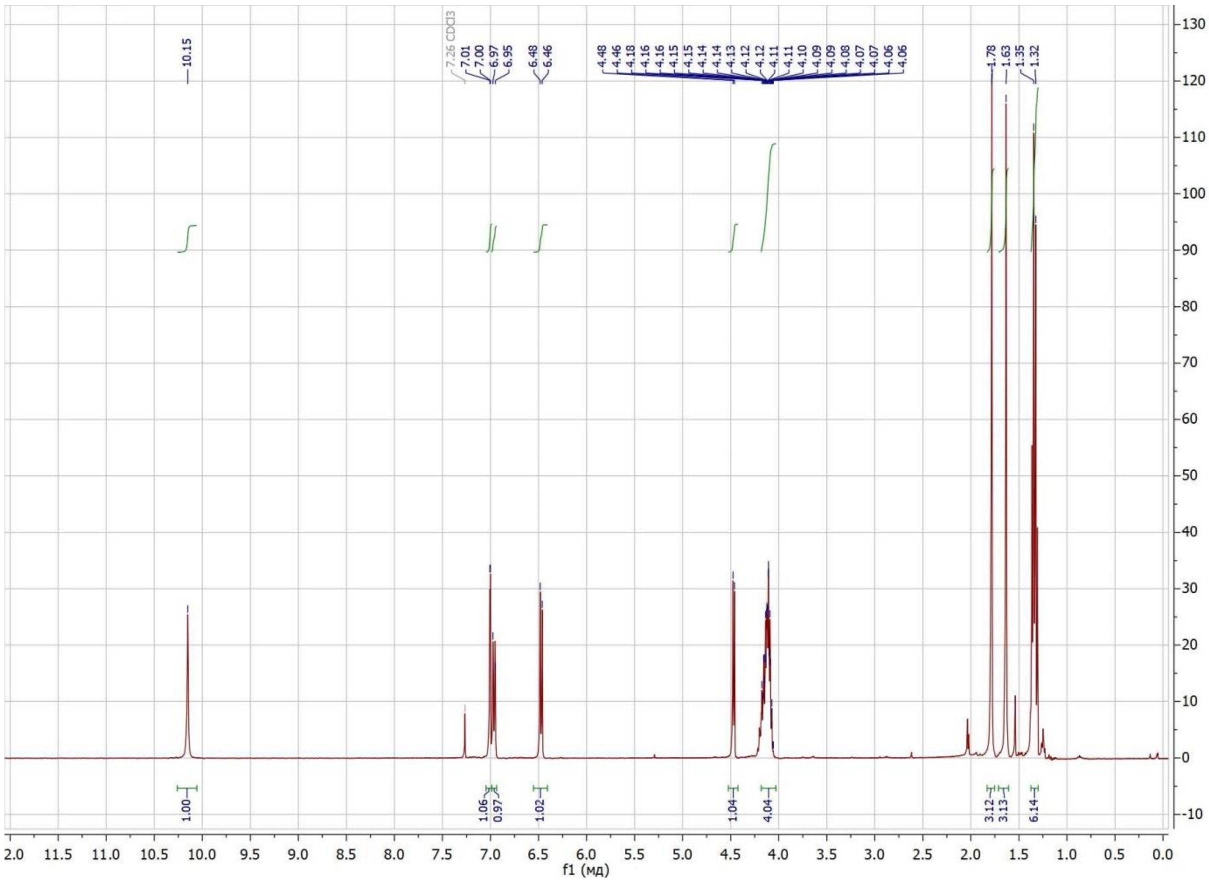

Figure S20. ^{13}C NMR spectrum of compound (**3g**)

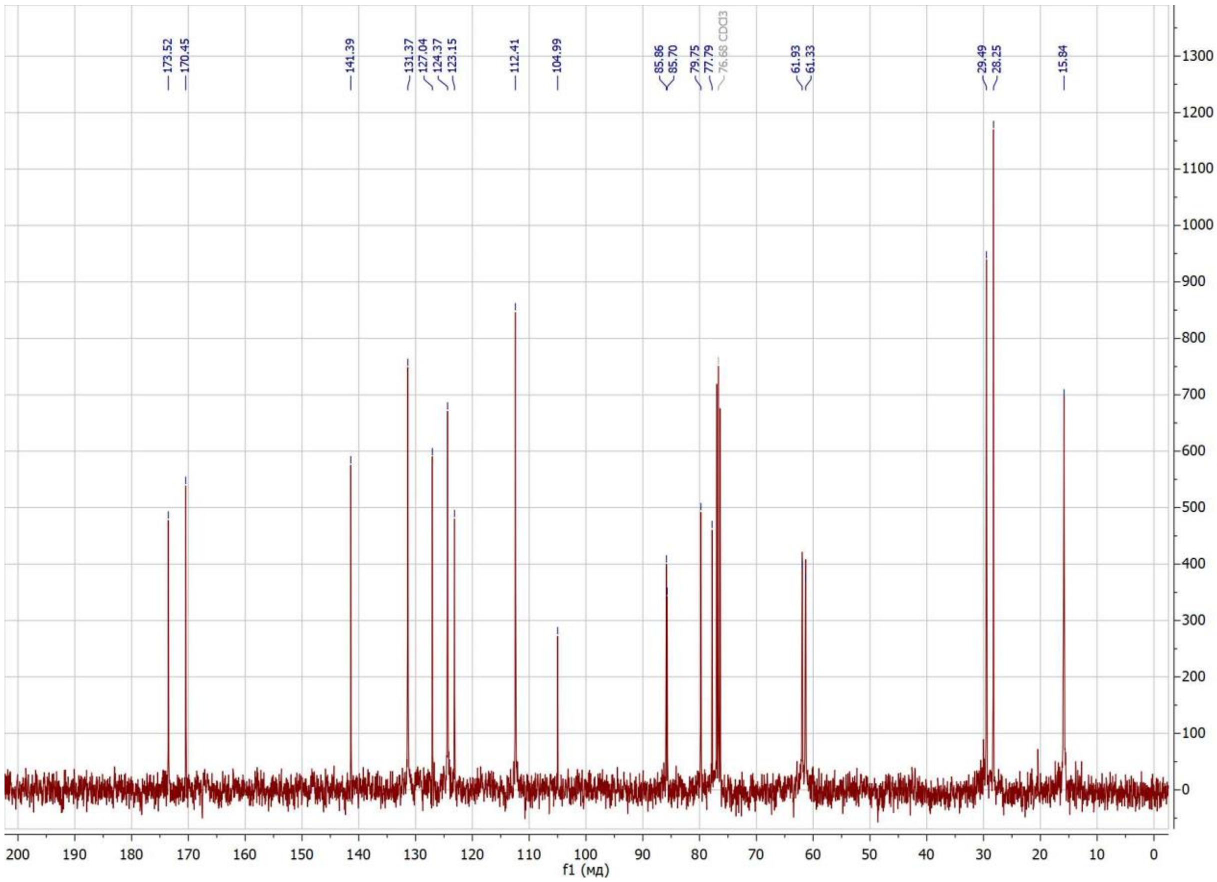

Figure S21. ^{31}P NMR spectrum of compound (**3g**)

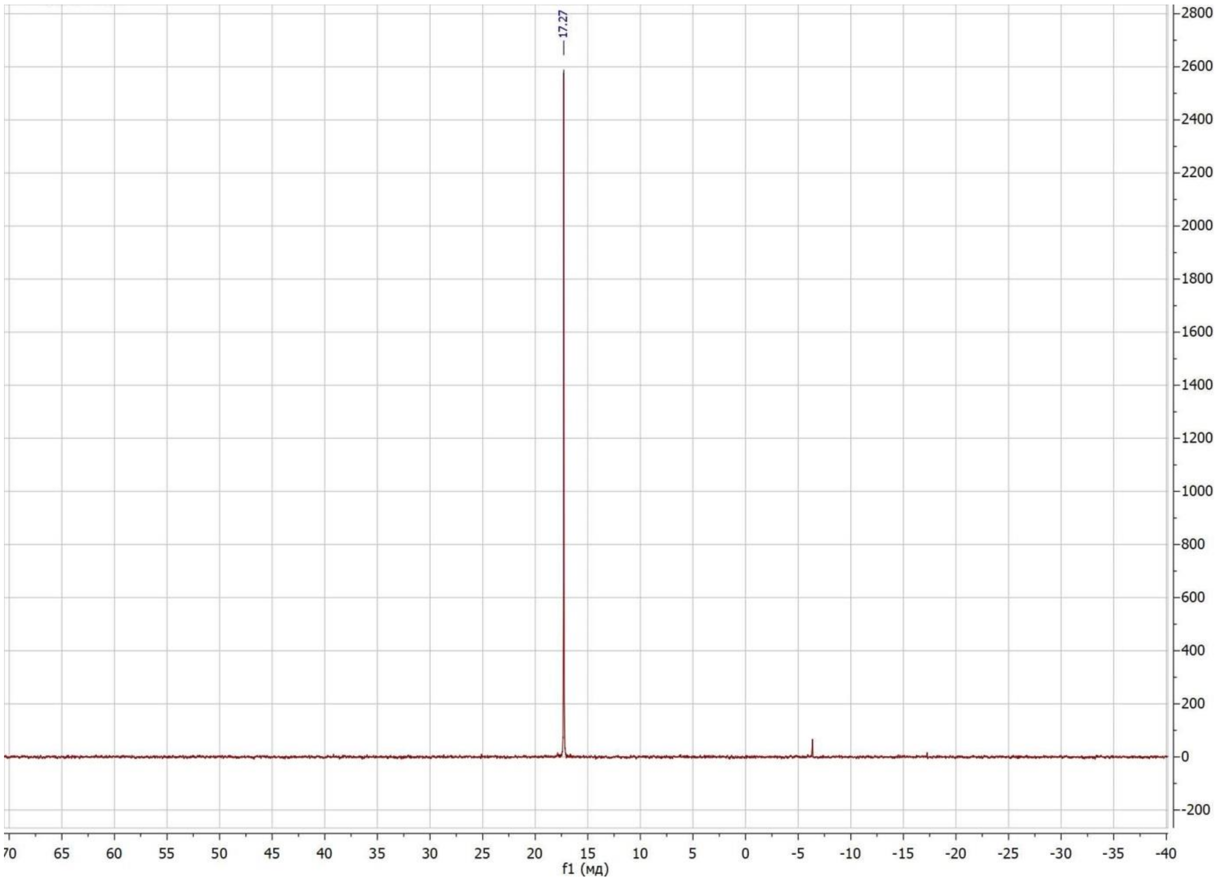

Figure S22. ^{1}H NMR spectrum of compound (**3h**)

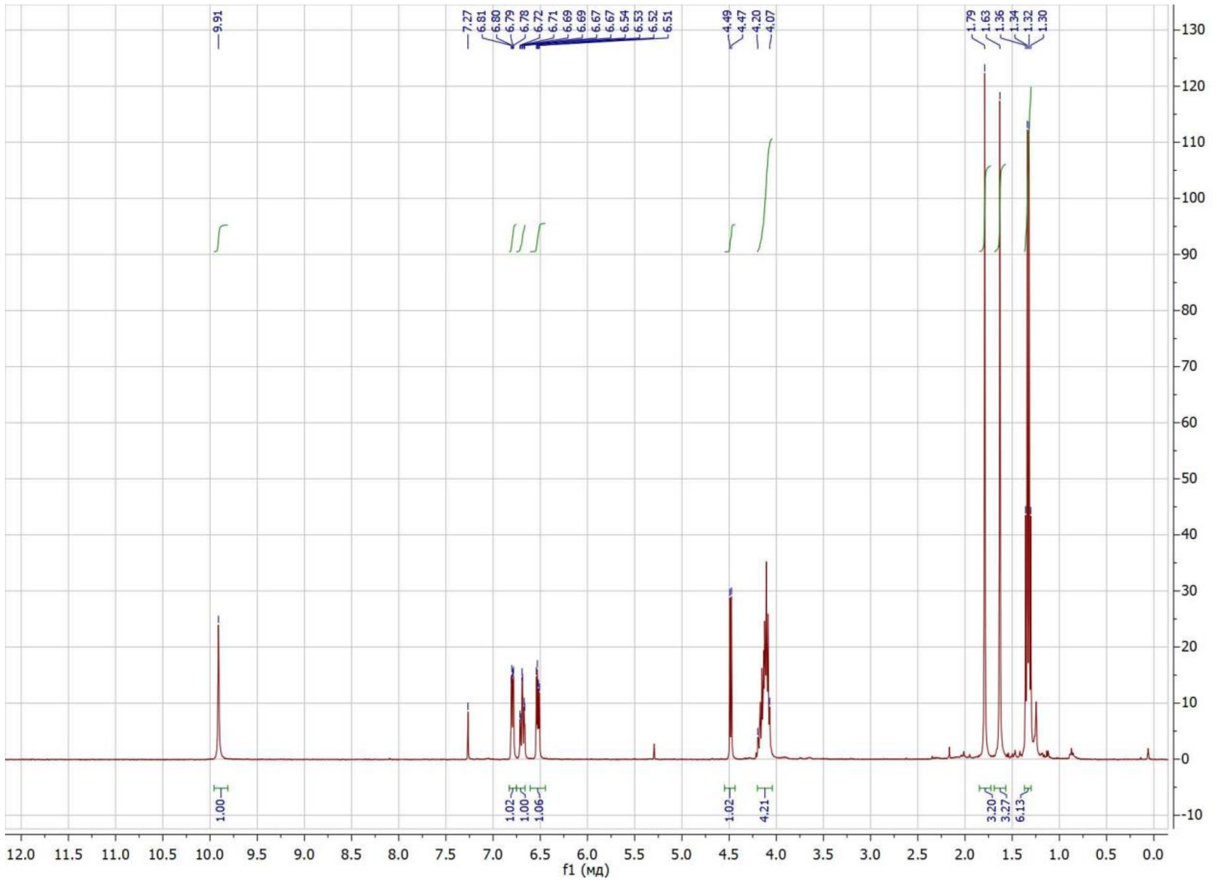

Figure S23. ^{13}C NMR spectrum of compound (**3h**)

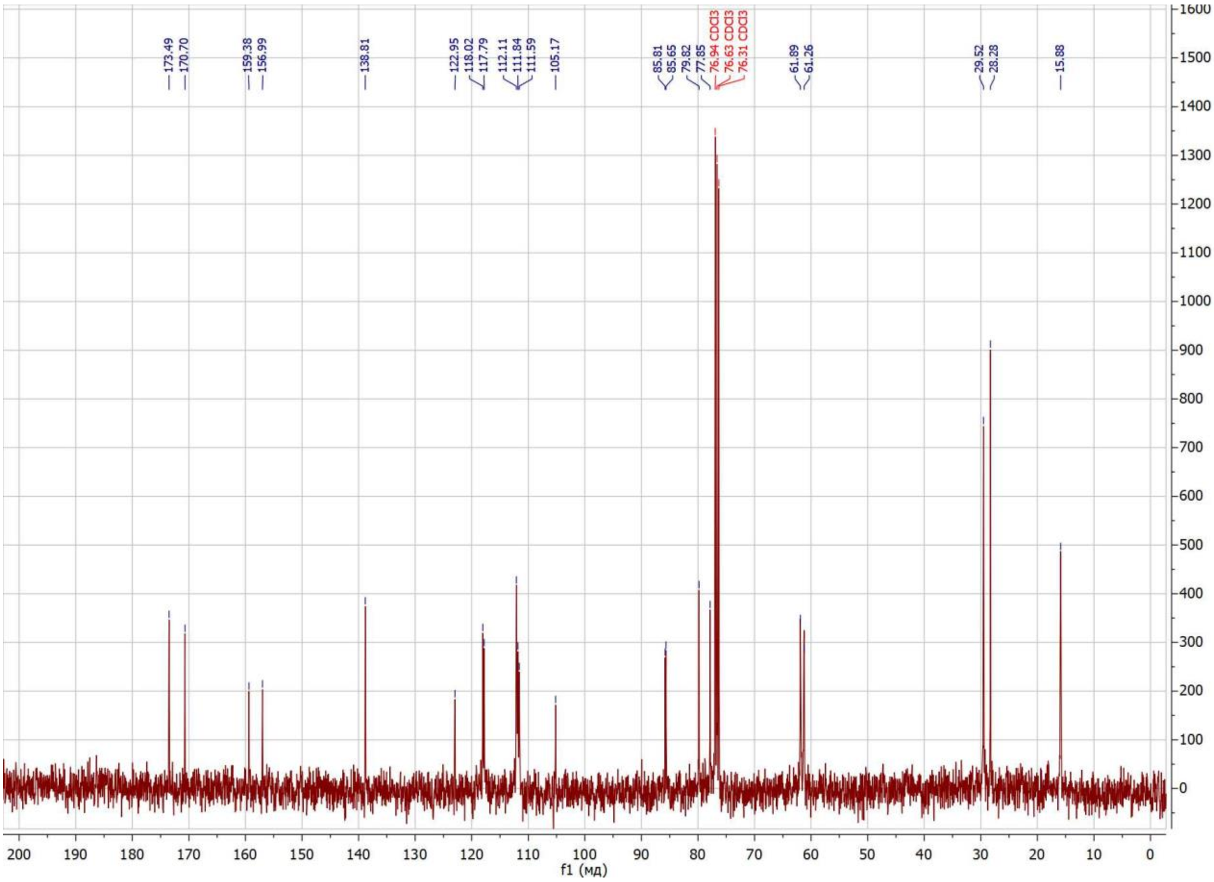

Figure S24. ^{31}P NMR spectrum of compound (**3h**)

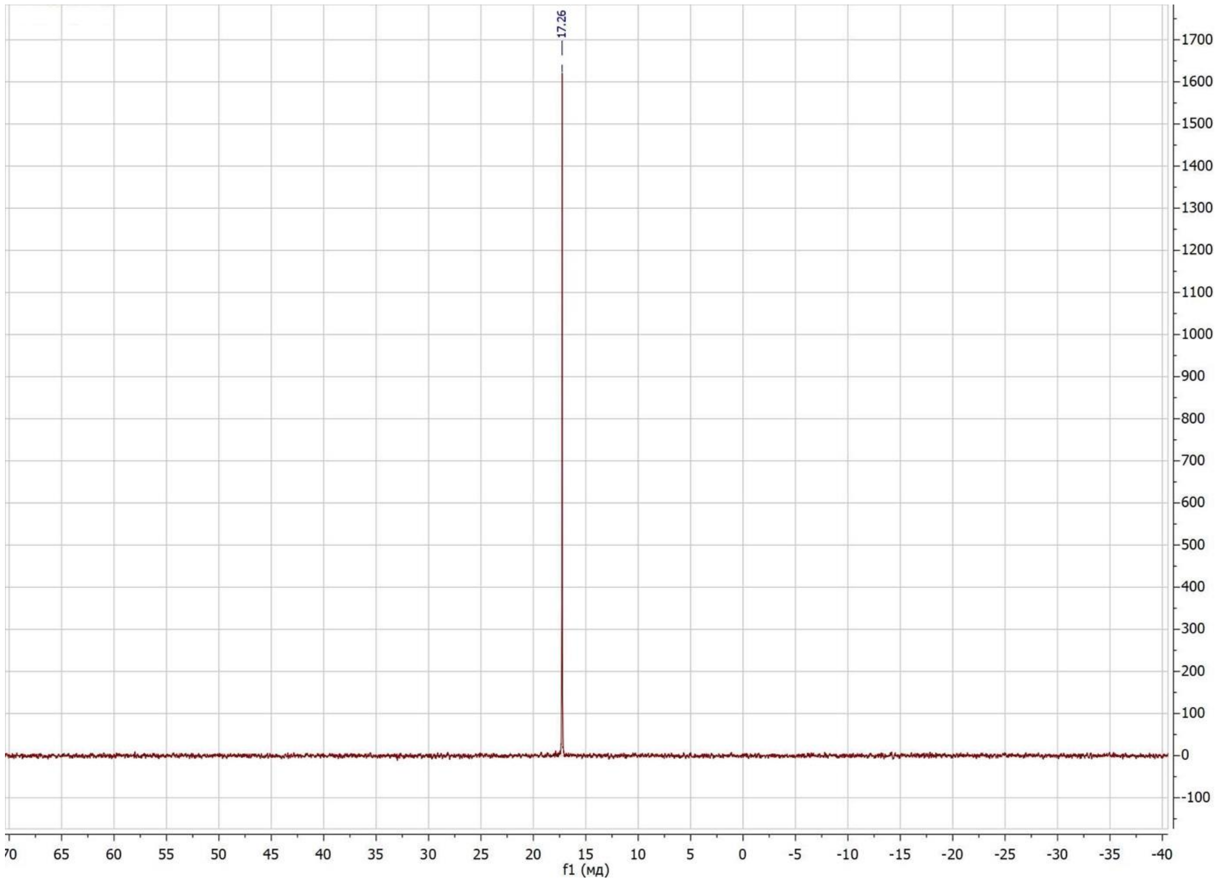

Figure S25. ^{1}H NMR spectrum of compound (**3i**)

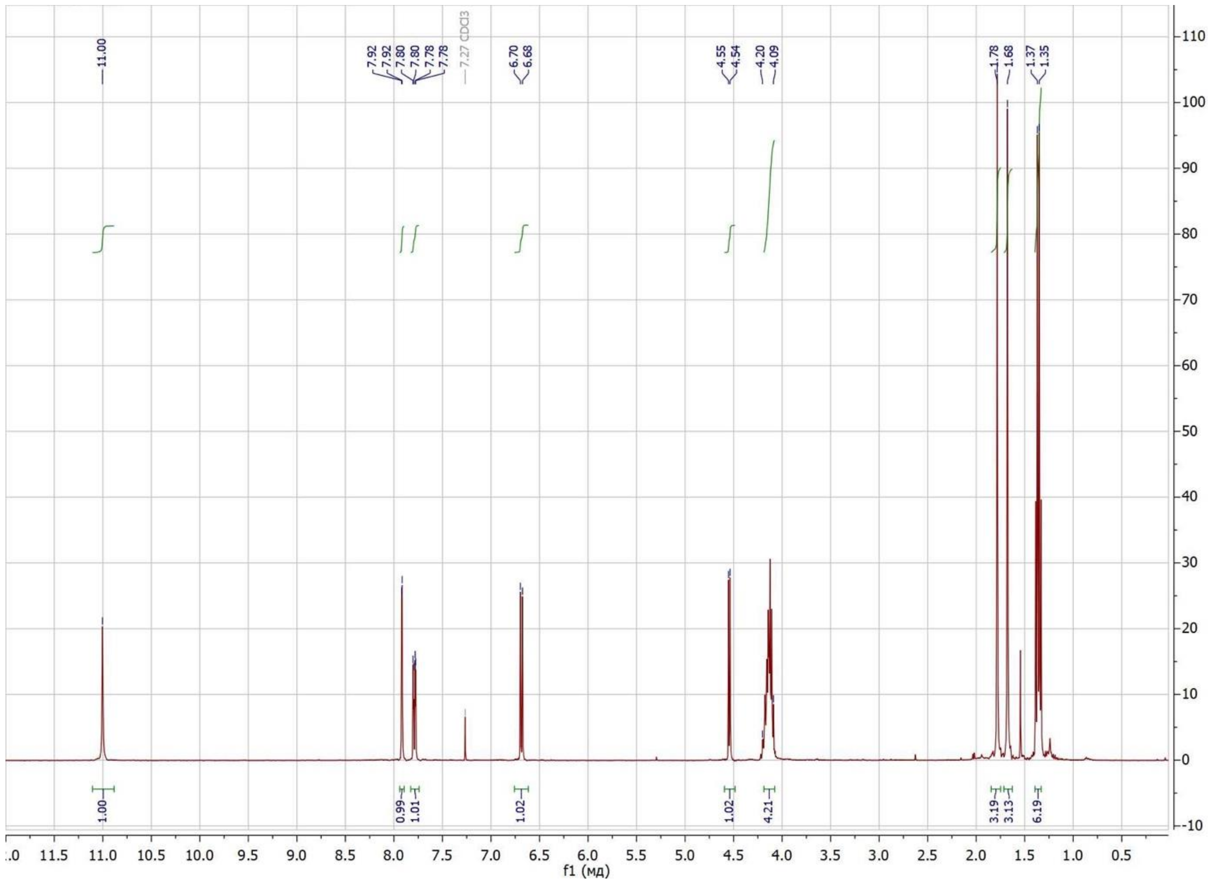

Figure S26. ^{13}C NMR spectrum of compound (**3i**)

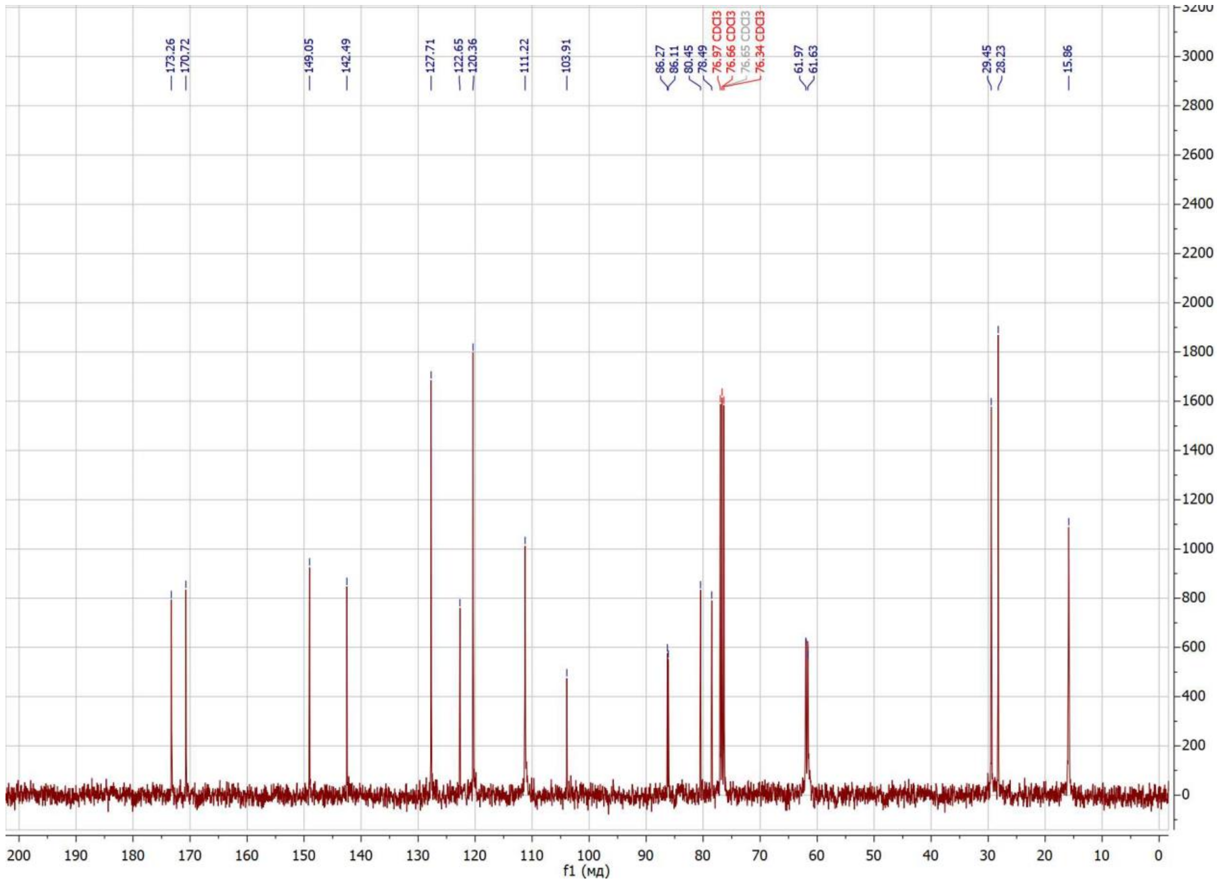

Figure S27. ^{31}P NMR spectrum of compound (**3i**)

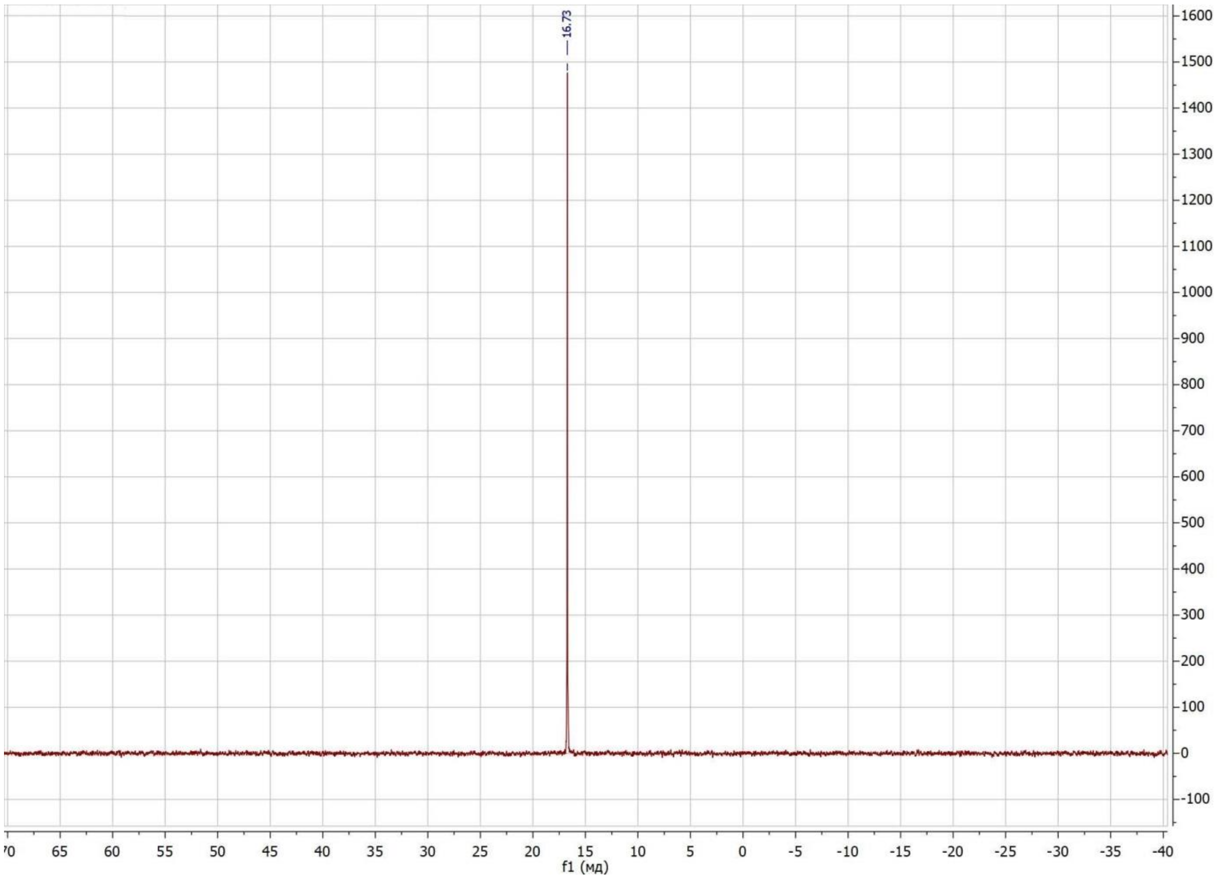

Figure S28. ^{1}H NMR spectrum of compound (**3j**)

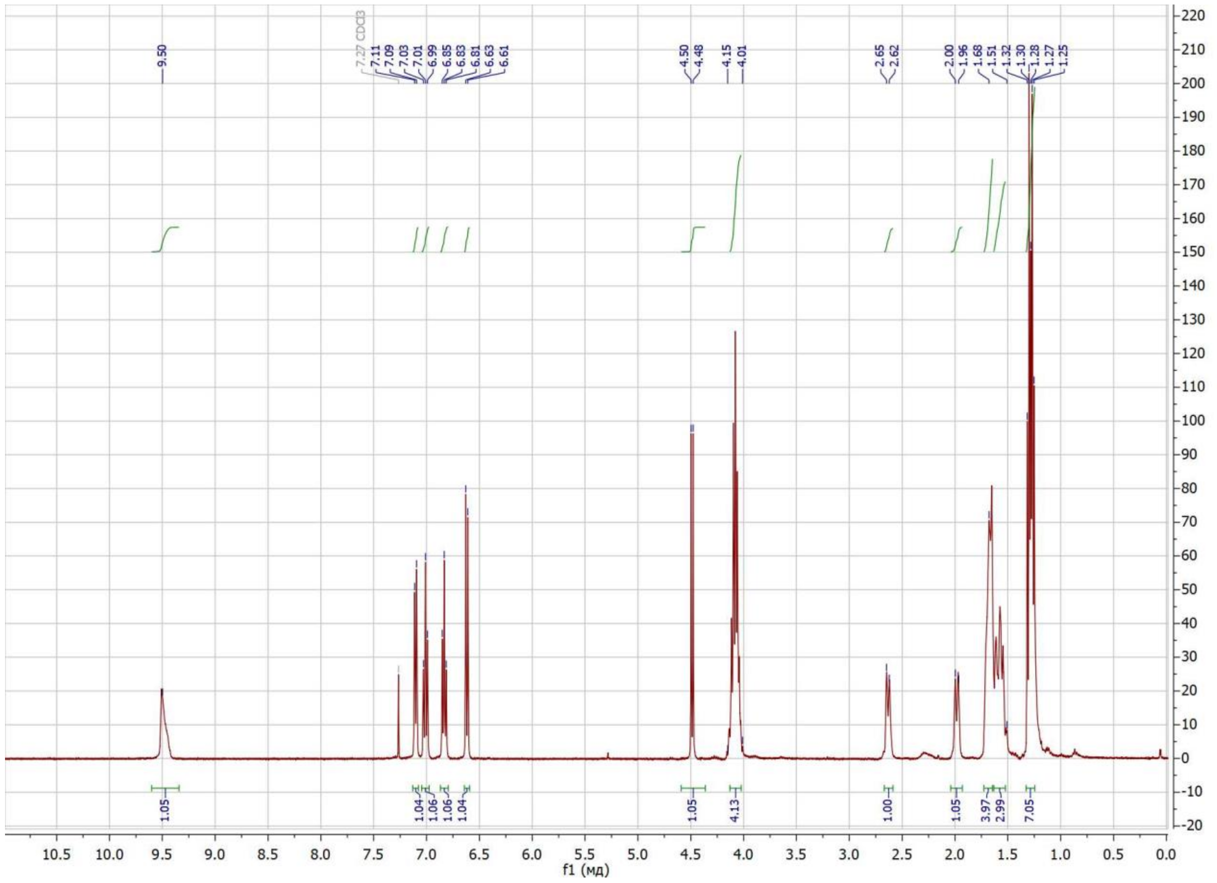

Figure S29. ^{13}C NMR spectrum of compound (**3j**)

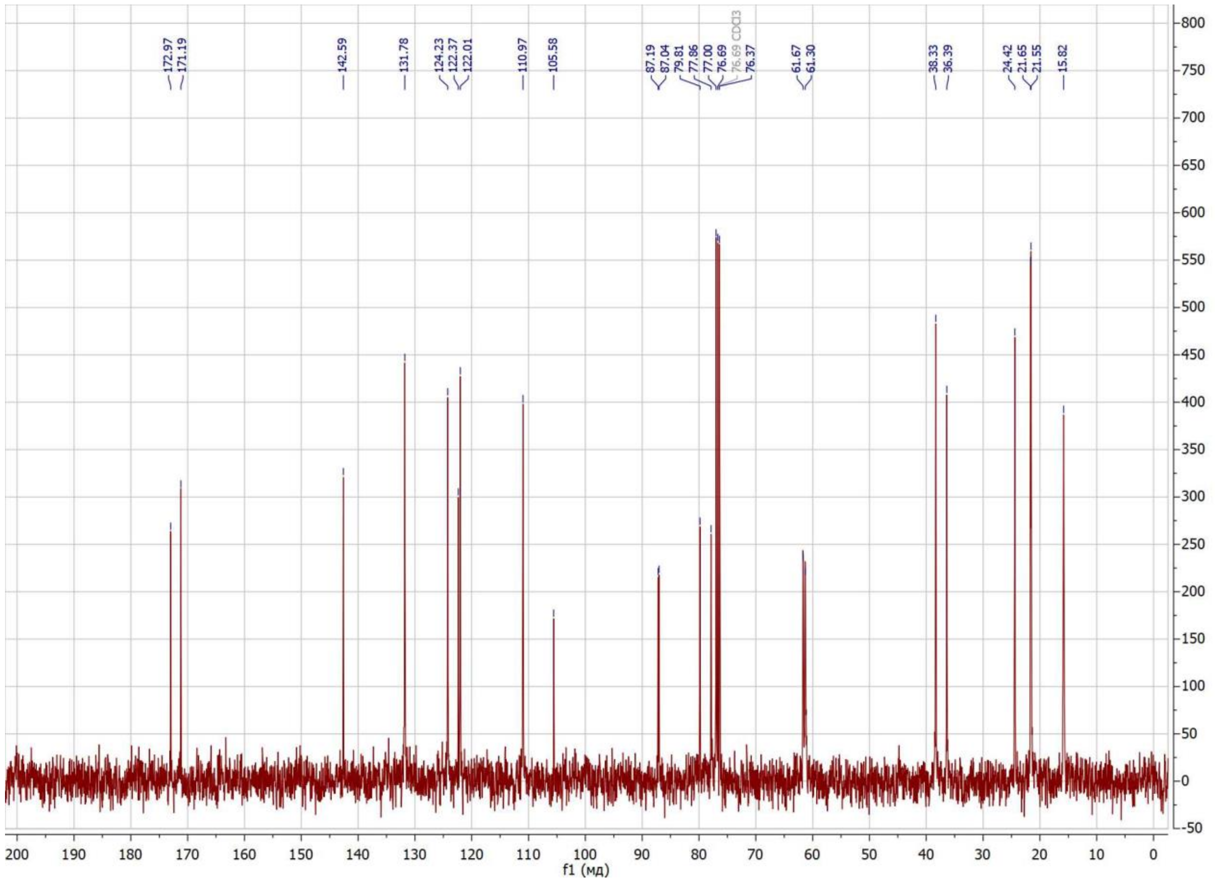

Figure S30. ^{31}P NMR spectrum of compound (**3j**)

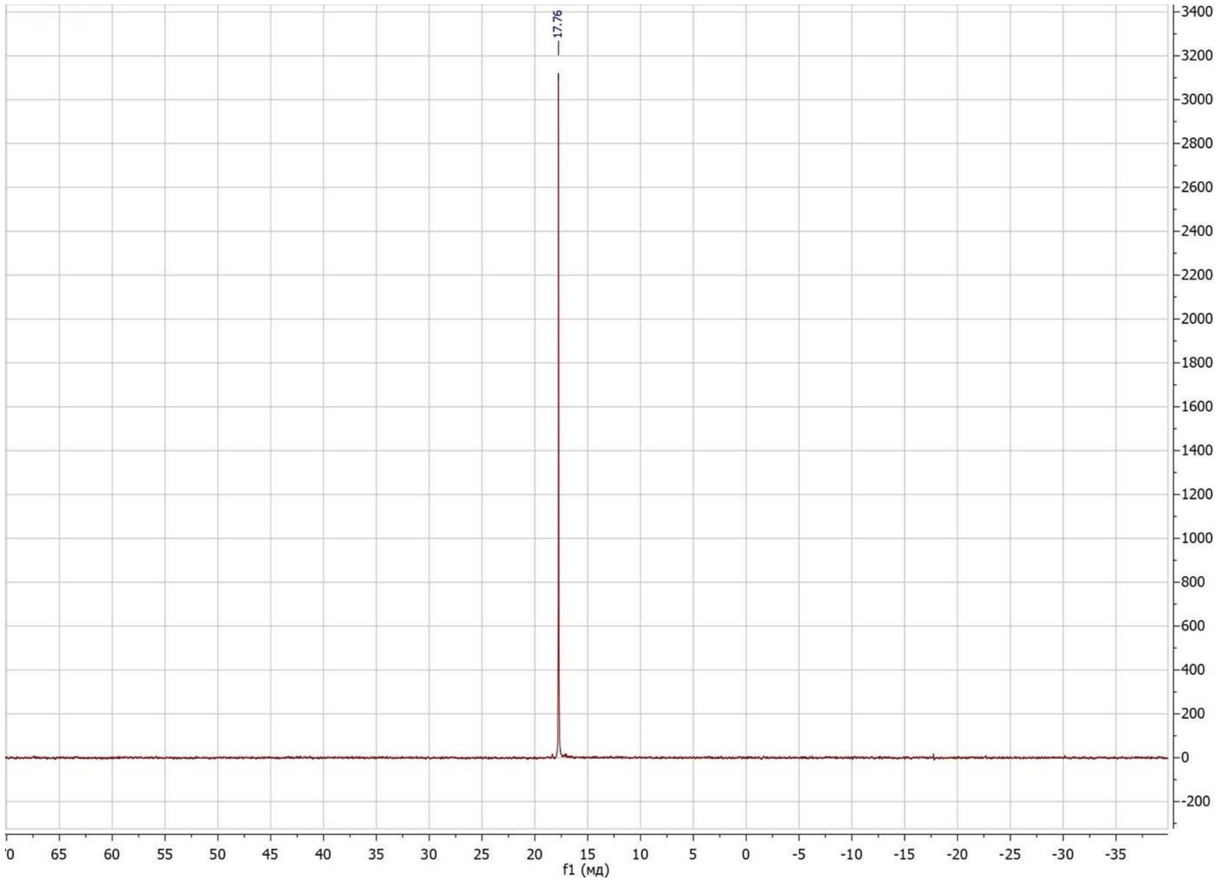

Figure S31. ^{1}H NMR spectrum of compound (**3k**)

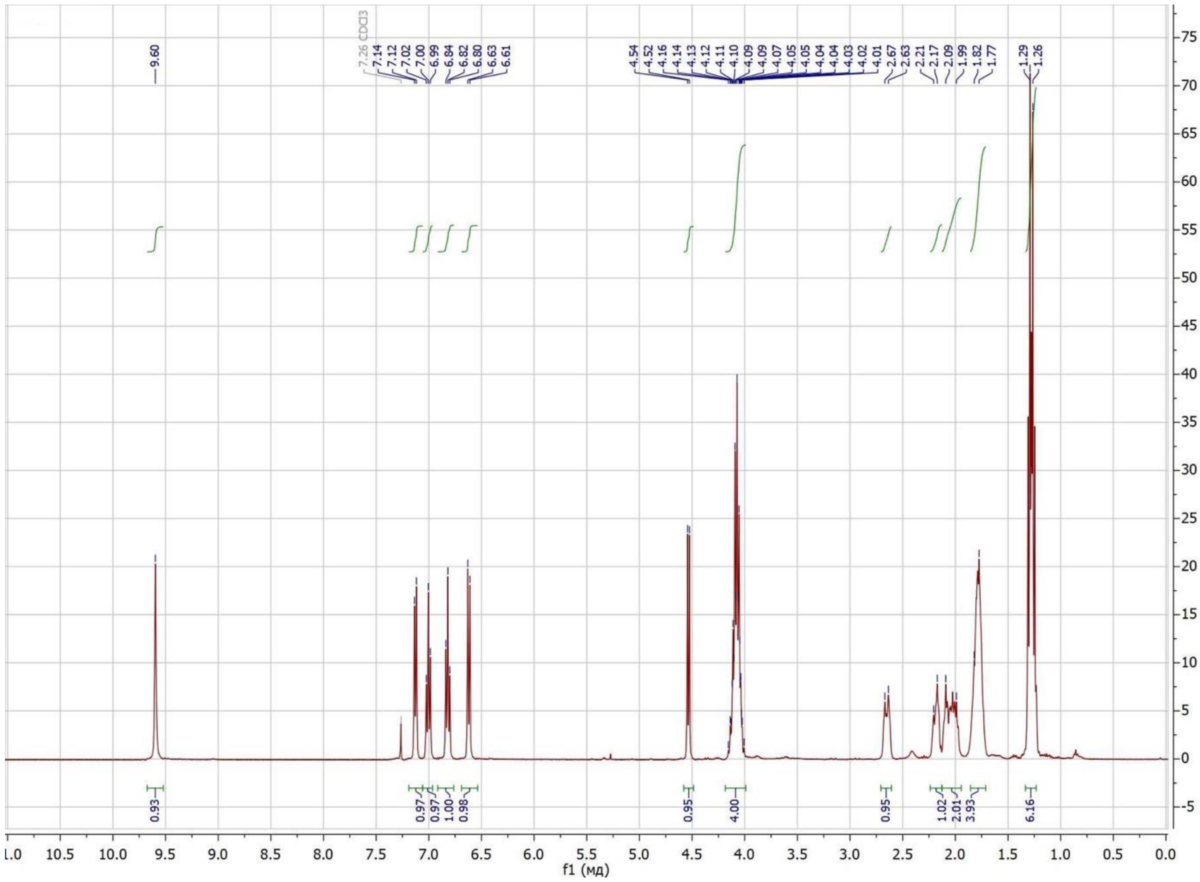

Figure S32. ^{13}C NMR spectrum of compound (**3k**)

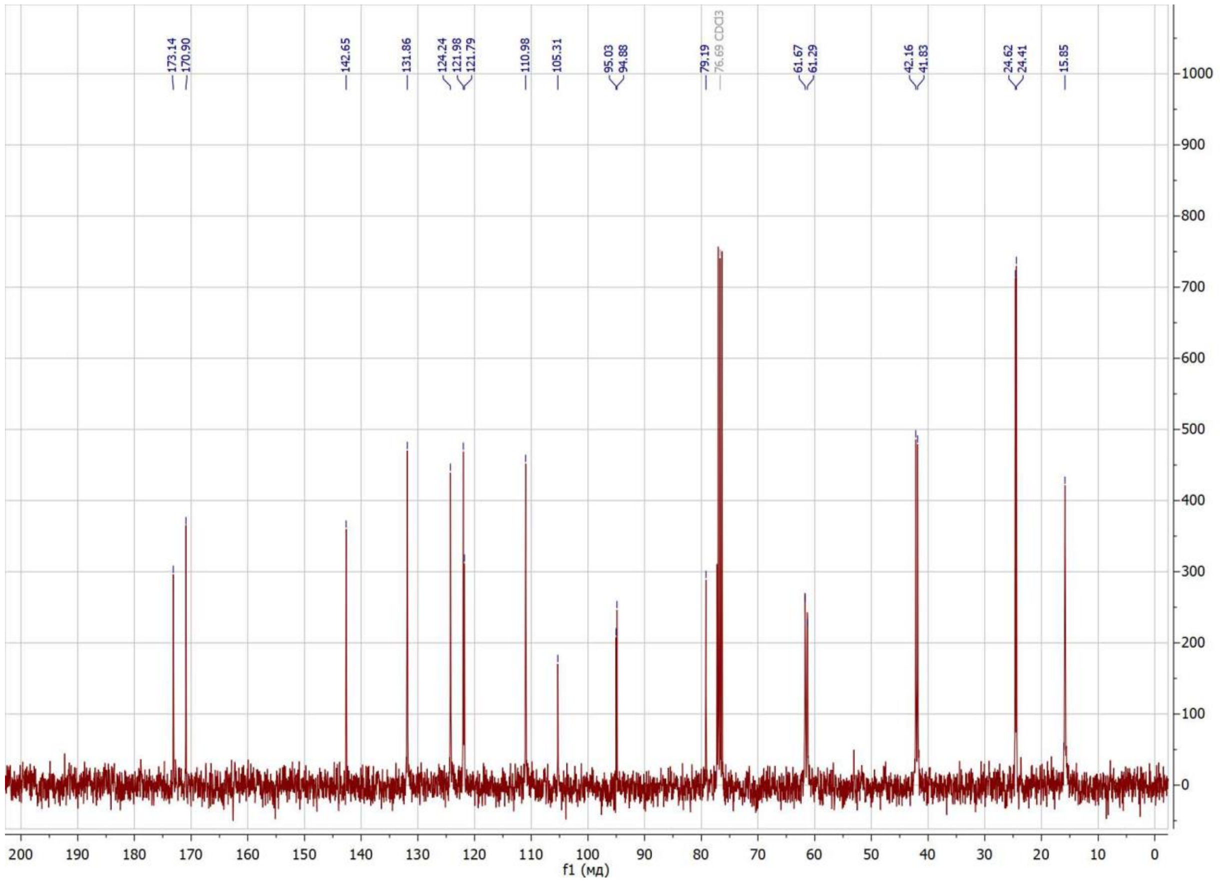

Figure S33. ^{31}P NMR spectrum of compound (**3k**)

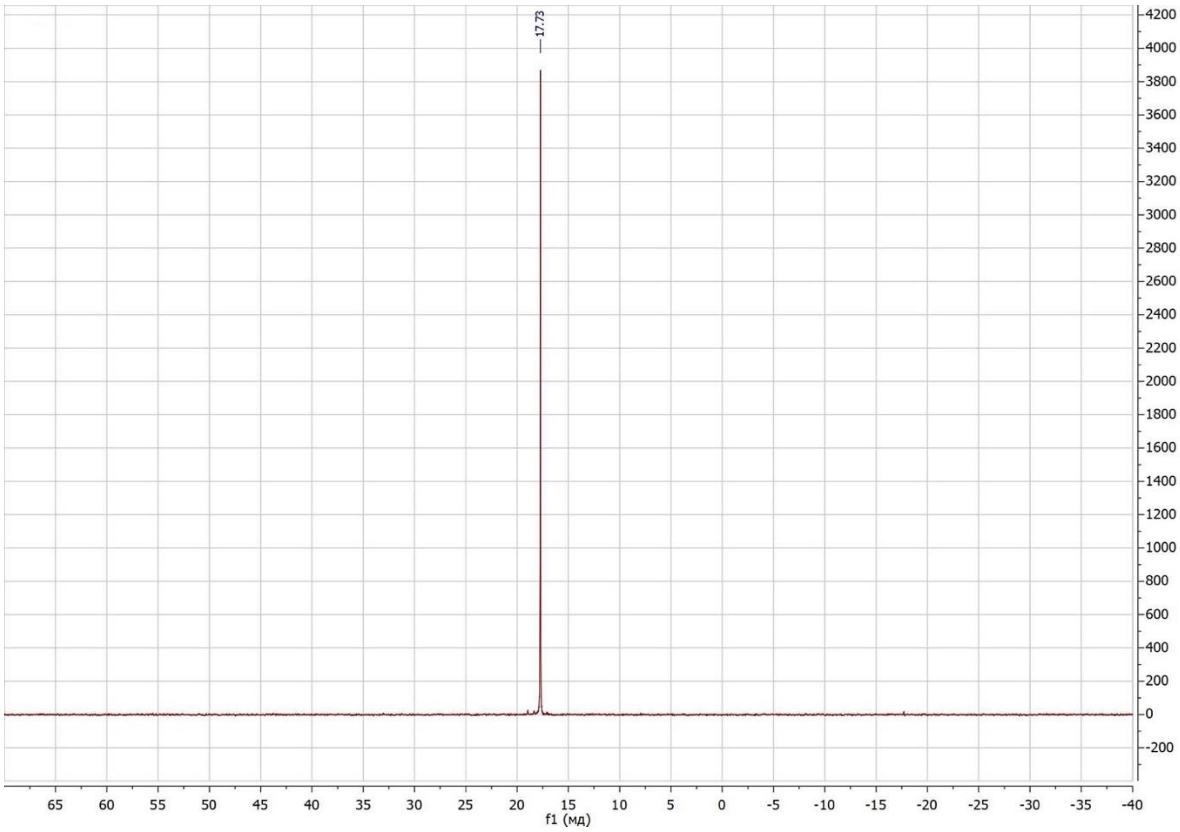

Figure S34. ^{1}H NMR spectrum of compound (**4a**)

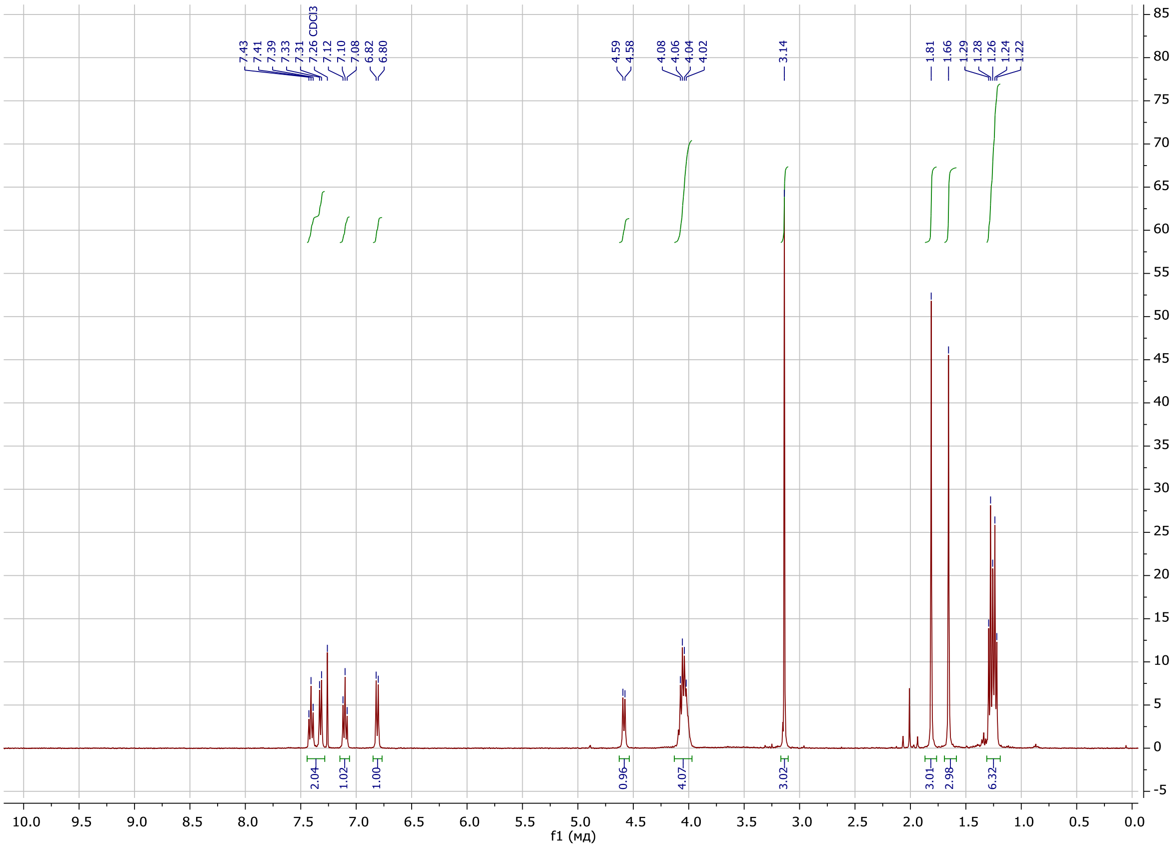

Figure S35. ^{13}C NMR spectrum of compound (**4a**)

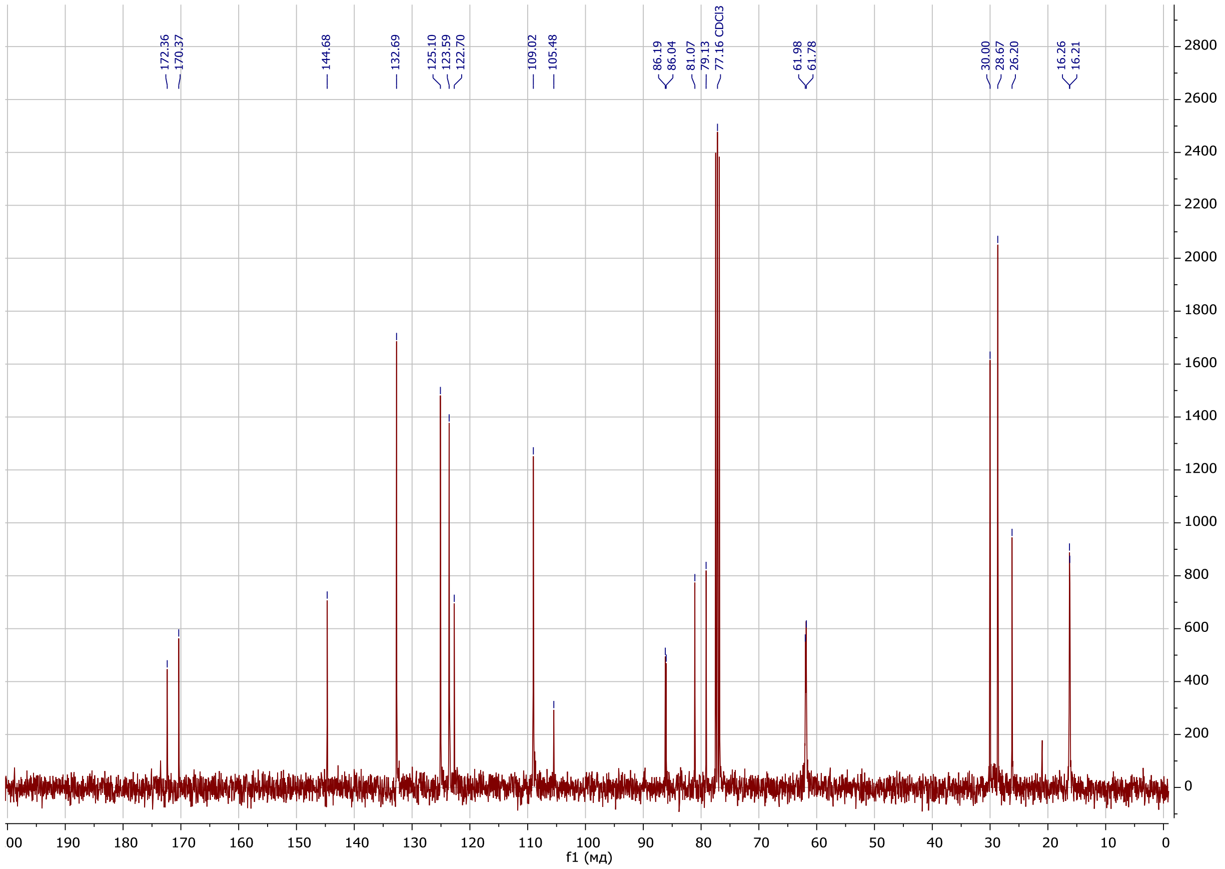

Figure S36. ^{31}P NMR spectrum of compound (**4a**)

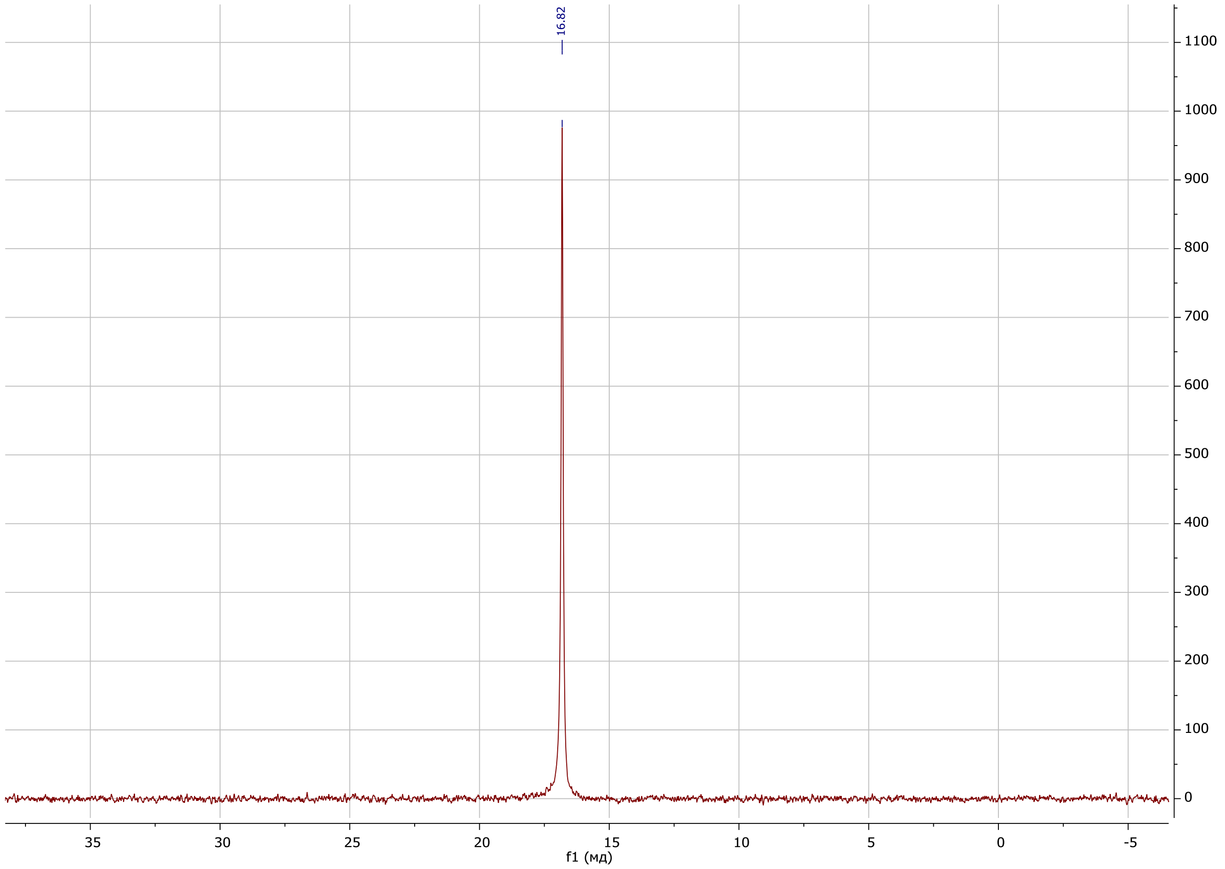

Figure S37. ^{1}H NMR spectrum of compound (**4b**)

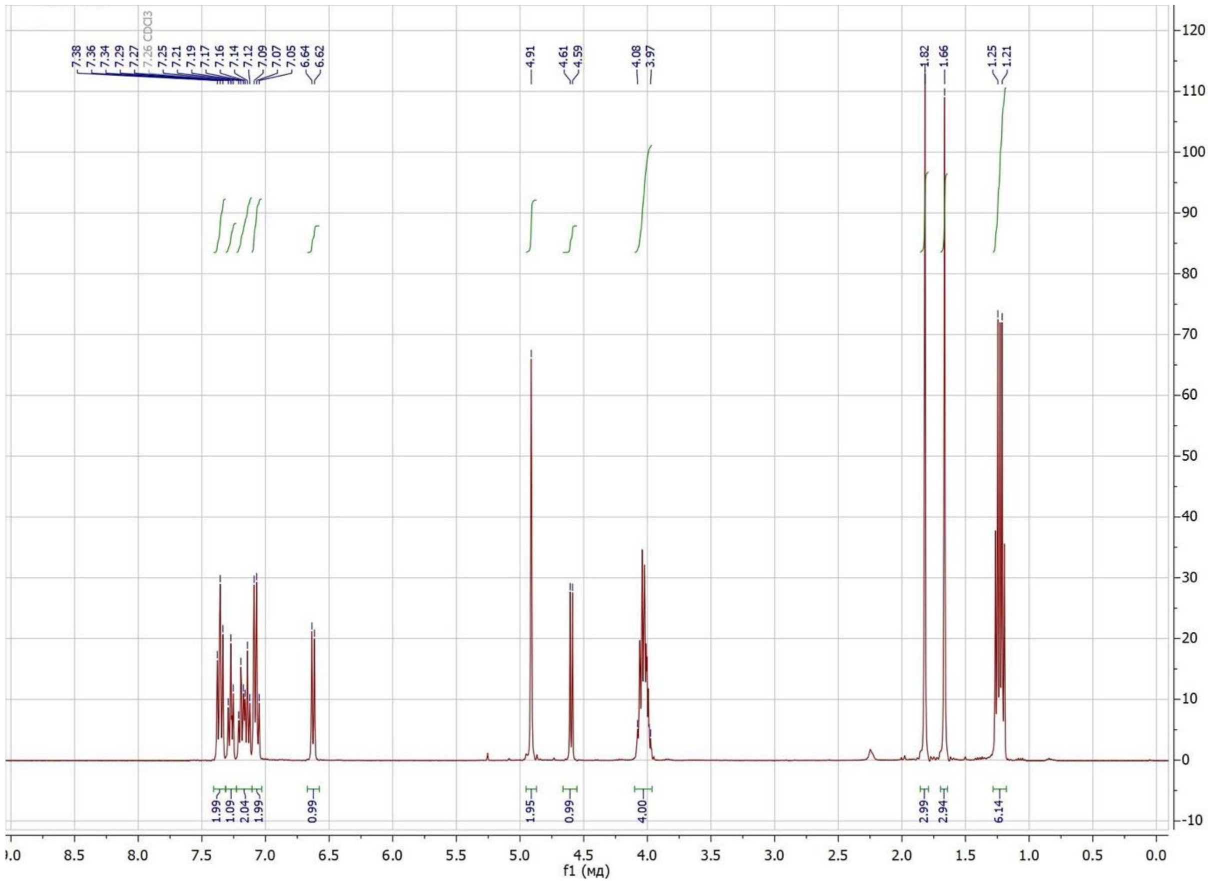

Figure S38. ^{13}C NMR spectrum of compound (**4b**)

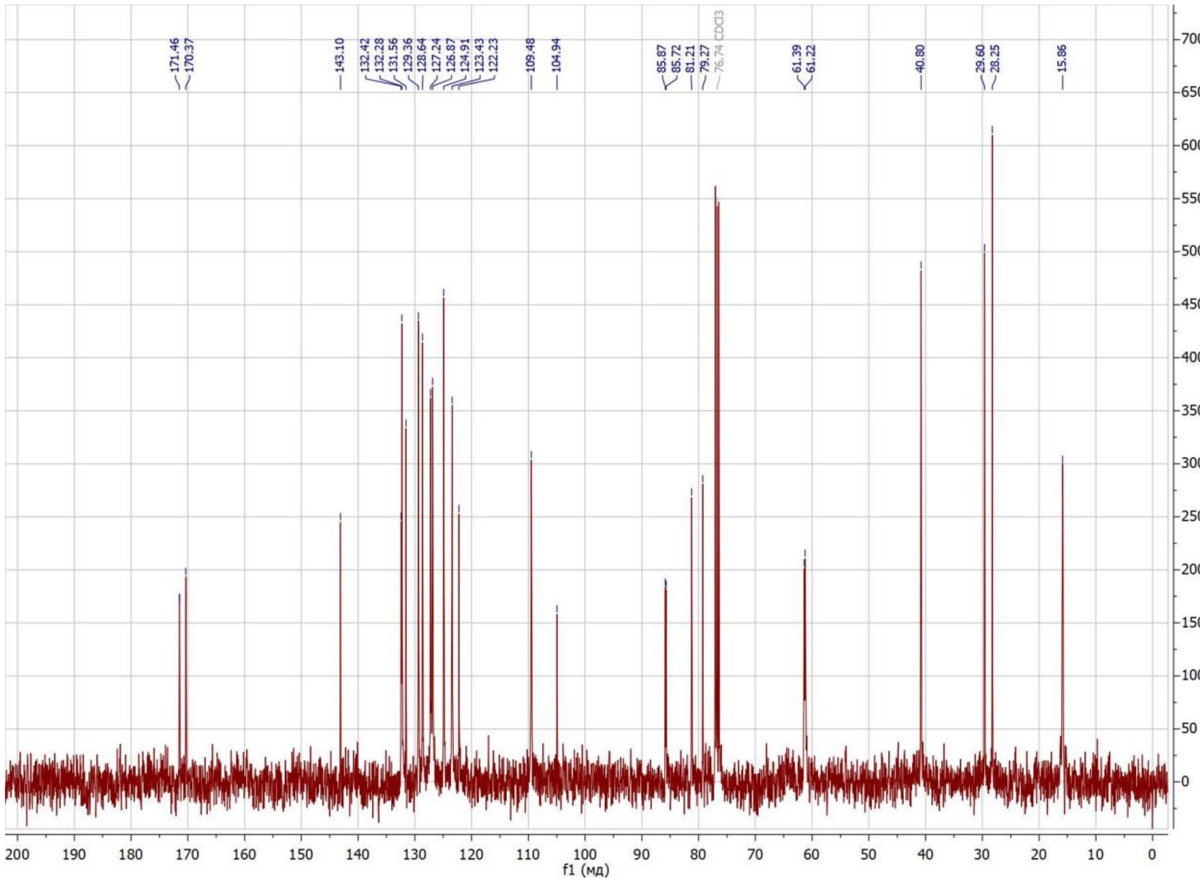

Figure S39. ^{31}P NMR spectrum of compound (**4b**)

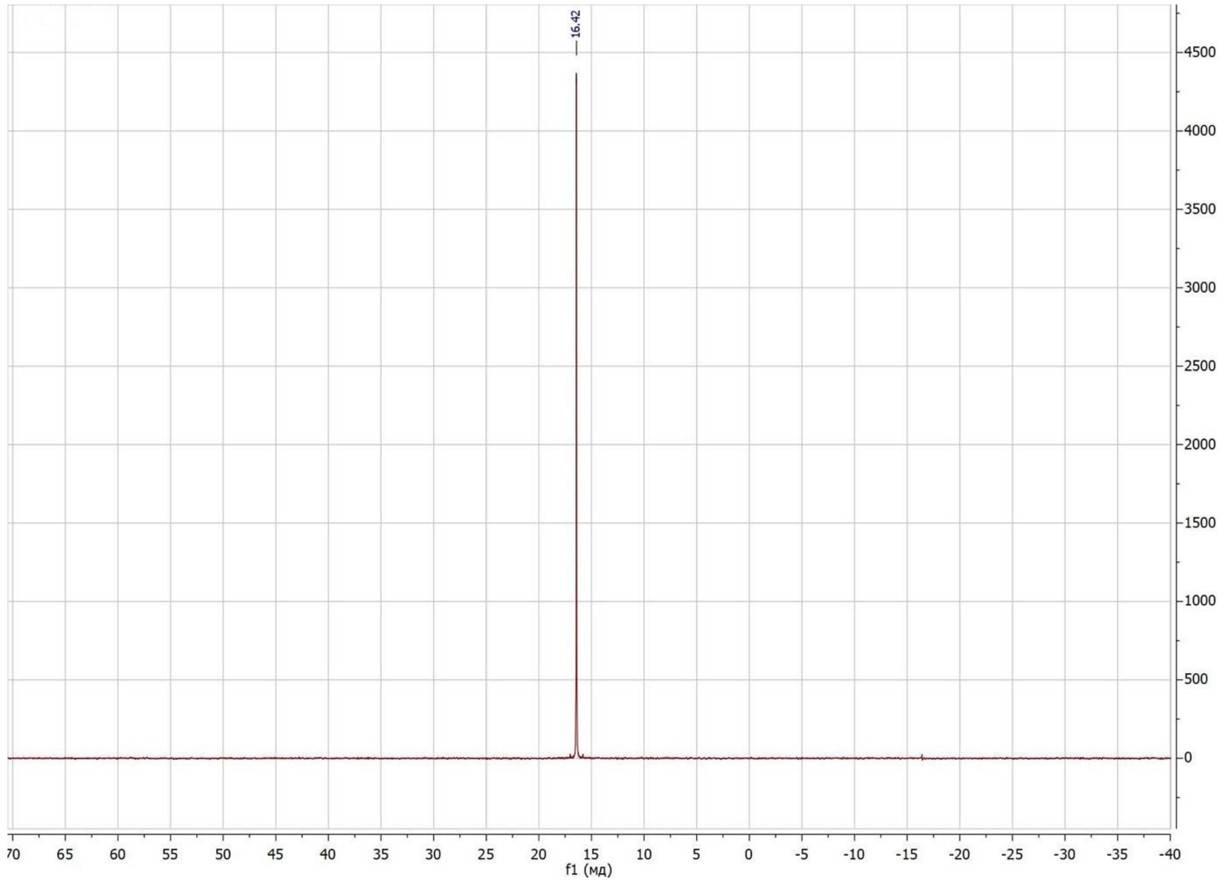

Figure S40. ^{1}H NMR spectrum of compound (**4c**)

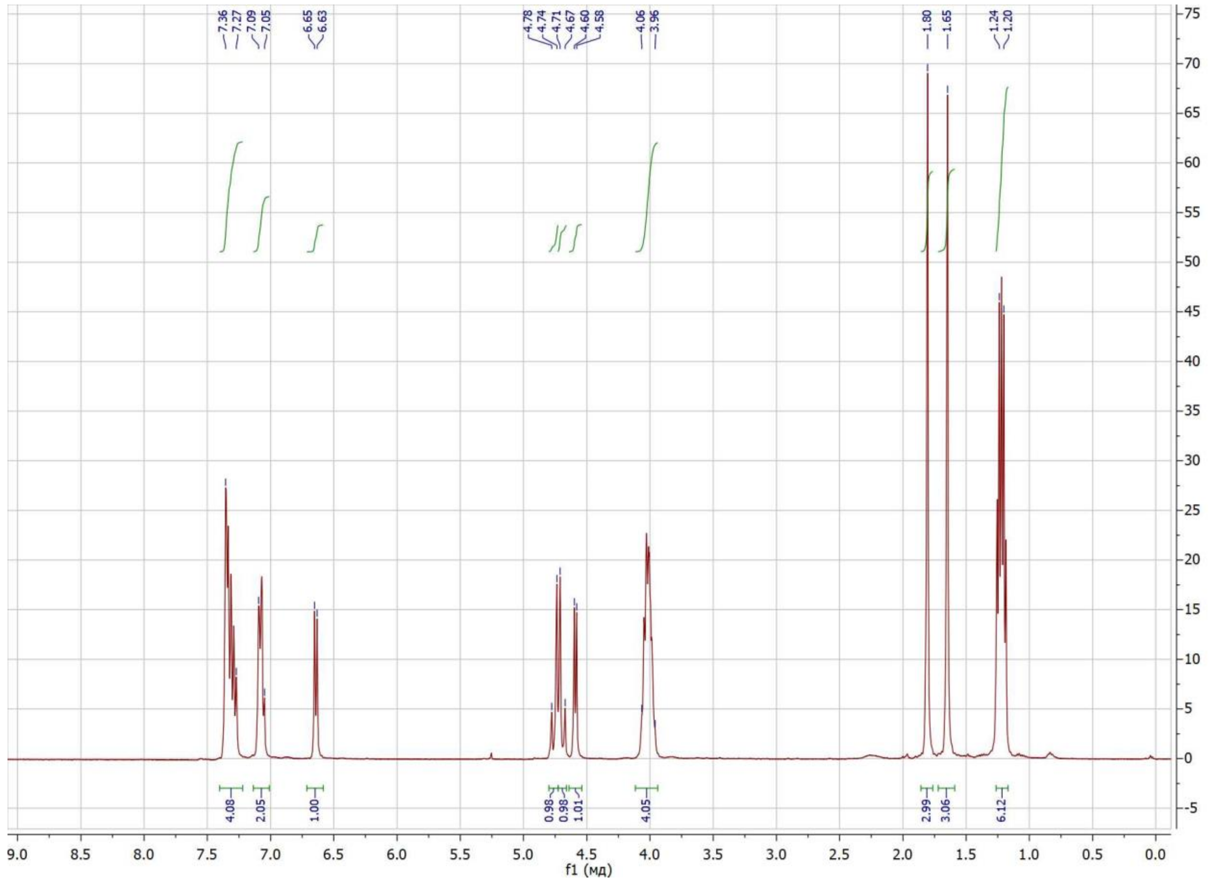

Figure S41. ^{13}C NMR spectrum of compound (**4c**)

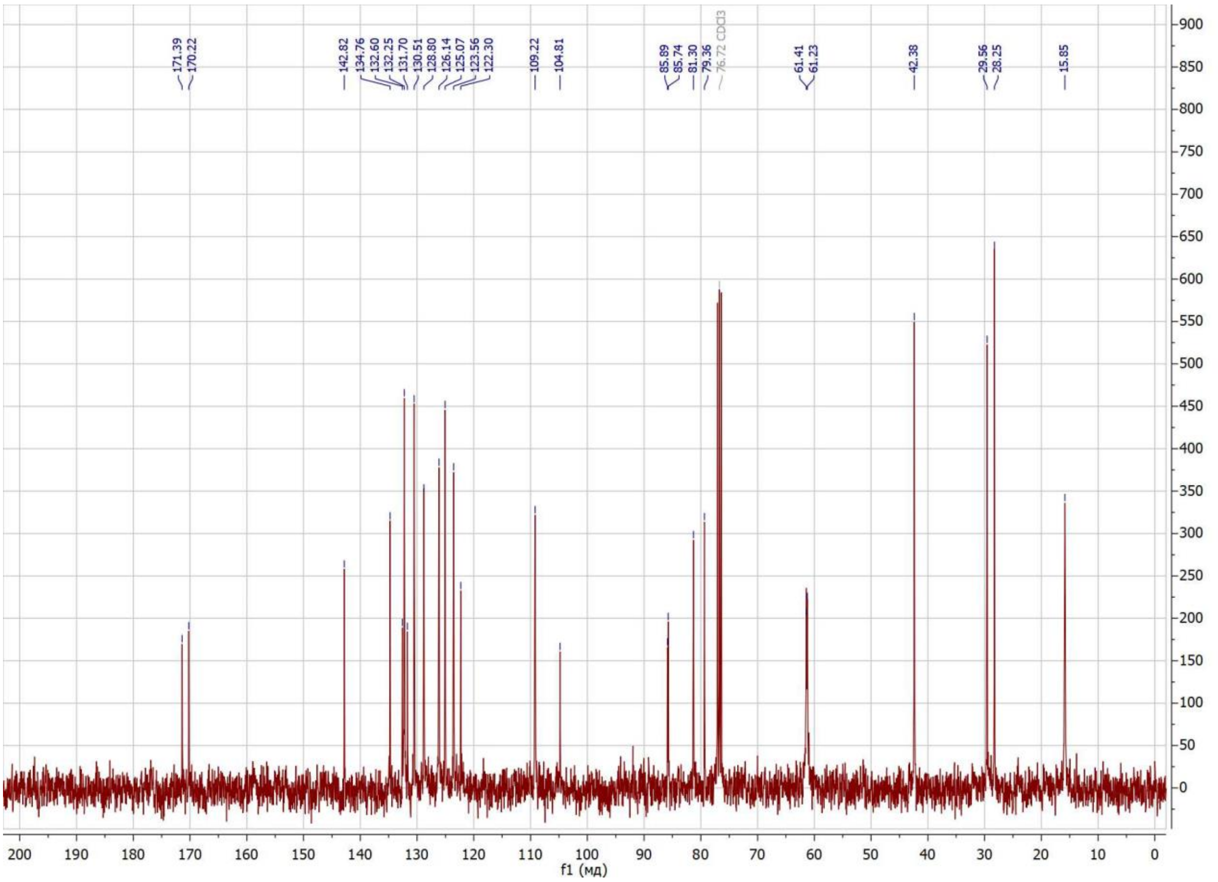

Figure S42. ^{31}P NMR spectrum of compound (**4c**)

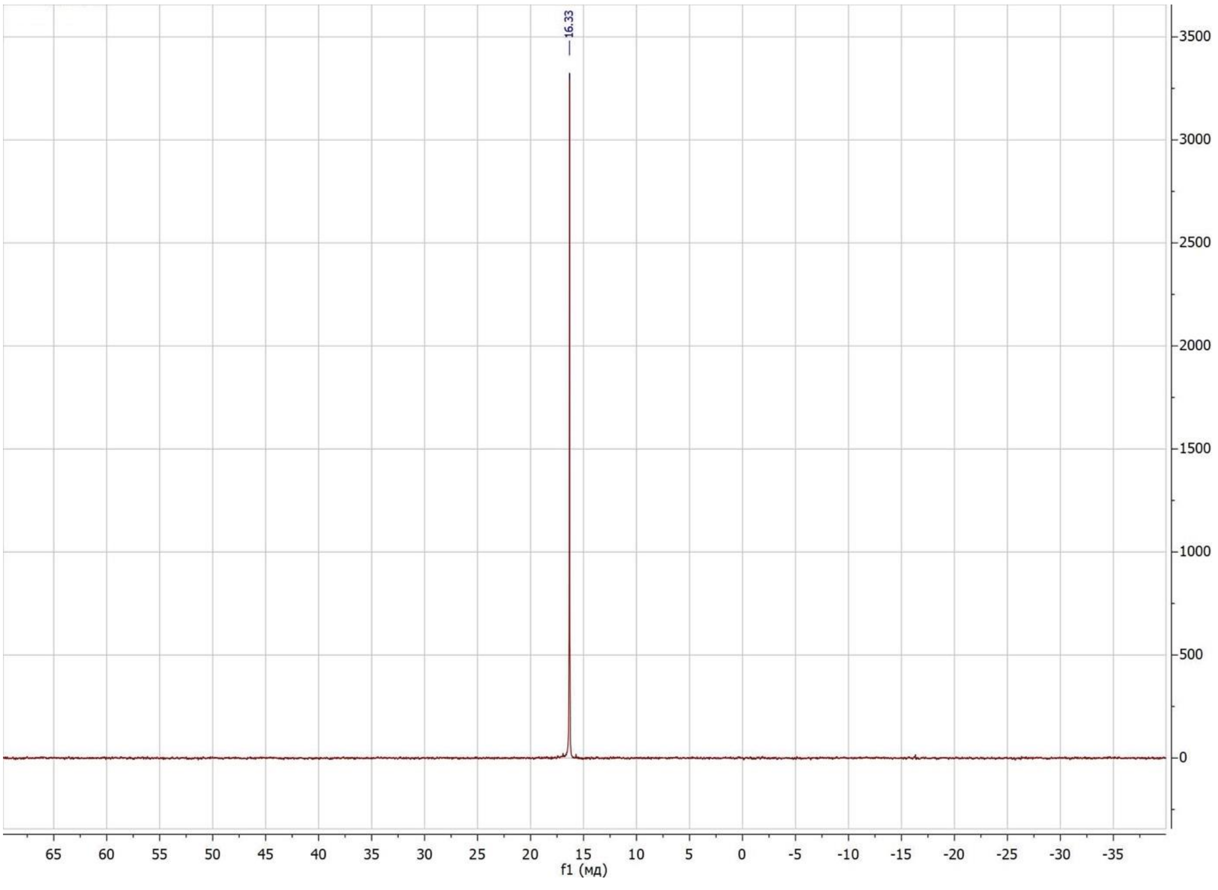

Figure S43. ^{1}H NMR spectrum of compound (**4d**)

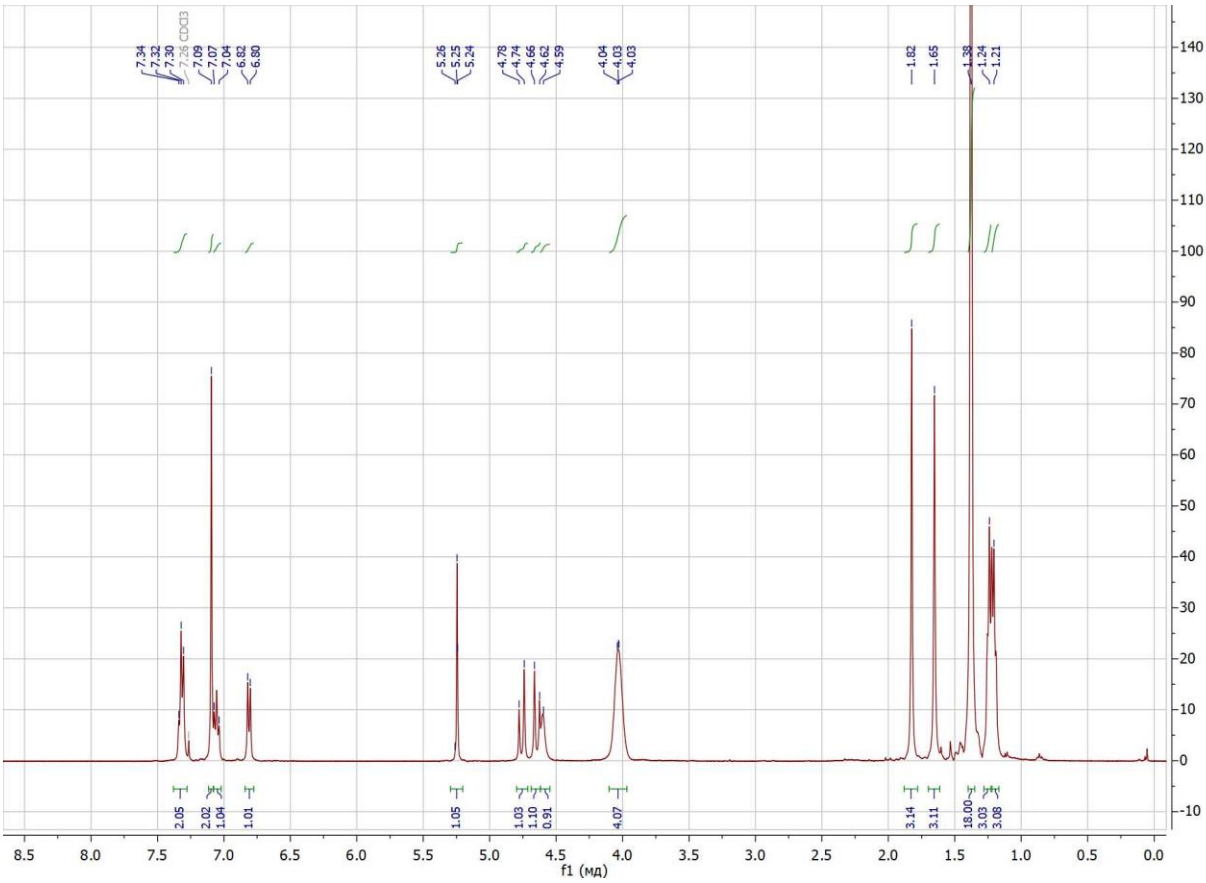

Figure S44. ^{13}C NMR spectrum of compound (**4d**)

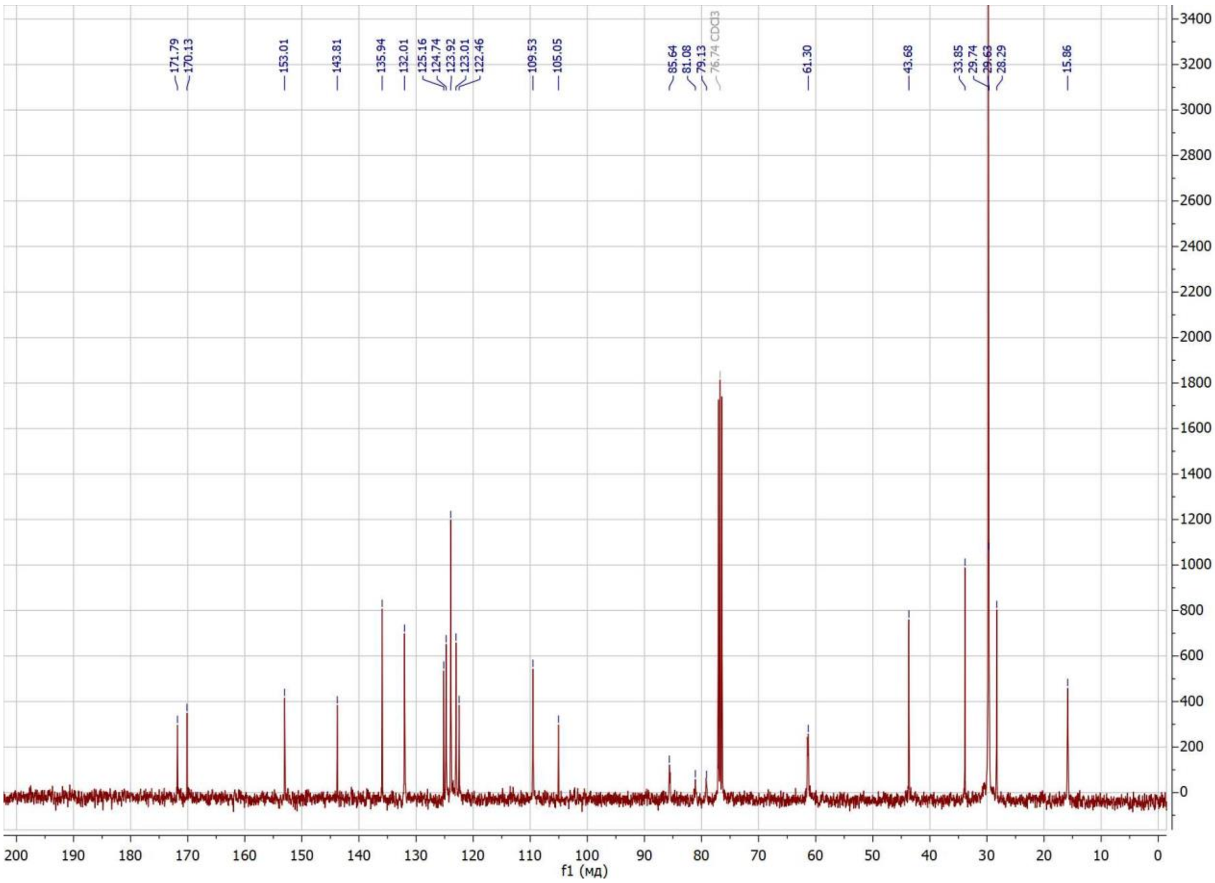

Figure S45. ^{31}P NMR spectrum of compound (**4d**)

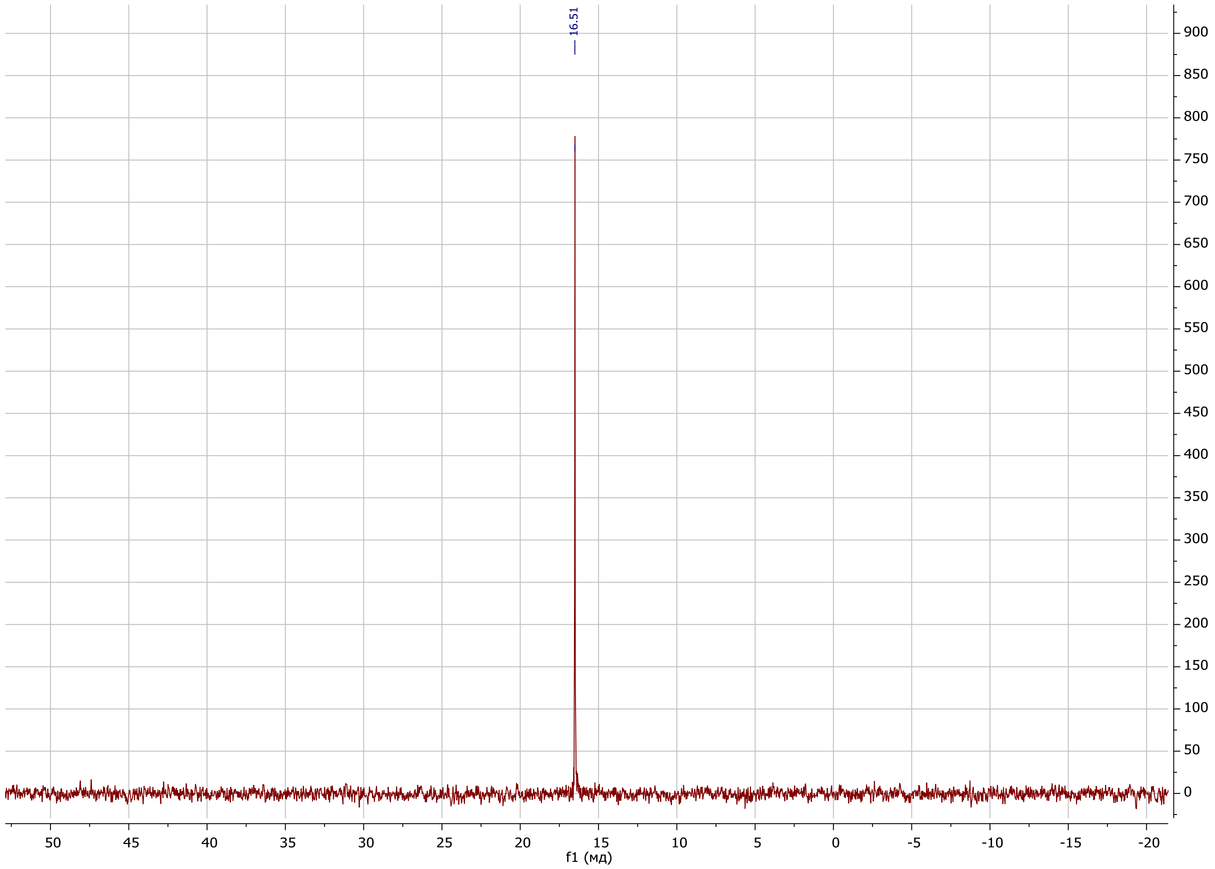

Figure S46. ^{1}H NMR spectrum of compound (**4e**)

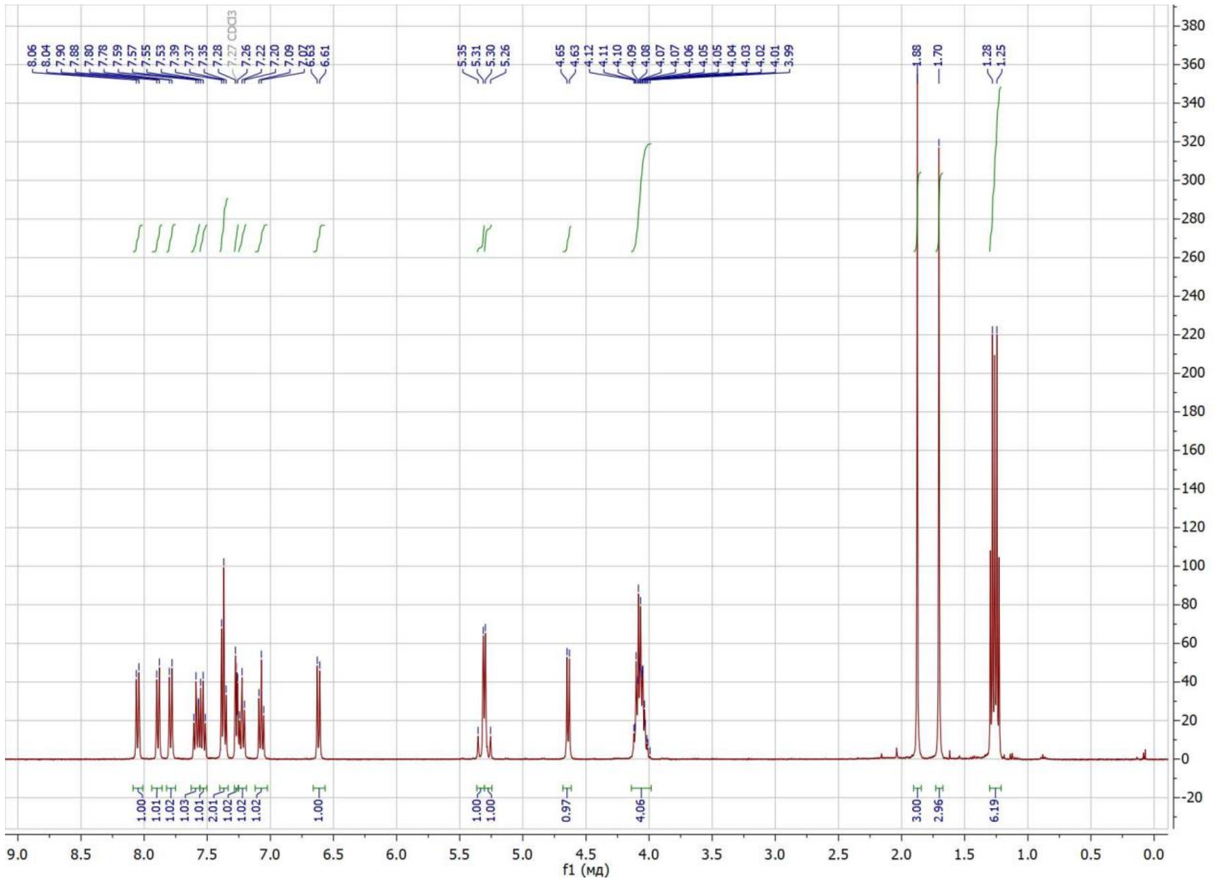

Figure S47. ^{13}C NMR spectrum of compound (**4e**)

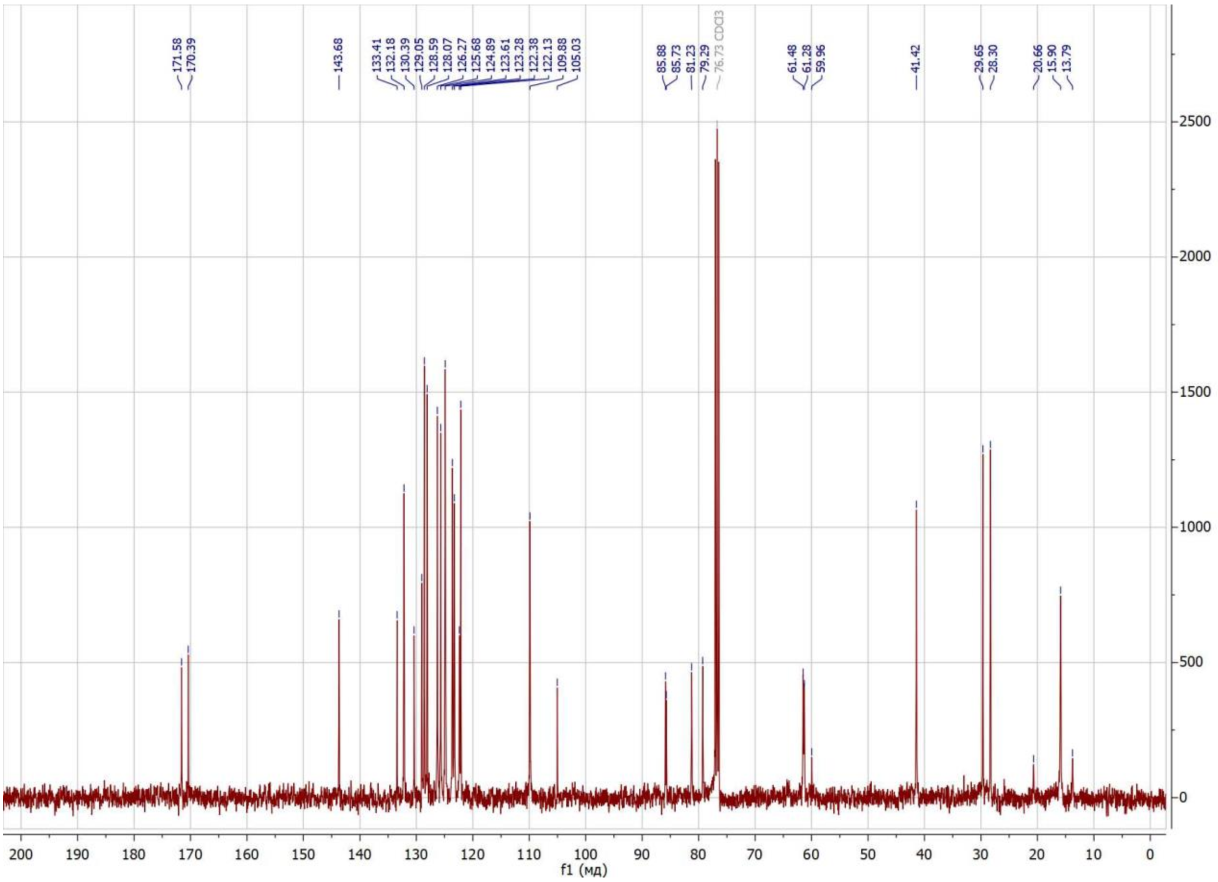

Figure S48. ^{31}P NMR spectrum of compound (**4e**)

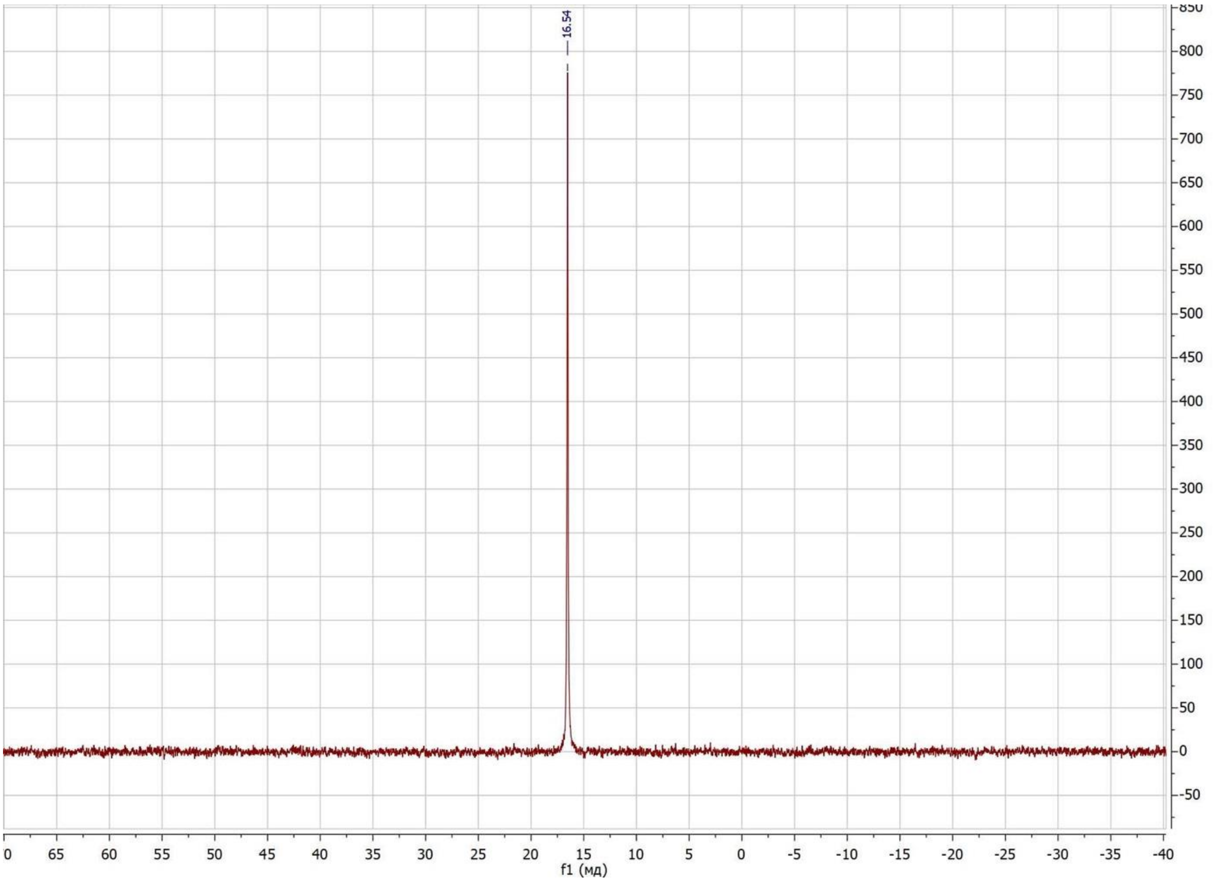

Supplement: Supplementary file 1 [file molecules-29-04764-s001.zip › molecules-3230710-supplementary.pdf]
